# Infertility prevalence and the methods of estimation from 1990 to 2021: a systematic review and meta-analysis

**DOI:** 10.1093/hropen/hoac051

**Published:** 2022-11-12

**Authors:** C M Cox, M E Thoma, N Tchangalova, G Mburu, M J Bornstein, C L Johnson, J Kiarie

**Affiliations:** Independent Consultant, Shoreview, MN, USA; Department of Family Science, School of Public Health, University of Maryland, College Park, MD, USA; Research, Teaching, and Learning, STEM Library, University of Maryland Libraries, College Park, MD, USA; Department of Sexual and Reproductive Health and Research (SRH), World Health Organization, Geneva, Switzerland; Division of Epidemiology, Ohio State University, Columbus, OH, USA; Department of Epidemiology and Community Health, School of Public Health, University of Minnesota, Minneapolis, MN, USA; Department of Sexual and Reproductive Health and Research (SRH), World Health Organization, Geneva, Switzerland

**Keywords:** time-to-pregnancy, subfertility, subfecundity, infecundity, infertility

## Abstract

**STUDY QUESTION:**

What is the contemporary prevalence of infertility in world populations and how do they differ by methodological and study characteristics?

**SUMMARY ANSWER:**

Pooled estimates of lifetime and period prevalence of 12-month infertility were 17.5% and 12.6%, respectively, but this varied by study population and methodological approach.

**WHAT IS KNOWN ALREADY:**

Infertility affects millions of individuals worldwide. Accurate measures of its magnitude are needed to effectively address and manage the condition. There are distinct challenges and variation in how infertility is defined and measured, limiting comparability of estimates across studies. Further research is needed to understand whether and how differences in methodological approaches and study characteristics account for heterogeneity in estimates.

**STUDY DESIGN, SIZE, DURATION:**

We conducted a systematic review and meta-analysis. Six electronic databases, websites of relevant organizations, and conference proceedings were systematically searched. Searches were limited to those published between 1 January 1990 and 11 March 2021, with no language restrictions.

**PARTICIPANTS/MATERIALS, SETTING, METHODS:**

Descriptive and random-effects meta-analysis models were used to examine range of estimates and generate estimates of pooled lifetime and period prevalence of 12-month infertility, respectively, among representative populations. Meta-regression using restricted maximum likelihood was applied to account for definitional and study characteristics and to obtain adjusted estimates. Risk of bias was assessed with a validated tool.

**MAIN RESULTS AND THE ROLE OF CHANCE:**

The search yielded 12 241 unique records of which 133 studies met the criteria for the systematic review. There were 65 and 69 studies that provided data for lifetime and period prevalence of 12-month infertility, respectively. Five methodological approaches were identified: prospective time-to-pregnancy (TTP) design, current duration design, retrospective TTP design, self-reported infertility measure and constructed infertility measure. Ranges for lifetime (3.3–39.7%) and period estimates (1.6–34.0%) were similar and wide even after accounting for methodological and study characteristics. Pooled estimates of lifetime and period prevalence were 17.5% (95% CI: 15.0, 20.3, n = 37 studies, *I*^2^ = 99.5%) and 12.6% (95% CI: 10.7, 14.6, n = 43 studies, *I*^2^ = 99.8%), respectively, with some variation in magnitude by region and methodological approach, but with most CIs overlapping.

**LIMITATIONS, REASONS FOR CAUTION:**

Pooled estimates generated from meta-analysis were derived from 12-month infertility prevalence estimates that were heterogeneous across different domains, even after adjusting for definitional and study characteristics. The number of studies was small for certain strata from which pooled estimates were derived (e.g. there were only two studies for lifetime prevalence in Africa).

**WIDER IMPLICATIONS OF THE FINDINGS:**

While findings show a high prevalence of infertility globally and regionally, it also reveals variation in measures to ascertain and compare infertility prevalence. More systematic and comprehensive collection of data using a consistent definition is needed to improve infertility prevalence estimates at global, regional and country-levels.

**STUDY FUNDING/COMPETING INTEREST(S):**

This work was supported by the World Health Organization. The authors have no conflicts of interest.

**REGISTRATION NUMBER:**

PROSPERO CRD42020211704

WHAT DOES THIS MEAN FOR PATIENTS? Infertility is a condition that impacts millions of people throughout the world, often with devastating consequences. It is defined broadly as a disease of the male and/or female reproductive system based on a prolonged period of time in which pregnancy or live birth is not reached despite unprotected, sexual intercourse; however, the precise definition and methods used to estimate its frequency varies across research studies. This variation makes it difficult to understand and estimate the magnitude of the problem with and across settings, which impacts prevention and treatment efforts for infertility.

To explore this issue further, we identified and reviewed all studies that estimated the prevalence of infertility between 1990 and 2021. We first described all of the studies that were identified during our search regardless of the definition of infertility that they used. We then narrowed our focus to those studies that defined infertility based on a 12-month duration because this is a useful measure for understanding clinical needs of individuals and couples with infertility and is the definition adopted by the World Health Organization (WHO). Accordingly, we combined the estimates that measured infertility based on a duration of 12 months to create pooled estimates of the prevalence of infertility globally and regionally as well as by other characteristics of interest. However, given the variation in how researchers defined and measured infertility, the estimates are imperfect. To generate estimates that are more accurate and comparable, researchers must improve how data are collected and analyzed. Shifting to a more systematic approach to measuring and defining the prevalence of infertility will improve our understanding of global, regional and country-level infertility prevalence and, subsequently, our ability to help those who experience it.

## Introduction

Addressing infertility is an important component of sexual and reproductive health and rights (SRHR) but has not been a major focus in the global SRHR agenda. Much more should be done to improve the prevention, management and treatment of infertility worldwide (Gerrits *et al.*, 2017; Starrs *et al.*, 2018). Infertility is thought to affect millions of individuals and couples worldwide often with devastating societal and health consequences, including social stigma, economic hardship, and poor physical and mental wellbeing (Thoma *et al.*, 2021). Given the global burden of infertility, there is an urgent need for improved efforts to address it. Understanding the magnitude of infertility is critical for monitoring, assessing and improving equitable access to quality fertility care services, addressing risk factors for and consequences of infertility, and safeguarding individual rights, freedom and ability to decide the number, spacing and timing of children.

Despite the importance of understanding the magnitude of infertility, there is considerable variability in its estimation at the population level, which, as many researchers have noted, complicates comparisons across studies, populations and time (Thonneau and Spira, 1990; Schmidt and Münster, 1995; Guzick and Swan, 2006; Olive and Pritts, 2006; Gurunath *et al.*, 2011; Stanford, 2013; Thoma, 2015). The precise number of individuals or couples affected is unknown and estimates range from 48.5 million couples globally (Mascarenhas *et al.*, 2012b) to 186 million ever-married women in developing countries alone (Rutstein and Shah, 2004). One of the most extensive analyses of infertility prevalence used data from 277 reproductive health surveys and Bayesian hierarchical modeling to generate age-standardized estimates for 190 countries and territories using a duration of 5 years or more to define infertility (Mascarenhas *et al.*, 2012b) and concluded that 1.9% of women exposed to the risk of pregnancy experienced primary infertility, defined as the inability to have any live birth and 10.5% experienced secondary infertility, defined as the inability to have an additional live birth. A 2007 literature review of 25 population surveys found that the prevalence of infertility when defined by a duration of 12 months or more ranges from 3.5% to 16.7% in more developed nations and from 6.9% to 9.3% in less-developed nations (Boivin *et al.*, 2007). Another review and meta-analysis of 52 studies reported that infertility had a mean prevalence of 10% worldwide, with pooled prevalences being lowest and highest for the continents of Australia and Africa, respectively (Moghaddam *et al.*, 2016).

Distinct challenges related to the definition and measurement of infertility exist. Unlike other types of conditions, infertility is defined by the absence of an event (i.e. not getting pregnant or having a live birth) between two people, usually after a defined period of time. The WHO adopts the definition of ‘the failure to achieve a pregnancy after 12 months or more of regular unprotected sexual intercourse’ (World Health Organization, 2018). This time frame is generally consistent with clinical recommendations to begin fertility care and diagnostic testing. Other definitions of infertility may include longer durations, such as 24 or 60 months (Gurunath *et al.*, 2011), or incorporate non-duration-based definitions to include health conditions that warrant infertility services or relationship factors, such as single persons or same-sex couples (Zegers-Hochschild *et al.*, 2017).

Prior systematic reviews have concluded that definitional and methodological considerations influence comparisons across populations. This was first documented by Schmidt and Münster (1995) in their systematic review of infertility and involuntary infecundity studies in high-income countries (HICs) between 1970 and 1992. A more recent systematic review by Gurunath *et al.* (2011) included both high and low-to-middle income countries and similarly found a wide range in global infertility prevalence estimates. They attributed this heterogeneity to variation in defining the numerator and denominator and other study characteristics. Limiting their findings to 12-month estimates did not fully account for this variation, a finding consistent with an earlier (non-systematic) review by Boivin *et al.* (2007). In all of these reviews, conclusions regarding true differences across populations were obscured due to variation in how infertility measures were operationalized, including study design, survey instruments and analytic method (Dyer, 2009; Thoma, 2015).

Since these earlier reviews, there have been additional infertility prevalence studies that apply new analytic methods (Slama *et al.*, 2012; Mascarenhas *et al.*, 2012b; Thoma *et al.*, 2013; Polis *et al.*, 2017) or assess differences in instrumentation (Crawford *et al.*, 2015; Jacobson *et al.*, 2018). Although noting variation in the measures for ascertaining infertility, prior reviews have not assessed how these definitional differences may have accounted for this heterogeneity in findings. Additionally, prior studies have not examined and synthesized the specific methodological approaches used to assess infertility prevalence, which may provide important insights for developing a standardized approach. For example, some researchers propose the use of time-to-pregnancy (TTP) for ascertaining infertility (Joffe *et al.*, 2005; Bonde *et al.*, 2006), whereas many studies are based on self-reported or constructed binary measures of infertility (Stanford, 2013). Furthermore, the pooling of prevalence data is necessary for generating global and regional estimates based on the best available evidence. To address the need to account for variation in measurement and generate global and regional estimates, we conducted a systematic review and meta-analysis to: (i) identify and describe the approaches used to estimate infertility prevalence among representative populations, (ii) summarize and evaluate the published contemporary estimates of the prevalence of infertility by methodological and study characteristics and (iii) generate pooled estimates of 12-month infertility prevalence globally and by region, sex of respondent and methodological approach.

## Materials and methods

We conducted a systematic review and meta-analysis in accordance with the updated Preferred Reporting Items for Systematic Reviews and Meta-Analyses (PRISMA) (Page *et al.*, 2021) and the Meta-analysis of Observational Studies in Epidemiology (MOOSE) (Stroup *et al.*, 2000). The protocol of this study is registered at PROSPERO (Registration number: CRD42020211704) (Cox *et al.*, 2020). Prior to registering the protocol, it was reviewed by 11 advisory committee members with expertise in a range of fields including library science, systematic review, meta-analysis, infertility, reproductive medicine, epidemiology and population health.

### Search strategy

Three members of the research team including a public health librarian and two public health investigators developed an extensive search strategy. Two health informationists external to the project peer reviewed the search strategy for PubMed using the Peer Review of Electronic Search Strategies (PRESS) tool (McGowan *et al.*, 2016).

To identify peer-reviewed publications, we searched the following electronic databases: PubMed (US National Library of Medicine), Web of Science (Clarivate Analytics), CINAHL (EBSCO), Family & Society Studies Worldwide (EBSCO), Public Health (ProQuest) and Google Scholar (search strategies are presented in Supplementary Table SI) and hand searched reference lists of 21 relevant articles, mostly literature reviews (Supplementary Table SII: strategy 1). To identify for grey literature, we searched electronic databases (Public Health (ProQuest) and ProceedingsFirst (OCLC) databases), relevant websites (Supplementary Table SII: strategy 2), conference proceedings (Supplementary Table SII: strategy 3) and contacted experts in the field.

The search strategy included terms related to infertility (e.g. infertility, subfertility, infecundity, childlessness) and estimation (e.g. estimate, prevalence). We limited searches to those published between 1 January 1990 and 11 March 2021 with no language restrictions (non-English articles were translated to English). We selected 1990 as the lower bound cutoff because: (i) we aimed to determine the contemporary prevalence of infertility, (ii) an analysis of trends in infertility prevalence in 190 countries and territories found that levels of infertility in 2010 were similar to those in 1990 in most regions of the world (Mascarenhas *et al.*, 2012b), (iii) we wanted the range of time to be large enough to capture all relevant methodological approaches and (iv) Schmidt and Münster (1995) conducted a review of the prevalence of infertility and its measurement in ‘industrialized countries’ that spanned 1970 to 1992 and we wanted to extend and expand on this work.

### Identification of studies

#### Inclusion criteria

We defined infertility broadly as a disease of the male or female reproductive system based on a prolonged period of time in which pregnancy or live birth is not reached despite exposure to the risk of pregnancy; this includes sterility. General population and clinic-based studies were included if they met all of the following criteria: (i) designed to be a representative sample of a general population of women and/or men; (ii) reported estimates of the prevalence or cumulative incidence of infertility; (iii) collected data in or after 1990; (iv) specified, in their definition of infertility, a duration of least 6 months in which pregnancy was not reached, or defined infertility as a subjective evaluation of one’s difficulty conceiving or maintaining a pregnancy; (v) presented original research using primary or secondary data; and (vi) used one of the following study designs: cross-sectional, cohort, case-control (if the control group was a representative sample of the general population and the disease of interest (i.e. the case group) was not infertility), or randomized trial (if they reported an overall estimate for a representative sample of the general population at baseline, before any interventions were administered).

We considered studies as representative if they recruited, based on their study design, all eligible members of a population (i.e. through a census) or applied probability-based sampling. We defined clinic-based studies that applied consecutive sampling for 12 or more months as a census of the clinic population and thus considered these studies for inclusion. Furthermore, for clinic-based studies to meet the requirement of representing a general population, their samples had to have been drawn from a clinic that serves the general population (i.e. primary care clinic or an obstetrics and/or gynecology clinic) and was representative of the clinic population as a whole.

In our initial protocol, we specified that studies with representative samples of subgroup populations would be considered; however, subgroups were defined differently across several demographic characteristics (e.g. age, race, occupation) and health conditions (e.g. hypertension, cancer, polycystic ovary syndrome). Therefore, we amended these criteria to only include general populations given the large number of studies meeting these criteria and our desire to pool estimates through meta-analysis.

#### Exclusion criteria

We excluded studies if they met any of the following criteria: (i) reported cause-specific prevalence of infertility only, such as tubal factor infertility, or male-factor or female-factor infertility; (ii) estimated only the proportion seeking fertility treatment or receiving a diagnosis of infertility; (iii) did not use individuals as the unit of analysis (e.g. studies that estimated the percent of pregnancies with a time to pregnancy greater than 12 months whereby women could contribute more than one pregnancy to the analysis and the denominator reflected pregnancies, rather than an individual); (iv) measured childlessness without an intention to estimate infertility (e.g. a combined measure of voluntary and involuntary childlessness or a measure that did not distinguish reasons for involuntary childlessness); (v) included menopausal and/or surgically sterile individuals in their numerator, which would inflate the numerator with individuals who have completed their reproductive life span either naturally or surgically; (vi) did not define their measure of infertility; (vii) and/or reported results only as an abstract or unpublished data.

#### Screening process

We compiled all records identified from searches in Zotero, a reference management tool, and removed all duplicates. We then imported records into Rayyan, a web application (Ouzzani *et al.*, 2016), where two members of the research team independently screened the title and abstract of each record. Two members of the research team independently reviewed the full-text of studies that were marked as meeting or possibly meeting the inclusion criteria based on their title and abstract. We resolved disagreements at either stage through discussion to reach consensus.

In instances where duplicate publications of research results were identified, we linked the publications and selected a primary publication. We defined duplicate publications as publications that generated estimates of the prevalence of infertility using the same data source, definition of infertility, and approach to estimation.

### Data extraction

For each study, one researcher extracted the data using a form that we generated in Excel. A second researcher reviewed the data extraction for quality and accuracy on a random subset (n = 32). The form captured study and participant characteristics, study design, data collection details, infertility measure details (definition, numerator, denominator, exclusions, etc.), infertility prevalence estimates and methodological approach(es) (Supplementary Table SIII). For studies that included data collected both before and after 1990, we only extracted estimates calculated from data collected in or after 1990. In cases where studies presented multiple estimates after 1990, we extracted the most recent estimate. In instances where necessary information was unreported in a manuscript, we attempted to contact the corresponding author via email.

### Study descriptor variables

#### Methodological approaches

We classified methodological approaches to estimating infertility prevalence into six categories: (i) prospective time to pregnancy (TTP) design, (ii) retrospective TTP design, (iii) current duration design, (iv) self-reported infertility measure (direct), (v) constructed infertility measure (indirect) and (vi) undetermined. Categories 1–5 are described in more detail in Table I. The ‘undetermined’ category includes studies that measured infertility prevalence, but where the methodological approach was not clearly reported. We did not identify the approaches listed here a priori but, per our research aims, we identified them based on the results of our systematic literature search and analysis.

**Table I:** Description of five approaches to measuring infertility prevalence identified from the systematic review.

	Prospective time-to-pregnancy design^1^	Retrospective time-to-pregnancy design	Current duration design	Self-reported infertility measure (direct)	Constructed infertility measure (indirect)
**Description**	Participants are enrolled prior to period of unprotected intercourse (PUI) (incident cohort) or during a period of unprotected intercourse (prevalent cohort). Participants are followed until pregnancy, infertility treatment, or study conclusion (administrative censoring).	Participants are asked to recall the PUI or pregnancy attempt time prior to becoming pregnant (pregnancy-based approach). Alternatively, participants may be asked about a PUI and/or time-to-pregnancy (TTP) in a specified time regardless of outcome (historical prospective approach).	Participants are enrolled during a current PUI or pregnancy attempt. Current duration (CD) is calculated as the interval between when the PUI or pregnancy attempt began and date of interview. CD values are used to estimate a summary TTP distribution for the population using survival methods and under certain analytic assumptions.	Participants are queried directly about their ability to conceive either within a specified duration of time (e.g. 12 months) or based on their subjective evaluation.	Infertility status is determined based on the presence or absence of a pregnancy or live birth among couples exposed to conception for a defined period. Exposure to conception is inferred from survey questions and/or a reproductive calendar.
**Sample questions for querying respondents**	[For those planning to conceive] Are you pregnant (asked at specified intervals during follow-up)? [For those planning to conceive] pregnancy is ascertained by pregnancy testing over follow-up period	How long had/have you been trying to become pregnant? How many months did you have regular intercourse without contraception before you became pregnant?	[For those at risk of pregnancy at interview]: Series of questions on dates of last use of contraception, pregnancy, or birth. Current duration is calculated from start of at-risk interval to date of interview. [Among those at risk of pregnancy at interview] How long have you been trying to become pregnant? (number of months or years)	Have you ever experienced a period of at least 12 months where you were having unprotected intercourse (or attempting to become pregnant) but did not become pregnant? Have you and a partner ever had difficulty conceiving?	Constructed based on a series of questions or reproductive calendar on relationship status, birth history, contraceptive use, and, in some instances, sexual activity and desire to have another child.
**Common research objectives**	Assess the biologic capacity for reproduction (i.e. fecundity) Examine the relationship between risk factors on fecundity	Estimate fecundity or measure infertility prevalence Identify and/or examine risk factors, which need to be anchored around the start of the PUI or pregnancy attempt	Generate population-based estimates of infertility prevalence Identify and/or examine risk factors, which need to be anchored around the start of the PUI or pregnancy attempt	Estimate infertility prevalence Assess association between infertility and risk factors, outcomes and/or treatment seeking behavior	Generate population-based estimates of infertility prevalence with nationally representative demographic and reproductive health survey data
**Common applications for^2^:**					
Type of prevalence^3^	Period prevalence	Period or lifetime prevalence	Period prevalence	Period or lifetime prevalence	Period prevalence
Duration cut-off for infertility	12 months 24 months	12 months 24 months	12 months 24 months	12 months No duration (subjective measure)	12 months 60 months
Pregnancy intentions considered in numerator	Always considered	Sometimes considered	Sometimes considered	Sometimes considered	Commonly not considered
Denominator considered	Those attempting to conceive	Those ever at risk of pregnancy or attempting to conceive	Those at risk of pregnancy or attempting to conceive at time of interview	Ever and not at risk of pregnancy (e.g. all women of reproductive age)	Ever and not at risk of pregnancy (e.g. all women of reproductive age)

1 The prospective time-to-pregnancy design approach is considered the gold standard.

2 Common applications are summarized based on the studies included in the systematic review.

3 Period prevalence is defined as the proportion of individuals/couples with infertility at a given point or interval in time, which may be current or past depending on the study aims. Lifetime prevalence is defined as the proportion of individuals/couples who have ever experienced infertility in their life.

#### Definitional characteristics

##### Type of prevalence

We categorized type of prevalence as either period prevalence or lifetime prevalence. We defined period prevalence as the proportion of individuals/couples with infertility at a point or over an interval of time, which may be current or past (e.g. first birth) depending on the study aims. In some studies, the interval of time may refer to the period over which data were collected. In other studies, the interval of time may refer to infertility for a specified pregnancy or pregnant attempt (e.g. most recent) within a defined period, such as 5 years from the date of interview. Other studies measured period prevalence of infertility at a specific point of the respondents’ life such as their first year after marriage. In comparison, we defined lifetime prevalence as the proportion of individuals/couples who have ever experienced infertility at any point in their life. For example, when measuring lifetime infertility, researchers often asked respondents a question similar to, ‘have you ever tried to become pregnant for more than a year without succeeding?’

##### Numerator

We classified the numerator as (i) ‘duration-only’ (only considered the duration of time at risk of conception or attempting pregnancy), (ii) ‘duration and treatment’ (considered duration of time and whether treatment for infertility was sought and/or received) or (iii) ‘self-perceived infertility’ (with or without duration specified). We also categorized the numerator based on whether intentions were considered or not (i.e. trying to conceive).

##### Denominator

We categorized the denominator based on if and how studies accounted for risk of pregnancy. The three categories were, ‘individuals regardless of risk of pregnancy’, ‘individuals at risk of pregnancy regardless of intentions’ and ‘individuals attempting to conceive’.

#### Study population characteristics

##### Sample type

We dichotomously categorized sample type as ‘population-based sample’ or ‘clinic-based sample’ based on whether respondents were recruited from the entire target population (e.g. community-based sampling) or a clinic serving the general population of reproductive-aged individuals.

##### Sex of respondent

We categorized studies based on the sex of the respondents, as defined by each study, regardless of which partner experienced infertility. The categories included ‘female respondent’, ‘male respondent’ and ‘combined’, which included both male and female respondents or couple respondents. Studies that included both male and female respondents but reported estimates separately were categorized under both ‘female respondent’ and ‘male respondent’.

##### Income level

We classified study populations as either high-income or low- and middle-income based on their country-specific status at the time of analysis (2021) according to the World Bank classifications (World Bank, 2021).

##### Region

We categorized study populations into regions according to the six WHO regions: African Region, Region of the Americas, South-East Asia Region, European Region, Eastern Mediterranean Region, and Western Pacific Region (World Health Organization, 2022).

#### Risk of bias assessment

We assessed the risk of bias for each study using the Hoy *et al.* (2012) risk of bias tool that was slightly modified by the study team to better fit with infertility definitions assessed in this review (Supplementary Table SIV). The tool includes eight items assessing external and internal validity. For each item, we rated studies as either low or high risk. We classified studies that provided insufficient information to permit a judgment for a given item as high risk. We generated an overall summary score that is the sum of the eight individual items (1 point awarded for each item labeled as low risk). The overall summary score was divided into the following tertiles: (i) low risk of bias: 6–8 points, (ii) moderate risk of bias: 3–5 points and (iii) high risk of bias: 0–2 points. We assessed the potential for publication bias through funnel plots. We generated two funnel plots: one for studies reporting period prevalence and one for studies reporting lifetime prevalence. In our initial protocol, we indicated that we would use a different tool to assess risk of bias; however, we later selected the Hoy *et al.* tool because we felt it could be applied more systematically and objectively across team members.

### Data analysis

#### Descriptive analysis

We first identified and described the methodological approaches used to estimate the prevalence of infertility. We then reported the number of studies overall and by study descriptor variables. When analyzing the estimates of infertility prevalence, we focused on the definition adopted by the WHO (2018), which defines infertility as ‘a disease of the male or female reproductive system defined by the failure to achieve a pregnancy after 12 months or more of regular unprotected sexual intercourse’, which we hereafter refer to as ‘12-month infertility’. We examined and reported the number of studies and range of estimates of 12-month infertility overall and by study descriptor variables.

#### Meta-analysis and meta-regression

We applied meta-analysis to estimate period and lifetime prevalence of 12-month infertility overall and stratified by income level, region, respondent type, and methodological approach (Schmid *et al.*, 2021). We calculated standard errors (SE) of each study’s infertility prevalence estimate either: (i) by extracting the SE directly or calculating from the 95% CI ((upper interval–lower interval)/3.92), or when this information was not available, (ii) a SE was calculated based on the formula for obtaining a SE from a proportion (p) ([SQRT(p*(1−p))/N]). For studies that applied the formula, we further differentiated whether the study used simple random sampling or a complete census versus studies in which we approximated the SE from this formula because they applied complex sampling designs or used survival analysis. Sensitivity analyses were further conducted to examine the influence of studies that used an approximated SE compared to studies in which the SE could be estimated directly.

Estimates of 12-month infertility were transformed using the logit function [ln(p/(1−p))]. Corresponding SEs were logit transformed using the delta method [SQRT((1/(p*(1−p))^2^)*(SE^2^))]. To ensure independence across studies for the meta-analysis and assess the sensitivity of analytic choices on selection of estimates, we selected the maximum or the minimum lifetime and period infertility prevalence estimates for studies in which multiple estimates were presented (either in the same record or a duplicate record, which for the purposes of the meta-analysis was defined as an estimate that was generated from the same data source).

We generated pooled estimates for studies using the maximum value of the prevalence estimate for studies presenting multiple estimates, then repeated for studies using the minimum value in sensitivity analyses. We used random-effects meta-analysis models to generate pooled estimates, 95% CIs, *I*^2^ statistics (i.e. proportion of total variability in point estimates that can be attributed to heterogeneity), and forest plots. A decision to present pooled estimates was not solely based on *I*^2^ values, but was informed by consideration that higher *I*^2^ values are inevitable where sample sizes are large, and SEs are precise (Rücker *et al.*, 2008), which was consistent with the studies included in this review. We stratified pooled estimates by whether they were period or lifetime infertility and by income classification, region, methodological approach and respondent’s sex. Using a random-effects meta-analysis model, we derived funnel plots of the logit transformed prevalence estimates against their SEs.

Meta-regression using restricted maximum likelihood was applied to generate adjusted period and lifetime prevalence estimates of 12-month infertility after accounting for region, methodological approach, numerator included intentions, denominator categories and risk of bias score. We chose the covariates in the model based on variables of interest in estimation (i.e. region, methodological approach) or having a sufficient number of studies across each variable categorization and region. The exponentiated regression coefficient obtained from the meta-regression of the logit transformed infertility prevalence estimates provides odds ratios (ORs) for a given unit change in the covariate. Stata 16.1 was used to conduct meta-analyses and meta-regression (StataCorp, 2019).

#### Sensitivity analyses

In meta-analysis, we estimated pooled lifetime and period prevalence of infertility stratified by factors that influenced selection of estimates into the meta-analysis (i.e. maximum versus minimum values for linked studies or SE calculation assumptions) as described above or based on study characteristics. Study characteristics consisted of restricting meta-analysis to estimates obtained from the highest quality studies (risk of bias > 6) or studies from the general population only.

## Results

**Figure 1.**
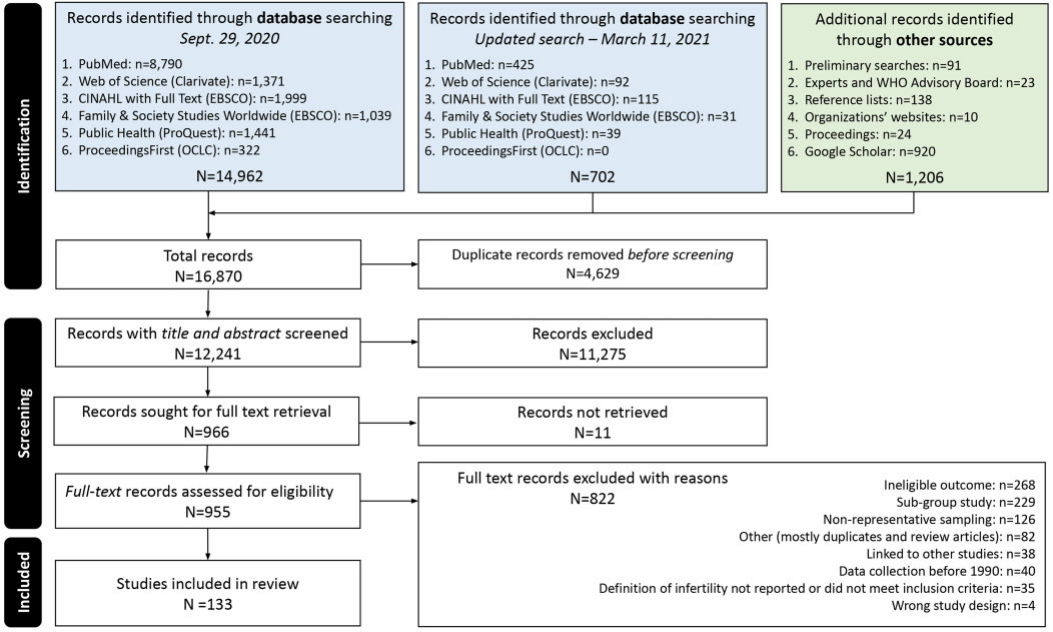
**Flowchart of the identification of studies via databases and other methods**.

A PRISMA flow diagram illustrating the literature search, article selection and final included studies is shown in Fig. 1 and follows the updated PRISMA guideline by Page *et al.* (2021).

### Description of studies

Our search yielded 16 870 records, and after removing duplicates, resulted in 12 241 unique records. Two reviewers independently screened the title and abstract of each unique record and identified 966 records to be reviewed in full text. Eleven of the 966 records could not be located (Supplementary File S1). Of the remaining 955 records, 171 records met our inclusion criteria. We excluded duplicate publications not selected as the primary publication and list them in Supplementary Table SV (n = 38). Thus, a total of 133 studies were included in our systematic review (Supplementary File S2). Supplementary Table SVI provides an overview of the study characteristics and infertility prevalence estimates for each study. The vast majority of studies were cross-sectional in design (n = 115). This count includes cohort studies that used cross-sectional data to generate their estimate of infertility prevalence. Thirteen studies used a cohort study design and five used a case-control study design for which we only extracted data for the control groups, who were representative samples of a general population.

The analytic sample in 85 studies included individuals of reproductive age, which was defined differently across studies but often confined to individuals aged 15–49 or 20–44 years old. Nineteen studies provided a lower age limit without an upper age limit and/or an age limit that extended beyond reproductive age. Fifteen studies limited the sample to a single age or a smaller age range that captures women in different stages of their reproductive life (e.g. 20–34 years, 30–49 years). For seven studies, all measuring lifetime prevalence of infertility, the sample included individuals beyond reproductive age. Ten studies did not report the age range of respondents in their analytic sample. Three studies reported estimates for two different age groupings and are thus represented in multiple tallies.

There were 66 studies which restricted their sample to individuals who were married or in a union, while 53 studies did not restrict their sample by relationship status, and a few studies reported both estimates separately. Eleven studies did not report the relationship status of respondents. Some studies explicitly or implicitly excluded individuals not engaged in heterosexual intercourse. Only one study reported the percent of respondents self-identifying as gay, lesbian or bisexual.

The most common definition applied to the estimates of infertility prevalence was a 12-month definition of infertility in 101 studies. There were 30 studies which applied a 24-month definition of infertility while 14 studies applied a demographic 60-month definition of infertility, and 29 studies applied definitions with durations other than 12-, 24- or 60 months (e.g. 6 months, 36 months) or with no duration at all (self-perceived infertility). Among the studies that defined infertility by duration, all studies measured infertility in months with no studies measuring infertility in menstrual cycles. Many studies reported estimates for multiple definitions of infertility. There were 60 studies which reported total infertility prevalence estimates (i.e. primary and secondary infertility combined in a single estimate) while 34 studies reported total, primary and secondary infertility prevalence estimates, whereas the remaining 39 studies reported some other combination of total, primary and/or secondary infertility prevalence estimates.

### Assessment of the risk of bias

**Figure 2.**
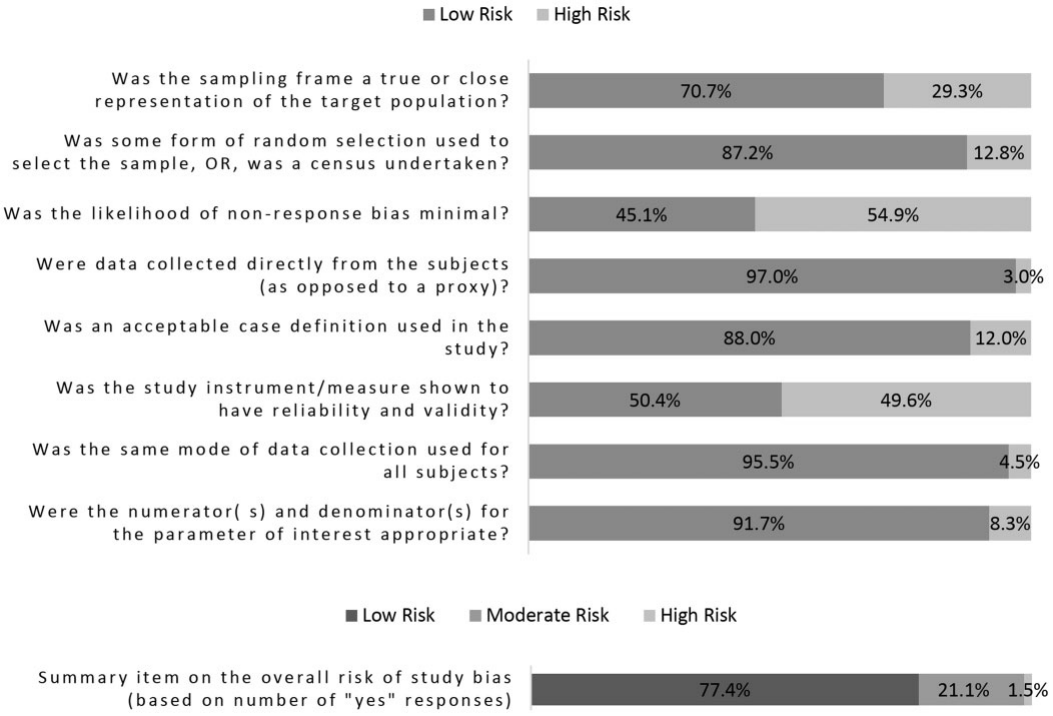
**Risk of bias of included studies**.

We found that the overall risk of bias was low for 77.4% of studies, moderate for 21.1% of studies, and high for 1.5% (Fig. 2; risk of bias ratings for individual studies can be found in Supplementary Table SVII). For five out of eight individual items assessed, we rated at least 87.2% of studies as low risk. Only one item, the item measuring the likelihood of non-response, had more than half (54.9%) of studies rated as high risk. This item required studies to have a reported response rate of 75% or higher to be rated as low risk. (We rated studies not reporting a response rate in the manuscript as high risk.) Another item had 49.6% of studies rated as high risk and measured whether the study instrument or measure was shown to be reliable and valid. There were several reasons why a study would be rated as high risk for this particular item, which are described in the assessment tool in Supplementary Table SIV. For example, we rated studies that used the reproductive calendar to indirectly classify women as infertile as high risk as well as studies that used proxy measures for unprotected sex.

Another item with nearly 29.3% of studies rated as high risk measured whether the sampling frame was a true or close representation of the target population. We excluded non-representative studies from the systematic review, thus the studies rated as high risk for this item represent clinic-based studies and/or studies that restricted their sample to pregnant women. While the sampling frames in these studies were representative of the clinic and/or pregnant population, they were at a higher risk of bias than the general population-based studies because their samples excluded those not seeking biomedical care or those with unresolved infertility respectively and thus were not necessarily representative of the general population, which was the target population for this review.

The funnel plots were symmetrical for studies reporting estimates of lifetime and period prevalence of infertility (Supplementary Figs S1 and S2).

### Methodological approaches for estimating infertility prevalence

The review comprised studies that fell into all six methodological categories: (i) prospective TTP design, (ii) retrospective TTP design, (iii) current duration design, (iv) self-reported infertility measure (direct), (v) constructed infertility measure (indirect) and (vi) undetermined. For 13 studies, we could not determine the approach based on the information provided in the manuscript and were unable to contact the author for clarification either due to an invalid email address or no response. Table I provides a description of each approach and common applications based on the studies included in our review and Table II reports the number of studies and range of estimates by methodological approach.

**Table II:** Number of studies and range of 12-month period and lifetime infertility prevalence estimates by study descriptors.

Study characteristics	Number of studies^1^	Number and range of 12-month total infertility prevalence estimates (%)^2^			
	**All studies (studies with 12-month estimates)**	**Number of period estimates^3^**	**Period prevalence**	**Number of lifetime estimates^3^**	**Lifetime prevalence**
**Total**	**133 (84)**	**69**	**1.6–34.0**	**65**	**3.3–39.7**
**Methodological approaches**					
Prospective TTP design	3 (3)	3	13.6–28.0	–	–
Retrospective TTP design	34 (24)	25	5.0–32.0	15	3.3–35.3
Current duration design	6 (5)	10	9.4–34.0	–	–
Self-reported infertility measure	61 (39)	16	4.0–18.0	45	4.2–39.7
Constructed infertility measure	23 (8)	12	6.0–17.0	–	–
Undetermined	13 (6)	3	1.6–13.3	5	10.1–20.9
**Definitional characteristics**					
**Numerator (duration only)**					
Intentions included^4^	65 (46)	22	7.0–32.0	42	4.2–39.7
Intentions not considered	61 (37)	44	1.6–34.0	14	3.3–35.3
**Numerator (duration and/or receipt of care included)**					
Intentions included^4^	8 (7)	2	12.0–12.3	8	11.0–26.0
Intentions not considered	2 (2)	1	18.0	1	35.0
**Numerator (subjective evaluation with or without duration)**					
Intentions included^4^	10 (1)	–	–	3	11.4–16.4
Intentions not considered or unknown	10 (1)	1	7.74	–	–
**Denominator**					
All regardless of risk of pregnancy	74 (41)	19	1.6–17.0	35	3.3–35.0
Ever at risk of pregnancy^5^	37 (26)	30	4.2–34.0	13	8.2–35.3
Attempting to conceive^4^	40 (30)	20	9.4–32.0	17	5.8–39.7
**Study population characteristics**					
**Sample type**					
General population-based	118 (71)	47	1.6–34.0	65	3.3–39.7
Clinic-based	15 (13)	22	5.0–28.0	–	–
**Sex of respondent**					
Female	109 (72)	54	1.6–34.0	56	3.3–39.7
Male	10 (10)	5	7.0–15.3	9	8.2–21.8
Combined^6^	18 (9)	10	4.2–28.0	–	–
Not reported	1 (–)	–	–	–	–
**Income Level** ^7^					
High-income countries	70 (55)	43	5.0–34.0	52	4.2–35.3
Low- and middle-income countries	65 (29)	26	1.6–32.0	13	3.3–39.7
**Region** ^8^					
Africa Region	24 (8)	6	9.5–32.0	4	9.3–15.8
Eastern Mediterranean Region	15 (6)	3	5.2–15.2	4	3.3–21.2
European Region	47 (37)	32	5.0–34.0	25	9.0–31.8
South-East Asia Region	12 (–)	–	–	–	–
Region of the Americas	24 (15)	16	4.0–15.7	15	4.2–35.3
Western Pacific Region	29 (19)	12	1.6–28.0	17	8.2–39.7

1 Some studies reported multiple prevalence estimates by applying different definitional or study population characteristics. In these instances, studies were included in more than one tally.

2 12-month estimates of resolved and unresolved infertility. Outlier 12-month infertility estimate is not reported in Table II. Outliers were determined based on their magnitude and justification in the respective studies regarding their ability to capture infertility. Outlier infertility estimates are documented in Supplementary Data S10.

3 Some studies reported multiple prevalence estimates by applying different definitional or study population characteristics. In these instances, multiple estimates from a single study may be included in the same tally.

4 Includes individuals wanting a child and/or trying to conceive.

5 Includes any individual ever at risk of a pregnancy. May include studies that used marital status as a proxy for being at risk of pregnancy.

6 Includes studies that reported estimates for male and female respondents or couple respondents.

7 Defined based on World Bank classifications at the time of the systematic review (The World Bank. Countries and economies. 2021; Available from: https://data.worldbank.org/country).

8 Defined based on World Health Organization regional groupings (World Health Organization. 2022; Available from: https://www.who.int/about/who-we-are/regional-offices).

TTP, time to pregnancy.

Of the five main approaches used to estimate prevalence among studies in our review, the self-reported infertility measure was applied most often followed by the retrospective TTP design (Table II). The prospective TTP design was the least used approach in our review with only three studies identified. We observed the same trends when only considering studies that reported at least one prevalence estimate of 12-month infertility. Six studies reported multiple estimates generated by different approaches and are thus included in the count for multiple approaches in Table II. Three studies combined two approaches to generate a single estimate. In these instances, we categorized these studies based on the primary approach used for generating the prevalence estimate.

Across studies, period prevalence was measured using all approaches, whereas lifetime prevalence was mainly measured using the self-reported infertility measure approach. The 12-month definition was the most common definition applied across all approaches except for the constructed infertility measure for which a 5-year definition was more commonly applied.

Use of a retrospective TTP design was more common in HICs, particularly Europe, whereas use of the constructed infertility measure approach was more common in low- and middle-income countries (LMICs). The self-reported infertility measure approach was widely applied in studies conducted in both HIC and LMIC. China was the only country in our review that used a prospective TTP design approach, where participants were recruited from premarital and preconception clinics widely available in China but uncommon in most other countries. The self-reported infertility measure approach was the most common approach applied in studies conducted in the regions of Africa, the Americas and Western Pacific. The self-reported infertility measure approach was also commonly applied in Europe; however, in this region, the retrospective TTP design approach was the most widely applied approach. The constructed infertility measure was the most common approach used in studies conducted in South-East Asia and Eastern Mediterranean and was also commonly applied in studies in Africa and the Americas.

The majority of 12-month infertility prevalence estimates were based on self-reported infertility measures and retrospective TTP designs (Table II and Supplementary Fig. S3). Across approaches, duration-based methods (prospective TTP, retrospective TTP and current duration designs) showed larger period estimates and ranges of 12-month infertility (5.0–34.0%) compared with self-reported and constructed measures (4.0–18.0%). Lifetime estimates of 12-month infertility were available only for retrospective TTP (3.3–35.3%) and self-reported measures (4.2–39.7%) and were comparable.

### Definitional characteristics

#### Type of prevalence

There were 84 studies that reported a period prevalence and 58 that reported a lifetime prevalence, with some reporting both. In some studies, lifetime prevalence was estimated among those who were no longer of reproductive age whereas other studies included individuals who were still of reproductive age and may not have completed childbearing. There were 134 estimates of 12-month infertility extracted from 84 studies, of which 69 were period prevalence and 65 were lifetime prevalence (Table II). Period and lifetime estimate ranges of 12-month infertility were both wide and comparable to one another.

#### Numerator

The majority of studies overall and those reporting 12-month infertility prevalence estimates used a numerator defined by duration only (Table II). Among duration-only estimates, about half included intentions (mainly defined as those trying to conceive) in the numerator and the other half did not. Some studies reported both. Among studies reporting 12-month estimates, more than half of the studies considered intentions. A much smaller number of studies incorporated duration and receipt of care in the numerator. Twenty studies used a numerator defined by subjective evaluation (i.e. perceived infertility) with or without a specified duration. Only two studies defined their numerator by subjective evaluation and/or a duration of 12 months. The range of period and lifetime estimates of infertility among studies that defined the numerator by duration only did not vary considerably by whether the numerator considered intentions (7.0–32.0%, 4.2–39.7%, respectively) or not (1.6–34.0% with one outlier removed, 3.3–35.3%, respectively).

#### Denominator

More than half of the studies included individuals regardless of their risk of pregnancy in the denominator. The remaining studies were split in how they defined their denominator between those ever at risk of pregnancy and those attempting to conceive (Table II). Some studies provided multiple estimates in their publication using different denominators. Among studies reporting 12-month estimates, the distribution among the three categories was more evenly divided than for all studies. Period infertility estimate ranges were lower when the denominator included individuals regardless of risk (1.6–17.0%) compared to individuals ever at risk (4.2–34.0%) or individuals attempting to conceive (9.4–32.0%). Lifetime infertility prevalence estimates were relatively similar across denominator categorizations.

### Study population characteristics

#### Sample type

Most studies drew their sample from the general population (n = 118), whereas only 15 studies drew their sample from a clinic population. Among the clinic-based studies, 12 restricted their sample to pregnant women (measuring TTP). Studies reporting 12-month estimates of infertility were also more likely to be of the general population. Period infertility estimate ranges were similar for general population studies compared to clinic-based studies. There were no lifetime estimates of 12-month infertility available for clinic-based studies.

#### Sex of respondents

An overwhelming majority of studies included estimates based on female respondents (n = 109), while only 10 studies included estimates based on male respondents. (Five studies that included separate estimates for female and male respondents were counted in both tallies.) Eighteen studies included estimates that combined responses from both male and female respondents or from couples. One study did not report the sex of respondents. The range for period and lifetime estimates of 12-month infertility was smaller and lower for male respondents compared to female respondents.

#### Income level

The proportion of study populations from HIC and LMIC was similar at 51.9% and 48.1%, respectively, whereas 65.5% of the 12-month infertility prevalence estimates were from HIC (Table II). Only two studies, both led by Mascarenhas (2012a,b), presented estimates from both HIC and LMIC. Overall, the range of period and lifetime estimates of 12-month infertility were similar within and across HIC and LMIC (Supplementary Fig. S4).

#### Region

Europe was the region represented in the greatest proportion of studies (35.3% of the total number of studies). Eastern Mediterranean and South-East Asia were the least represented regions in our review with only 15 (11.3% of the total number of studies) and 12 studies (9.0% of the total number of studies), respectively (Table II). The regions reporting the greatest number of 12-month estimates were Europe, the Americas and Western Pacific. Very few 12-month estimates were available for Africa and Eastern Mediterranean regions and no 12-month estimates were available for South-East Asia. Overall, period infertility prevalence estimate ranges were largest for the African (9.5–32.0%), European (5.0–34.0%) and Western Pacific regions (1.6–28.0%) compared to the Americas (4.0–15.7%) and Eastern Mediterranean regions (5.2–15.2%). Lifetime infertility prevalence estimate ranges were largest for the Americas (4.2–35.3%), European (9.0–31.8%) and Western Pacific (8.2–39.7%) regions and smallest for the African region (9.3–15.8%) (Supplementary Fig. S5 and Table II).

### Pooled 12-month infertility prevalence estimates

**Figure 3.**
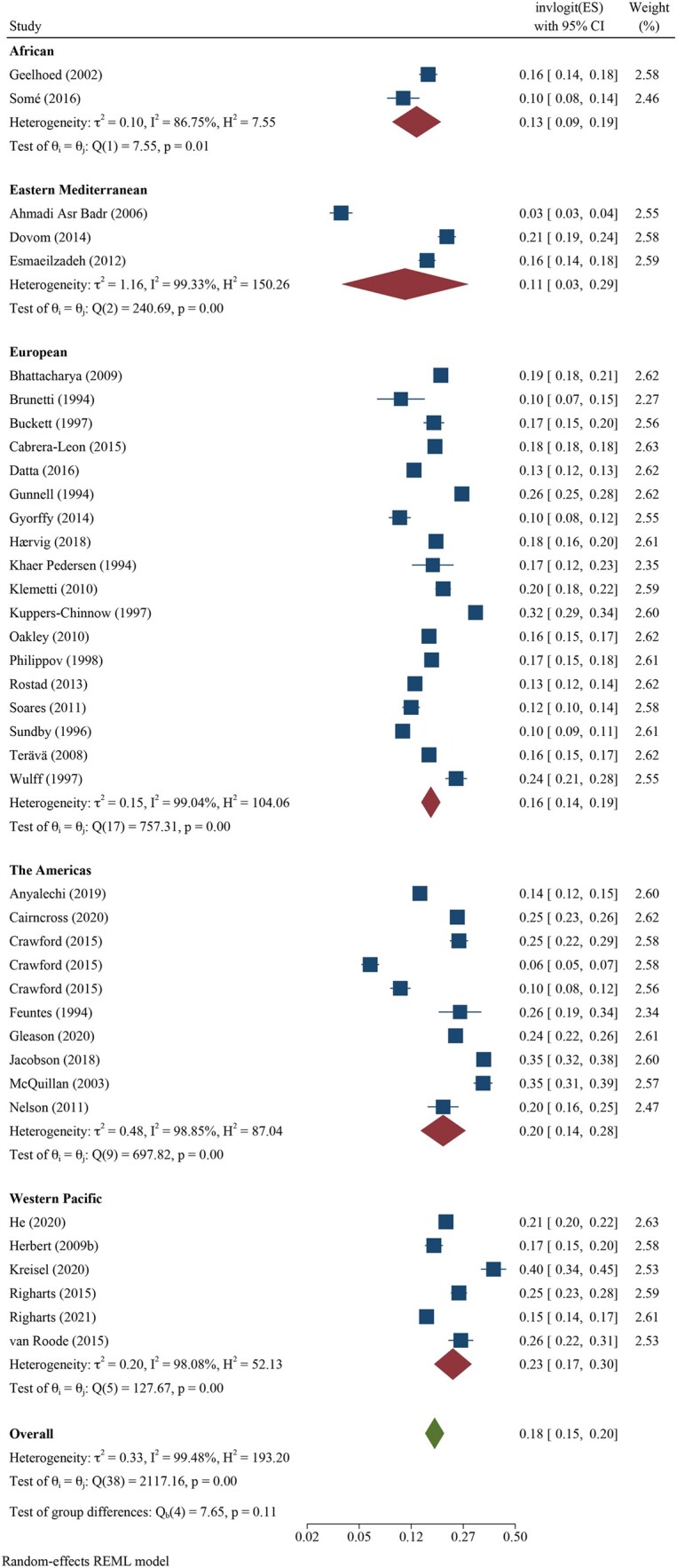
**Forest plot of pooled lifetime prevalence of 12-month infertility by region**.

**Figure 4.**
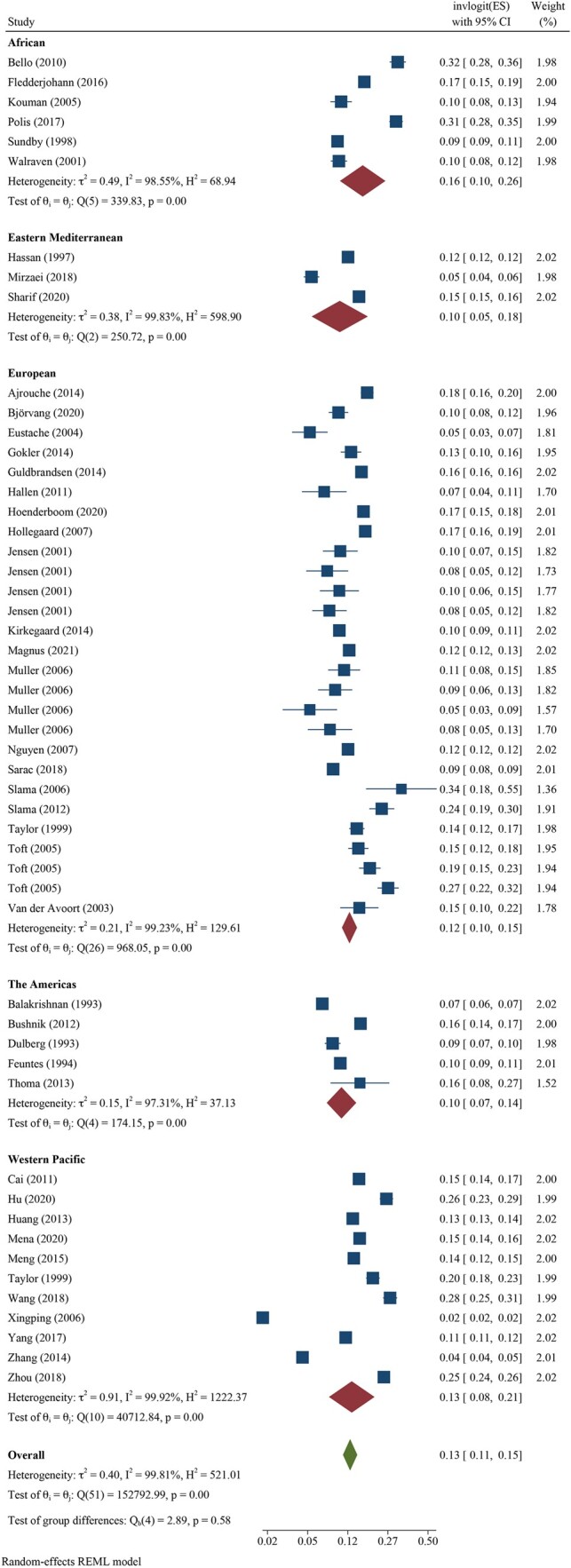
**Forest plot of pooled period prevalence of 12-month infertility by region**.

We pooled all 12-month infertility prevalence estimates using meta-analysis and stratified by whether the measure was estimating lifetime (n = 39 independent estimates from 37 studies) or period prevalence (n = 52 independent estimates from 43 studies). Overall, pooled lifetime and period prevalence estimates were 17.5% (95% CI: 15.0, 20.3, *I*^2^ = 99.5%) and 12.6% (95% CI: 10.7, 14.6, *I*^2^ = 99.8%), respectively (Figs 3 and 4, respectively). Visual inspection of the forest plots showed a wide range of point estimates and a high degree of non-overlapping 95% CIs across individual studies. For primary 12-month infertility, pooled lifetime and period prevalence was 9.6% (95% CI: 6.3, 14.3, n = 12, *I*^2^ = 99.9%) and 9.0% (95% CI: 6.6, 12.2, n = 33, *I*^2^ = 99.9%), respectively (Supplementary Fig. S6). For secondary 12-month infertility, pooled lifetime and period prevalence was 6.5% (95% CI: 3.9, 10.7, n = 10, *I*^2^ = 99.4%) and 4.9% (95%: 2.7, 8.8, n = 17, *I*^2^ = 99.9%), respectively (Supplementary Fig. S7). For studies that presented more than one 12-month infertility prevalence estimate, sensitivity analyses showed minimal variation in lifetime and period estimates when selecting the minimum value over the maximum value for infertility prevalence (Supplementary Table SVIII). Similarly, restricting analyses to only higher quality studies with a bias score of 7 or 8 (n = 28 for lifetime, n = 16 for period), general population studies (n = 39 for lifetime, n = 30 for period) or studies in which the SEs could be directly ascertained from the publication (n = 28 for lifetime, n = 39 for period), rather than approximated, also showed little difference in overall infertility prevalence compared with the main findings (Supplementary Table SVIII).

#### Pooled infertility prevalence estimates stratified by population and study design characteristics

Stratification by study population characteristics showed estimates that were similar by income classification and region (i.e. CIs overlapped). When stratified by income classifications, we found pooled lifetime infertility prevalence to be 17.8% (95% CI: 15.3, 20.7, n = 30, *I*^2^ = 99.3%) for HIC and 16.5% (95% CI: 10.4, 25.0, n = 9, *I*^2^ = 99.2%) for LMIC. Pooled period infertility prevalence was slightly lower than lifetime estimates at 12.6% (95% CI: 10.8, 14.7, n = 31, *I*^2^ = 99.2%) for HIC and 12.6% (95% CI: 9.2, 16.9, n = 21, *I*^2^ = 99.9%) for LMIC (data not shown).

Regional differences in pooled lifetime infertility prevalence showed some variation in magnitude, yet all CIs overlapped (Fig. 3). Western Pacific region had the highest prevalence of lifetime infertility (23.2%, 95% CI: 17.4, 30.2, n = 6, *I*^2^ = 98.1%), followed by the regions of the Americas (20.0%, 95% CI: 13.9, 27.9%, n = 10, *I*^2^ = 98.9%), Europe (16.5%, 95% CI: 14.1, 19.2, n = 18, *I*^2^ = 99.0%) and Africa (13.1%, 95% CI: 8.6, 19.4, n = 2, *I*^2^ = 86.8%), and the lowest magnitude was found in Eastern Mediterranean (10.7%, 95% CI: 3.4, 29.0, n = 3, *I*^2^ = 99.3%). Similarly, the magnitude of period infertility prevalence estimates varied by region, but all CIs overlapped (Fig. 4). The highest pooled estimate of period infertility prevalence was in the African region (16.4%, 95% CI: 10.0, 25.7, n = 6, *I*^2^ = 98.6%) followed by Western Pacific (13.0%, 95% CI: 7.8, 20.8, n = 11, *I*^2^ = 99.9%), European (12.4%, 95% CI: 10.5, 14.6, n = 27, *I*^2^ = 99.2%), the Americas (10.4%, 95% CI: 7.4, 14.3, n = 5, *I*^2^ = 97.3%) and Eastern Mediterranean regions (10.0%, 95% CI: 5.2, 18.2, n = 3, *I*^2^ = 99.8%). The number of studies for lifetime and period estimates varied across regions, contributing to the variation in estimates. Notably, no studies conducted in the South-East Asian region provided overall 12-month infertility prevalence estimates.

Stratification by study design characteristics included respondent population (female, male, combined) and methodological approach used for estimation. The majority of lifetime and period prevalence estimates were based on female respondents (n = 37, n = 41, respectively) compared to male respondents (n = 12, n = 2, respectively) or combined sex (n = 0, n = 9, respectively). Pooled lifetime infertility prevalence estimates were higher when study respondents were female (17.5%, 95% CI: 14.9, 20.5, n = 37, *I*^2^ = 99.5%) compared with male (12.4%, 95% CI: 10.5, 14.6, n = 12, *I*^2^ = 95.7%). This pattern was also observed for pooled period estimates, but was based on only two studies that used male respondents. Pooled period infertility prevalence estimates were 12.6% (95% CI: 10.6, 15.0, n = 41, *I*^2^ = 99.8%) based on female respondents, 8.7% (95% CI: 5.1, 14.4, n = 2, *I*^2^ = 34.7%) based on male respondents, and 12.6% (95% CI: 8.2, 18.8, n = 9, *I*^2^ = 99.7%) based on combined (male, female, couple) respondents (data not shown).

**Figure 5.**
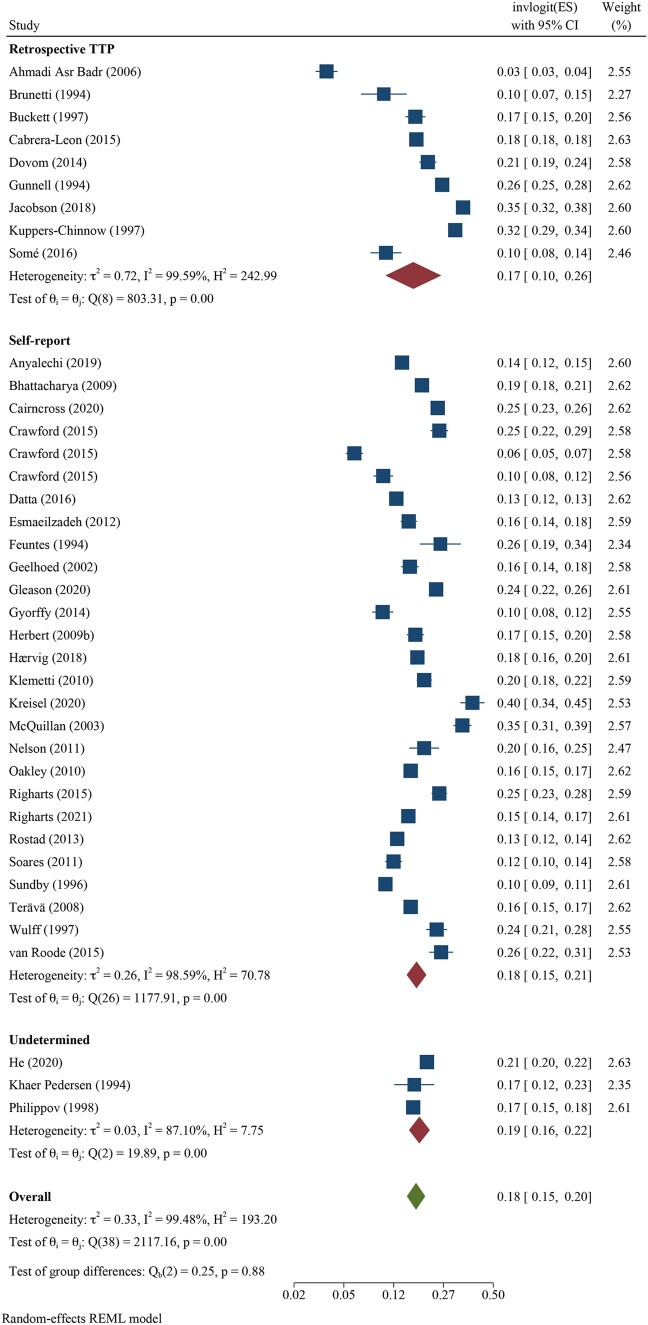
**Forest plot of pooled lifetime prevalence of 12-month infertility by methodological approach**.

**Figure 6.**
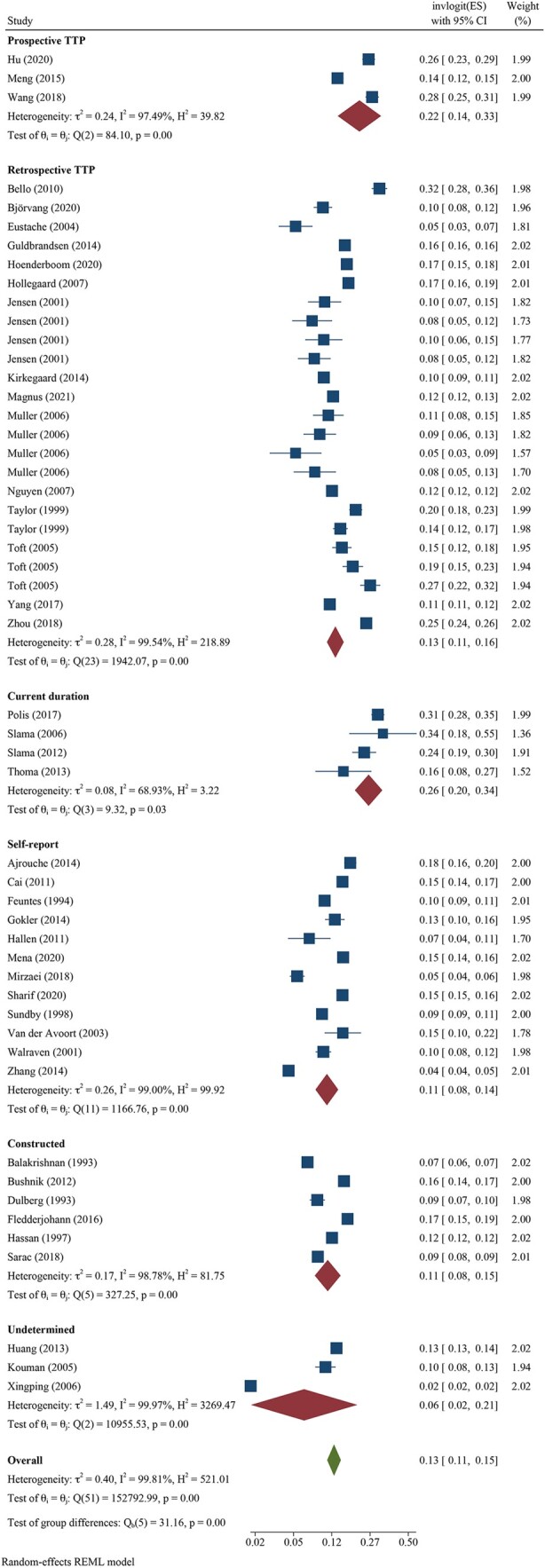
**Forest plot of pooled period prevalence of 12-month infertility by methodological approach**.

Methodological approach varied based on reporting of lifetime or period prevalence estimates. We found minimal differences in lifetime estimates across the three methodological approaches that were used with estimates of 16.7% (95% CI: 10.3, 26.0, n = 9, *I*^2^ = 99.6%), 17.6% (95% CI: 15.0, 20.7, n = 27, *I*^2^ = 98.6%) and 18.5% (95% CI: 15.6, 21.8, n = 3, *I*^2^ = 87.1%) for retrospective TTP, self-reported infertility, and undetermined approaches, respectively (Fig. 5). In contrast, period estimates were highest when using prospective TTP (21.8%, 95% CI: 13.7, 32.9, n = 3, *I*^2^ = 97.5%) and current duration approaches (26.2%, 95% CI: 19.9, 33.6, n = 4, *I*^2^ = 68.9%) followed by a retrospective TTP approach (12.9%, 95% CI: 10.7, 15.6, n = 24, *I*^2^ = 99.5%) (Fig. 6). Self-reported and constructed approaches were similar with pooled period infertility prevalences of 10.6% (95% CI: 8.1, 13.8, n = 12, *I*^2^ = 99.0%) and 10.9% (95% CI: 8.0, 14.6, n = 6), respectively. The lowest infertility prevalence was found for the three studies in which the approach could not be determined (6.2%, 95% CI: 1.6, 20.8, n = 3, *I*^2^ = 99.9%).

#### Meta-regression results by period and lifetime

Meta-regression results yielded patterns similar to unadjusted pooled lifetime infertility prevalence (Table III). Although CIs overlapped, the magnitude of the ORs showed generally higher lifetime infertility prevalence in the Americas (OR: 1.33, 95% CI: 0.81, 2.18) and Western Pacific regions (OR: 1.34, 95% CI: 0.72, 2.49) and lower magnitude in the African (OR: 0.60, 95% CI: 0.24, 1.26) and Eastern Mediterranean (OR: 0.64, 95% CI: 0.31, 1.30) regions relative to the European region after adjustment for definitional characteristics and bias scores. This corresponded to pooled adjusted lifetime infertility prevalence estimates of 20.5%, 23.4%, 13.0%, and 10.8% for each respective region compared to the European region (16.8%). In the same model, differences by methodological approach were minimal with adjusted lifetime infertility prevalence estimates of 17.8% for retrospective TTP, 18.5% for undetermined, and 18.2% for the self-reported direct measures. This corresponded to OR associations of 0.85 (95% CI: 0.49, 1.47) for retrospective TTP and 1.02 (95% CI: 0.49, 2.12) for undetermined relative to self-reported direct measures. The overall *I*^2^ percentage was slightly reduced with adjustment (98.6%).

**Table III:** Pooled lifetime and period infertility prevalence estimates and multivariable odds ratios associations for region and methodological approach, adjusting for definitional factors and risk of bias.

Study covariates	Infertility prevalence, % (95% CI)	**Multivariable model** ^1^ **odds ratio (95% CI)**
**Lifetime prevalence (n = 39 estimates)** ^2^		
**Region** ^3^		
African	13.0 (4.6, 21.3)	0.60 (0.24, 1.26)
Eastern Mediterranean	10.8 (5.0, 16.6)	0.64 (0.31, 1.30)
European	16.8 (13.4, 20.2)	Ref
The Americas	20.5 (15.2, 25.8)	1.33 (0.81, 2.18)
Western Pacific	23.4 (15.9, 31.0)	1.34 (0.72, 2.49)
**Methodological approach**		
Prospective TTP	–	–
Retrospective TTP	17.8 (12.8, 22.9)	0.85 (0.49, 1.47)
Current duration	–	–
Self-reported direct measure	18.2 (15.2, 21.1)	Ref
** **Constructed measure	–	–
** **Undetermined	18.5 (9.3, 27.5)	1.02 (0.49, 2.12)
**Period prevalence (n = 52 estimates)** ^2^		
**Region** ^3^		
African	18.1 (11.7, 24.5)	1.95 (1.02, 3.72)
Eastern Mediterranean	10.1 (4.4, 15.7)	1.11 (0.51, 2.42)
European	12.6 (10.2, 15.0)	Ref
The Americas	11.2 (6.2, 16.2)	0.88 (0.41, 1.87)
Western Pacific	14.2 (10.1, 18.2)	1.32 (0.77, 2.27)
**Methodological approach**		
Prospective TTP	21.8 (11.1, 32.6)	1.42 (0.53, 3.84)
Retrospective TTP	13.1 (10.5, 15.7)	1.10 (0.65, 1.85)
Current duration	26.0 (14.6, 37.3)	2.43 (1.17, 5.05)
Self-reported direct measure	10.9 (7.8, 14.1)	Ref
Constructed measure	11.1 (6.7, 15.5)	1.31 (0.69, 2.47)
Undetermined	6.2 (2.5, 9.9)	0.48 (0.22, 1.07)

(–) indicates that 12-month estimates were not found for respective categories.

1 Models were adjusted for region, methodological approach (prospective TTP, retrospective TTP, current duration, self-reported binary measure, constructed binary measure, undetermined), numerator included intentions, denominator categories (all regardless of risk, ever at risk, attempting to become pregnant) and risk of bias score (0–8).

2 Lifetime prevalence (*I*^2^ = 98.6%), period prevalence (*I*^2^ = 99.2).

3 Overall lifetime and period 12-month estimates were not found for studies conducted in South East Asian regions.

TTP, time to pregnancy.

Similarly, meta-regression results for period infertility prevalence were consistent with unadjusted results (Table III). Relative to studies from the European region, infertility prevalence estimates from the African region were associated with the largest magnitude of association (OR: 1.95, 95% CI: 1.02, 3.72) followed by the Western Pacific (OR: 1.32, 95% CI: 0.77, 2.27) and Eastern Mediterranean regions (OR: 1.11, 95% CI: 0.51, 2.42). The Americas (OR: 0.88, 95% CI: 0.41, 1.87) had a lower magnitude of association relative to estimates from the European region, although CIs overlapped for all regions with the exception of the African region. These OR associations were consistent with adjusted period prevalence estimates, which showed the highest prevalence in the African region (18.1%) followed by the Western Pacific (14.2%), European (12.6%), the Americas (11.2%), and Eastern Mediterranean regions (10.1). The magnitude of association between period infertility estimates and methodological approach showed higher ORs for prospective TTP (OR: 1.42, 95% CI: 0.53, 3.84), retrospective TTP (OR: 1.10, 95% CI: 0.65, 1.85), current duration approach (OR: 2.43, 95% CI: 1.17, 5.05) and constructed measure (OR: 1.31, 95% CI: 0.69, 2.47) relative to the self-reported direct measure; however, differences were only statistically significant for the current duration approach and CIs overlapped when comparing across other approaches. Period infertility estimates based on undetermined methodology were lower (OR: 0.48, 95% CI: 0.22, 1.07) relative to the self-reported direct measure, but this was not statistically significantly different.

## Discussion

Global estimates of infertility are needed to guide planning and coordination of infertility prevention, diagnosis and treatment efforts, particularly estimates that are consistent with clinical definitions that can guide services. This systematic review included 133 studies that reported period and/or lifetime estimates of the prevalence of infertility in a representative sample of a general population. It expands on previous studies by providing more contemporary global and regional summary estimates of infertility and explicitly examines the variability across study characteristics. Additionally, this study identifies gaps in the availability of studies for certain regions of the globe and for particular study populations, such as male respondents. The studies included in the review represent both HIC and LMIC as well as all regions of the world. Studies from the European region were the most well represented in our review (35.3% of included studies) whereas studies from the Eastern Mediterranean (11.3%) and South-East Asian (9.0%) regions were the least represented. When we restricted the studies to those reporting an estimate of 12-month infertility (N = 84), we found that the most and least represented regions stayed the same; however, proportionally, the gap between the most and least represented regions was greater (44.0% of included studies represented the European region compared to 7.1% from Eastern Mediterranean and 0% from South-East Asian regions).

We identified five main approaches used to estimate infertility prevalence among the studies included in our review: (i) prospective TTP design, (ii) retrospective TTP design, (iii) current duration design, (iv) self-reported infertility measure and (v) constructed infertility measure. The approach for some studies could not be determined. While ranges in period and lifetime estimates of 12-month infertility were wide, they were often comparable across methodological approaches, definitional characteristics and study population characteristics.

In meta-analyses, overall lifetime prevalence of 12-month infertility had a higher magnitude than period prevalence, as was expected, and based on *I*^2^ percentages and visual inspection of forest plots, we found a high level of heterogeneity across studies. When stratified by HIC and LMIC, lifetime and period infertility prevalence estimates were similar across income classifications. The Western Pacific region had the highest prevalence of lifetime infertility, whereas the African region had the highest prevalence of period infertility, although CIs overlapped across regions. In contrast, using a demographic infertility measure (5-year exposure period) and different regional groupings than our study, Mascarenhas *et al.* (2012b) found the highest primary infertility prevalence rate in the North Africa/Middle East region and the highest secondary infertility prevalence rate in the Central/Eastern Europe and Central Asia region. Interestingly, Mascarenhas *et al.* (2012b) reported greatest data availability from the South Asian and sub-Saharan African regions, which were similar to the two regions with the least data availability for 12-month estimates in our meta-analysis (South-East Asian and African regions). For the studies that provided primary and/or secondary infertility prevalence estimates, we found a higher global prevalence of primary compared to secondary infertility. This may be due to a larger proportion of estimates from HIC or LMIC (i.e. China and Iran) with lower fertility in which secondary infertility may not be recognized due to earlier completed childbearing.

Comparing across methodological approaches, lifetime prevalence estimates were similar for the three approaches compared (retrospective TTP design, self-reported infertility measure and undetermined), whereas period prevalence estimates were slightly higher for prospective TTP and current duration design approaches compared with the other approaches. Similarly, Thoma *et al.* (2013) applied the same definition for estimating infertility prevalence, but found an almost two-fold higher prevalence of infertility based on estimated TTP from a current duration approach (15.5%) compared with a constructed approach (7.0%). In our study, adjusted infertility prevalence estimates for region and methodological approach ascertained through meta-regression showed magnitudes of infertility prevalence and patterns similar to unadjusted estimates. Although the number of studies with male respondents was limited, pooled lifetime and period infertility prevalence estimates reported by male respondents were lower than for female respondents.

We rated nearly all studies in our review as low or moderate risk of bias with only 1.5% of studies rated as high risk. The greatest area of risk among studies included in our review was response bias with more than 50% of studies reporting a response rate below 75% or failing to report a response rate. Additionally, we conducted sensitivity analyses to assess the robustness of findings after excluding high-risk studies with minimal impact on the overall estimates. To assess the potential for publication bias, we generated funnel plots, which were found to be symmetrical, suggesting lower potential for publication bias; however, caution should be used in interpreting these results given limitations on the interpretation of funnel plots for this purpose (Sterne and Egger, 2001).

Regarding the reporting of infertility prevalence estimates, most studies reported either a total infertility prevalence estimate or total, primary and secondary infertility prevalence estimates. Some studies suggested that estimates of primary infertility should be used for making comparisons across time and settings due to the potential biases that may arise by including subsequent pregnancies (Basso *et al.*, 2000; Olsen, 2003). However, some studies have reported significantly higher rates of secondary infertility than primary infertility in certain contexts and regions such as Africa, where infection-related infertility from postpartum infections or unsafe abortions is higher (Larsen, 2000; Sharma *et al.*, 2009) and thus excluding secondary infertility from infertility prevalence estimates would result in an underestimation or distortion of the total burden of infertility in the population. We report global estimates of primary and secondary 12-month infertility but were unable to explore regional differences in primary and secondary 12-month infertility prevalence due to a lack of sufficient number of studies across regions.

Period and lifetime measures of infertility prevalence provide different information about population burden and need for services, but both play an important role in our understanding of infertility prevalence. Estimates of current period infertility prevalence help countries identify service needs and target resources, whereas estimates of lifetime infertility prevalence provide an understanding of the burden of infertility over people’s lifetime. Surprisingly, we found that the range of 12-month infertility prevalence estimates was broad and did not vary substantially by period or lifetime prevalence. This may be due to the majority of studies capturing lifetime prevalence from reproductive-aged individuals who may not have completed childbearing. The wide range of estimates held even after accounting for definitional or study population characteristics. This finding was consistent with a prior systematic review by Gurunath *et al.* (2011), which found considerable heterogeneity of infertility prevalence estimates by definition, denominator and study population.

Patterns of infertility prevalence estimates by income level and region were also similar within and across approaches. For example, the range of infertility prevalence estimates for HIC and LMIC were similar, which is consistent with another (non-systematic) review examining 12 and 24-month infertility prevalence estimates by Boivin *et al.* (2007). In our study, regional comparisons showed that infertility estimate ranges were widest and had the largest estimates for the African, European and Western Pacific regions compared with the Americas and Eastern Mediterranean regions.

When examining differences by study design characteristics, we found some important differences in the application of the five methodological approaches identified in our review. Fewer studies conducted in LMIC used duration-based approaches (prospective TTP, retrospective TTP and current duration designs) compared to those in HIC, which may be limiting as duration-based approaches allow for more flexibility in how estimates are calculated and reported. In contrast, we found that more studies conducted in LMIC relative to HIC used the constructed infertility measure approach, which is an indirect approach for ascertaining infertility among respondents (i.e. relies on assumptions regarding the participant’s risk of pregnancy). This is likely a reflection of a lack of direct information collected on infertility in population-based surveys conducted in LMIC. Regional differences in the number and type of methodological approach used and how the approaches are operationalized may impact our ability to ascertain true difference in infertility prevalence between regions. However, adjustment for known sources of heterogeneity had minimal impact when comparing between unadjusted and adjusted regional estimates.

We also found definitional variation across studies, which is consistent with the findings of other systematic reviews (Schmidt and Münster, 1995; Gurunath *et al.*, 2011). In our review, the majority of studies reported an estimate of the prevalence of 12-month infertility suggesting that population-level estimates can be generated globally using this definition. When exploring definitional characteristics, we found that nearly all studies defined their numerator by duration only (with or without consideration of intentions) while only a limited number of studies defined their numerator by duration along with receipt of fertility care. Depending on the objectives of the research, including receipt of fertility care in the definition of the numerator may be important, especially as access to and uptake of fertility care continue to increase worldwide. Additionally, restricting the numerator to those intending to conceive (slightly more than 50% of studies in this review) may generate a more useful estimate for predicting service needs (White *et al.*, 2006; Greil *et al.*, 2016), whereas not restricting to those with intentions may be more useful for examining risk factors associated with infertility (Slama *et al.*, 2014). More than half of the studies did not restrict their denominator to those at risk of or attempting pregnancy. Including individuals regardless of risk of pregnancy in the denominator provides a broad understanding of the proportion experiencing infertility within a reproductive population; however, it could induce variation across populations that differ on their at-risk status (i.e. non-contraceptive use, non-sexually active).

Only a few studies applied a consistent definition and methodological approach across different regions. The majority of these studies were from LMIC and used data from Demographic and Health Survey (DHS) to infer exposure to conception from survey questions and/or a reproductive calendar (Rutstein and Shah, 2004; Mascarenhas *et al.*, 2012a,b). Furthermore, these studies relied on the demographic definition of infertility (i.e. an inability of a non-contraceptive using, sexually active woman to have a live birth, generally after 5 or 7 years of exposure). This definition does not meet the 12-month definition of infertility used by WHO (2018) and may be too long a duration for understanding clinical needs. Furthermore, women who experienced infertility prior to this time frame may not be captured, because they are no longer sexually active due to divorce or abandonment resulting from infertility.

One other study used DHS data and applied a current duration approach for estimating 12-month infertility across different countries and regions (Keiding *et al.*, 2021a). They found that the derivation of current duration values from the date of cohabitation was not feasible in certain country settings. Other studies have applied different methods for deriving the current duration measure within a single country setting based on calendar information on contraceptive discontinuation, pregnancy loss or birth or direct questions on time spent trying to become pregnant, suggesting other approaches for deriving current duration values may need to be explored (Slama *et al.*, 2012; Thoma *et al.*, 2013; Polis *et al.*, 2017, 2021; Keiding *et al.*, 2021b). Finally, one other study by Taylor *et al.* (1999) used a retrospective TTP design, applying standard questions, to compare infertility prevalence estimates in Australia (Western Pacific Region) and the UK (European Region) and found a higher prevalence of infertility in Melbourne, Australia (20.2%) compared with Manchester, UK (14.5%) among comparable study populations. These estimates were within the range of other studies found in each respective region and were consistent with our regional comparisons showing higher infertility in Western Pacific compared to European regions.

Other studies have compared estimates across different numerators or denominators within the same study population and found clear differences in infertility prevalence (Larsen, 2005; Crawford et al., 2015; Jacobson et al., 2018). However, in our review, we did not find much differentiation after accounting for numerator or denominator differences across studies, suggesting that other factors, such as survey questions or eligibility criteria (e.g. exclusions based on relationship status, use of infertility treatment, intentions of pregnancy, timing and frequency of sexual intercourse), may be masking true differences and need to be considered when making comparisons across studies. For example, Crawford *et al.* (2015) examined lifetime infertility prevalence from three states in the USA and noted that variations in population age distribution and in the wording and sequential flow of questions precluded conclusions about variation in infertility between states. Furthermore, the impact of these factors on infertility prevalence estimates will likely vary by country due to cultural and contextual factors. For example, the impact of restricting a sample to married couples on estimates of infertility prevalence would be minimal in contexts where premarital sexual intercourse is uncommon and substantial where it is common. A study conducted in Jamaica, for example, justified not restricting their sample to married women due to the variety of conjugal unions in that context (Priestley, 2012); however, this would not be justified in all settings.

Taken together, we examined a number of sources of heterogeneity in our systematic review and meta-analysis using a variety of techniques, including reporting of *I*^2^ values, visual inspection of forest plots, subgroup analyses of population characteristics (e.g. region, gender of respondents) or methodological approach, and meta-regression to adjust for definitional or methodological differences across studies. Although *I*^2^ values were high and the range of point estimates of individual studies was wide with a high degree of non-overlapping 95% CIs, suggesting heterogeneity across estimates, results were fairly consistent across subgroup and sensitivity analyses. Given that *I*^2^ values will be large when comparing highly precise estimates (Rücker *et al.*, 2008), which was the case for the majority of population-based studies examined in our study, we did not solely rely on this statistic in our decision to conduct meta-analyses. This decision was further informed by consultations with both the WHO technical committee and our advisory committee for this systematic review. Nevertheless, we acknowledge that other sources of heterogeneity could not be examined in this study, such as accounting for differences in the age distribution of the population, which would require the presentation of age-stratified or age-adjusted estimates of infertility. Additionally, studies should distinguish between the age in which infertility occurred from the age of the respondent at the time of study, for which the latter could take place years after the occurrence of infertility. Furthermore, there are potential unmeasured factors inherent to the study of infertility prevalence, such as stigma or cultural biases in reporting infertility, which may lead to underestimation of infertility in some populations. Future studies of infertility prevalence could examine these potential sources of heterogeneity to quantitatively assess the impact of these differences on estimates.

### Limitations

There are some limitations to the methodological choices we made that could impact our findings. First, we chose to focus on biological-based infertility, which excludes those experiencing ‘social infertility’ or ‘conditional childlessness’, which is described as childlessness due to legal, regulatory or social constraints (Davis and Khosla, 2020). This decision was, in part, due to a lack of consensus on other types of infertility as well as a lack of research. This exclusion eliminates some individuals from our estimates who might desire to use fertility treatments.

Second, we considered clinic-based studies for inclusion in our systematic review if they used a census or probability-based sample and drew their sample from a primary care, obstetrics and/or gynecology clinic that served the general population; however, the validity of using clinic-based samples to represent a sample of the ‘general population’ varies based on context. This may be a reasonable assumption for countries with universal healthcare systems, where individuals are likely to be seen for well-person visits and obstetrical care. For example, some studies in our review sampled from prenatal or birthing facilities and noted that these facilities would cover almost all pregnancies for that geographic area (Toft *et al.*, 2005; Raatikainen *et al.*, 2010; Kirkegaard *et al.*, 2014). In contexts where use of preventative and/or biomedical services is limited or under-used by certain groups of individuals (e.g. rural populations, lower-income individuals, smokers and those without health insurance coverage) (Azagba *et al.*, 2013; Seo *et al.*, 2019; Park and Kim, 2021; Shibre *et al.*, 2021), the use of a clinic-based sample to represent the general population may be biased. Our sensitivity analyses found that this limitation may be minimal for the studies included in our review given there was little difference between estimates that included clinic-based studies compared to estimates restricted to the general population studies only.

Third, we excluded non-representative and subgroup studies because of our interest in identifying the current prevalence of infertility for global populations. However, these criteria excluded some large, reputable prospective cohort studies that apply the methodological approaches identified in this review (Buck *et al.*, 2002, 2012; Yland *et al.*, 2022).

Fourth, we observed significant variation in the level of detail provided in publications, making it difficult in some cases to discern definitional characteristics (e.g. period versus lifetime prevalence) and/or approaches used in estimating infertility prevalence. Some studies explicitly stated the numerator and denominator used in their estimate, which was extremely helpful in understanding how the researchers’ reported definition of infertility was operationalized in a given study. Furthermore, providing the verbatim survey question(s) used to generate the estimate of infertility prevalence was beneficial, and in some cases, essential, for identifying the methodological approach used. Only about one third of studies included in our review reported the survey question(s) in their manuscript. Comprehensively reporting the methods and results is essential for understanding, interpreting and comparing estimates. More research is needed to understand the broad variability in infertility prevalence estimates that is maintained even after differences in definition and approach are taken into account.

Fifth, we generated pooled estimates of period and lifetime prevalence of 12-month infertility that differed by methodological approach, definitional characteristics and study population characteristics. We conducted meta-regression to adjust for these factors; however, the adjusted estimates were similar to the unadjusted estimates, suggesting that additional factors, real or otherwise, may account for some of the differences across studies. For example, studies varied in their inclusion criteria by age, which is a strong determinant of infertility and affects the length of time one may be exposed to risk of infertility; however, the age at infertility versus the age at interview could not be differentiated for many of the studies included in this review. While pooling data might not be ideal under such circumstances it was necessary to generate prevalence estimates at regional and global levels. Data included spanned three decades (1990–2021) and, therefore, potential changes in prevalence over time period may have occurred; however, due to heterogeneity in the timing of when 12-month estimates were ascertained, we were unable to provide trend analysis. For example, the date of data collection of all studies included in this review occurred in or after 1990; but the recall period may reflect an earlier time period given respondents were reporting on past experiences. Also trend analysis would require using smaller time periods, reduce the data available for inclusion at each time period, and limit our broad view of the methodological approaches in contemporary use. However, trend analysis could be attempted in future or within studies that could apply the same measurement of infertility over time.

Sixth, in the stratified analysis, we generated the estimates for some strata from only a few studies. For example, the pooled lifetime estimate of infertility for the African region included only two studies (Geelhoed *et al.*, 2002; Somé *et al.*, 2016), which may explain the conflicting and unexpected result of lifetime prevalence being lower than period prevalence in the African region.

Lastly, we did not use the GRADE approach (Guyatt *et al.*, 2011) to rate the quality of evidence and strength of recommendations since there was a lack of formal guidance for use of this approach in reviews of prevalence (Migliavaca *et al.*, 2020). However, specific domains of GRADE criteria were assessed in our study, including risk of bias, precision of estimates, publication bias and inconsistency. The first three of these domains showed minimal impact on the overall findings and a high level of precision, suggesting further confidence in the quality of evidence presented. On the other hand, we observed a wide range of point estimates and non-overlapping 95% CIs across studies applying similar definitions, and a high degree of unexplained heterogeneity, resulting in some reduction of our confidence in the quality of evidence based on the criterion of inconsistency. Our review provides an opportunity for further analysis, which could contribute to the development of formal guidance for applying the GRADE approach to reviews of prevalence data.

## Conclusion and recommendations

**Figure 7.**
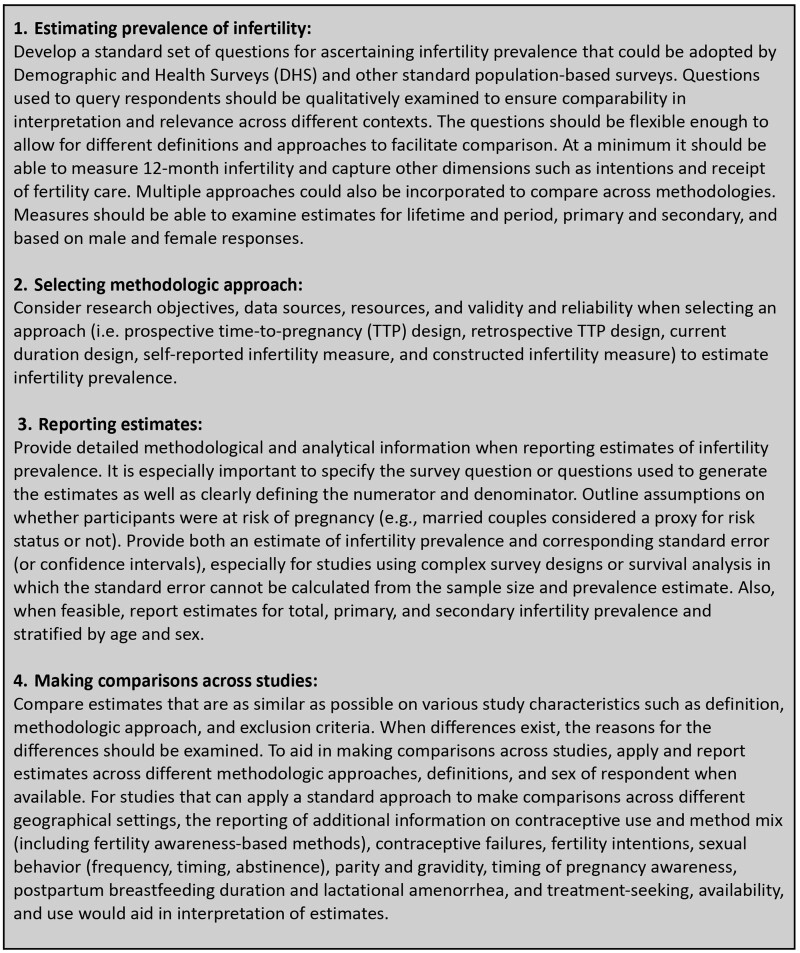
**Key recommendations based on results of the systematic review and meta-analysis**.

Accounting for aspects and sources of heterogeneity, we generated pooled estimates of period and lifetime prevalence of 12-month infertility globally and by region, methodological approach and respondent type with the best available data. Additionally, our study enables further insight on components that could be addressed in future infertility prevalence research. As such, we propose several recommendations to improve infertility prevalence estimates and our ability to compare these estimates across settings and time (Fig. 7). Valid and reliable estimates of infertility are needed to understand its burden and to facilitate advocacy, and provision and monitoring of prevention efforts and fertility care services. Findings from this systematic review and meta-analysis show high rates of infertility globally and regionally. The findings also reveal limitations in data used to measure infertility prevalence and highlights the urgent need for more systematic and comprehensive collection of data for measuring infertility prevalence at global, regional and country levels.

## Supplementary Material

hoac051_Supplementary_Tables1_8

hoac051_Supplementary_Figures

hoac051_Supplementary_Files1_2

## Data Availability

A spreadsheet with data inputs is available from the corresponding author. Stata code for key analytic steps will also be shared on reasonable request to the corresponding author.
