# Supplementary material for: Infertility prevalence and the methods of estimation from 1990 to 2021: a systematic review and meta-analysis
Source: Hum Reprod Open. 2022 Nov 12;2022(4):hoac051. doi: 10.1093/hropen/hoac051 (PMC9725182; doi:10.1093/hropen/hoac051)
Supplement: hoac051_Supplementary_Tables1_8 [file hoac051_supplementary_tables1_8.docx]

#

#

# Supplementary Table SI: Search strategies

**PubMed**

|  | Query | Notes | Results Sept. 29, 2020 | Results March 11, 2021 |
| --- | --- | --- | --- | --- |
| #8 | ((("Childlessness"[Title/Abstract] OR "Delayed conception"[Title/Abstract] OR "Time-to-conception"[Title/Abstract] OR "Fecundability"[Title/Abstract] OR "Fecundity"[Title/Abstract] OR "Inability to conceive"[Title/Abstract] OR "Infecundity"[Title/Abstract] OR "Infertility"[Title/Abstract] OR "Infertility, Female/epidemiology"[MeSH] OR "Infertility, Male/epidemiology"[MeSH] OR "Infertility/epidemiology"[MeSH] OR "Sterility"[Title/Abstract] OR "Subfecundity"[Title/Abstract] OR "Subfertility"[Title/Abstract] OR "Time-to-pregnancy"[MeSH] OR "Time-to-pregnancy"[Title/Abstract]) AND ("Estimate"[Title/Abstract] OR "Estimated"[Title/Abstract] OR "Estimates"[Title/Abstract] OR "Estimating"[Title/Abstract] OR "Estimation"[Title/Abstract] OR "Incidence"[Title/Abstract] OR "Prevalence"[MeSH] OR "Prevalence"[Title/Abstract] OR "Prevalent"[Title/Abstract])) NOT ("Case study"[Title/Abstract] OR "Case studies"[Title/Abstract] OR "Case reports"[Publication Type])) NOT ("Animals"[MeSH] NOT "Humans"[MeSH]) | **Filters applied:** from 1990 - 2020  **Updated search:** Filters applied - from 2020/09/29 - 2021/03/11 | 8,790 | 425 |
| #7 | ((("Childlessness"[Title/Abstract] OR "Delayed conception"[Title/Abstract] OR "Time-to-conception"[Title/Abstract] OR "Fecundability"[Title/Abstract] OR "Fecundity"[Title/Abstract] OR "Inability to conceive"[Title/Abstract] OR "Infecundity"[Title/Abstract] OR "Infertility"[Title/Abstract] OR "Infertility, Female/epidemiology"[MeSH] OR "Infertility, Male/epidemiology"[MeSH] OR "Infertility/epidemiology"[MeSH] OR "Sterility"[Title/Abstract] OR "Subfecundity"[Title/Abstract] OR "Subfertility"[Title/Abstract] OR "Time-to-pregnancy"[MeSH] OR "Time-to-pregnancy"[Title/Abstract]) AND ("Estimate"[Title/Abstract] OR "Estimated"[Title/Abstract] OR "Estimates"[Title/Abstract] OR "Estimating"[Title/Abstract] OR "Estimation"[Title/Abstract] OR "Incidence"[Title/Abstract] OR "Prevalence"[MeSH] OR "Prevalence"[Title/Abstract] OR "Prevalent"[Title/Abstract])) NOT ("Case study"[Title/Abstract] OR "Case studies"[Title/Abstract] OR "Case reports"[Publication Type])) NOT ("Animals"[MeSH] NOT "Humans"[MeSH]) | #5 NOT #6 | 9,950 | 10,248 |
| #6 | "Animals"[MeSH] NOT "Humans"[MeSH] | Animal studies to be excluded | 4,739,369 | 4,798,531 |
| #5 | (("Childlessness"[Title/Abstract] OR "Delayed conception"[Title/Abstract] OR "Time-to-conception"[Title/Abstract] OR "Fecundability"[Title/Abstract] OR "Fecundity"[Title/Abstract] OR "Inability to conceive"[Title/Abstract] OR "Infecundity"[Title/Abstract] OR "Infertility"[Title/Abstract] OR "Infertility, Female/epidemiology"[MeSH] OR "Infertility, Male/epidemiology"[MeSH] OR "Infertility/epidemiology"[MeSH] OR "Sterility"[Title/Abstract] OR "Subfecundity"[Title/Abstract] OR "Subfertility"[Title/Abstract] OR "Time-to-pregnancy"[MeSH] OR "Time-to-pregnancy"[Title/Abstract]) AND ("Estimate"[Title/Abstract] OR "Estimated"[Title/Abstract] OR "Estimates"[Title/Abstract] OR "Estimating"[Title/Abstract] OR "Estimation"[Title/Abstract] OR "Incidence"[Title/Abstract] OR "Prevalence"[MeSH] OR "Prevalence"[Title/Abstract] OR "Prevalent"[Title/Abstract])) NOT ("Case study"[Title/Abstract] OR "Case studies"[Title/Abstract] OR "Case reports"[Publication Type]) | #3 NOT #4 | 11,636 | 11,997 |
| #4 | "Case study"[Title/Abstract] OR "Case studies"[Title/Abstract] OR "Case reports"[Publication Type] | Types of studies to be excluded | 2,201,814 | 2,242,806 |
| #3 | ("Childlessness"[Title/Abstract] OR "Delayed conception"[Title/Abstract] OR "Time-to-conception"[Title/Abstract] OR "Fecundability"[Title/Abstract] OR "Fecundity"[Title/Abstract] OR "Inability to conceive"[Title/Abstract] OR "Infecundity"[Title/Abstract] OR "Infertility"[Title/Abstract] OR "Infertility, Female/epidemiology"[MeSH] OR "Infertility, Male/epidemiology"[MeSH] OR "Infertility/epidemiology"[MeSH] OR "Sterility"[Title/Abstract] OR "Subfecundity"[Title/Abstract] OR "Subfertility"[Title/Abstract] OR "Time-to-pregnancy"[MeSH] OR "Time-to-pregnancy"[Title/Abstract]) AND ("Estimate"[Title/Abstract] OR "Estimated"[Title/Abstract] OR "Estimates"[Title/Abstract] OR "Estimating"[Title/Abstract] OR "Estimation"[Title/Abstract] OR "Incidence"[Title/Abstract] OR "Prevalence"[MeSH] OR "Prevalence"[Title/Abstract] OR "Prevalent"[Title/Abstract]) | #1 AND #2 | 11,971 | 12,346 |
| #2 | "Estimate"[Title/Abstract] OR "Estimated"[Title/Abstract] OR "Estimates"[Title/Abstract] OR "Estimating"[Title/Abstract] OR "Estimation"[Title/Abstract] OR "Incidence"[Title/Abstract] OR "Prevalence"[MeSH] OR "Prevalence"[Title/Abstract] OR "Prevalent"[Title/Abstract] | Concept #2: Estimate/prevalence | 2,450,356 | 2,537,998 |
| #1 | "Childlessness"[Title/Abstract] OR "Delayed conception"[Title/Abstract] OR "Time-to-conception"[Title/Abstract] OR "Fecundability"[Title/Abstract] OR "Fecundity"[Title/Abstract] OR "Inability to conceive"[Title/Abstract] OR "Infecundity"[Title/Abstract] OR "Infertility"[Title/Abstract] OR "Infertility, Female/epidemiology"[MeSH] OR "Infertility, Male/epidemiology"[MeSH] OR "Infertility/epidemiology"[MeSH] OR "Sterility"[Title/Abstract] OR "Subfecundity"[Title/Abstract] OR "Subfertility"[Title/Abstract] OR "Time-to-pregnancy"[MeSH] OR "Time-to-pregnancy"[Title/Abstract] | Concept #1: Infertility | 87,175 | 89,892 |

**Web of Science**

|  | Query | Notes | Results Sept. 29, 2020 | Results March 11, 2021 |
| --- | --- | --- | --- | --- |
| #5 | #3 NOT #4 | All searches were limited in advance to the following:  - DOCUMENT TYPES: (Article OR Abstract of Published Item OR Book OR Book Chapter OR Meeting Abstract OR Meeting Summary OR Proceedings Paper OR Reprint OR Review)  - Indexes=SSCI, CPCI-S, CPCI-SSH  - Timespan=1990-2021 | 1,371 | 92 |
| #4 | SU=(Fisheries OR "Veterinary Sciences" OR Zoology) | Animal studies to be excluded | 116,642 | 4,793 |
| #3 | #1 AND #2 | Combining Concept #1 and Concept #2 | 1,510 | 93 |
| #2 | TI=(Epidemiolog* OR Estimat* OR Incidence OR Prevalen*) OR AB=(Epidemiolog* OR Estimat* OR Incidence OR Prevalen*) | Concept #2: Estimate/prevalence | 1,159,351 | 68,269 |
| #1 | TI=(Childlessness OR "Delayed conception" OR Fecundability OR Fecundity OR "Inability to conceive" OR Infecundity OR Infertility OR Sterility OR Subfecundity OR Subfertility OR "Time to conception" OR "Time to pregnancy") OR AB=(Childlessness OR "Delayed conception" OR Fecundability OR Fecundity OR "Inability to conceive" OR Infecundity OR Infertility OR Sterility OR Subfecundity OR Subfertility OR "Time to conception" OR "Time to pregnancy") | Concept #1: Infertility | 10,848 | 682 |

**CINAHL with full text (EBSCO)**

|  | Query | Notes | Results Sept. 29, 2020 | Results March 11, 2021 |
| --- | --- | --- | --- | --- |
| #6 | Filtered to 1990-2020, Source Types: Academic Journals, Dissertations, Books | Updated search: Filtered to Sept. 2020-March 2021, Source Types | 1,999 | 115 |
| #5 | #3 NOT #4 |  | 2,065 | 2,184 |
| #4 | (MH "Animals" NOT MH "Human") | Filter used from <https://blocks.bmi-online.nl/catalog/16>   Creators: Bramer WM, Fowler S, Ket JCF, Otten RHJ, and Riphagen II | 80,779 | 82,435 |
| #3 | #1 AND #2 |  | 2,081 | 2,200 |
| #2 | (TI Epidemiolog*) OR (AB Epidemiolog*) OR (TI Estimat*) OR (AB Estimat*) OR (TI Incidence) OR (AB Incidence) OR (TI Prevalen*) OR (AB Prevalen*) | Concept #2: Estimate/prevalence | 575,918 | 602,162 |
| #1 | (MM "Childlessness/EP") OR (TI Childlessness) OR (AB Childlessness) OR (TI "Delayed conception") OR (AB "Delayed conception") OR (TI Fecundability) OR (AB Fecundability) OR (TI Fecundity) OR (AB Fecundity) OR (TI "Inability to conceive") OR (AB "Inability to conceive") OR (TI Infecundity) OR (AB Infecundity) OR (TI Infertility) OR (AB Infertility) OR (TI Sterility) OR (AB Sterility) OR (TI Subfecundity) OR (AB Subfecundity) OR (TI Subfertility) OR (AB Subfertility) OR (TI "Time to conception") OR (AB "Time to conception") OR (TI "Time to pregnancy") OR (AB "Time to pregnancy") | Concept #1: Infertility | 12,110 | 12,734 |

**Family & Society Studies Worldwide (EBSCO)**

|  | Query | Notes | Results Sept. 29, 2020 | Results March 11, 2021 |
| --- | --- | --- | --- | --- |
| #4 | Filtered to 1990-2020, Source Types: Academic Journals, Books, Reports, Conference papers, Working papers, Reviews | Updated search: Filtered to Sept. 2020-March 2021 | 1,039 | 31 |
| #3 | #1 AND #2 |  | 1,426 | 1,452 |
| #2 | (TI Epidemiolog*) OR (AB Epidemiolog*) OR (TI Estimat*) OR (AB Estimat*) OR (TI Incidence) OR (AB Incidence) OR (TI Prevalen*) | Concept #2: Estimate/prevalence | 123,407 | 125,753 |
| #1 | (TI Childlessness) OR (AB Childlessness) OR (TI "Delayed conception") OR (AB "Delayed conception") OR (TI Fecundability) OR (AB Fecundability) OR (TI Fecundity) OR (AB Fecundity) OR (TI "Inability to conceive") OR (AB "Inability to conceive") OR (TI Infecundity) OR (AB Infecundity) OR (TI Infertility) OR (AB Infertility) OR (TI Sterility) OR (AB Sterility) OR (TI Subfecundity) OR (AB Subfecundity) OR (TI Subfertility) OR (AB Subfertility) OR (TI "Time to conception") OR (AB "Time to conception") OR (TI "Time to pregnancy") OR (AB "Time to pregnancy") | Concept #1: Infertility | 8,890 | 8,986 |

**Public Health (ProQuest)**

|  | Query | Notes | Results Sept. 29, 2020 | Results March 11, 2021 |
| --- | --- | --- | --- | --- |
| #4 | Filtered to 1990-2020, Source type - Scholarly Journals, Dissertations & Theses, Reports | Updated search: Filtered to Sept. 2020 - March 2021 | 1,441 | 39 |
| #3 | #1 AND #2 |  | 1,824 | 1,599 |
| #2 | ti(epidemiolog*) OR ab(epidemiolog*) OR ti(estimat*) OR ab(estimat*) OR ti(incidence) OR ab(incidence) OR ti(prevalen*) OR ab(prevalen*) | Concept #2: Estimate/prevalence | 577,239 | 468,618 |
| #1 | ti(childlessness) OR ab(childlessness) OR ti("delayed conception") OR ab("delayed conception") OR ti(fecundability) OR ab(fecundability) OR ti(fecundity) OR ab(fecundity) OR ti("inability to conceive") OR ab("inability to conceive") OR ti(infecundity) OR ab(infecundity) OR ti(infertility) OR ab(infertility) OR ti(sterility) OR ab(sterility) OR ti(subfecundity) OR ab(subfecundity) OR ti(subfertility) OR ab(subfertility) OR ti("time to conception") OR ab("time to conception") OR ti("time to pregnancy") OR ab("time to pregnancy") | Concept #1: Infertility | 15,890 | 12,665 |

**ProceedingsFirst (OCLC)**

|  | Query | Notes | Results Sept. 29, 2020 | Results March 11, 2021 |
| --- | --- | --- | --- | --- |
| #4 | #1 AND #2 (Search limited to 1990-2020 and Title) | Updated search: Limit to 2020-2021 | 64 | 0 |
| #3 | #1 AND #2 (Search limited to 1990-2020 and Title) | Updated search: Limit to 2020-2021 | 258 | 0 |
| #2 | epidemiolog* OR estimat* OR incidence OR prevalen* | Concept #2: Estimate/prevalence | 322,293 | 24 |
| #1 | childlessness OR delayed w conception OR fecundability OR fecundity OR inability w1 conceive OR infecundity OR infertility OR sterility OR subfecundity OR subfertility OR time w1 conception OR time w1 pregnancy | Concept #1: Infertility | 12,829 | 0 |

**Google Scholar**

|  | Query | Notes | Results Sept. 29, 2020 | Results March 11, 2021 |
| --- | --- | --- | --- | --- |
| #1 | (Childlessness OR "Delayed conception" OR "Inability to conceive" OR Infecundity OR Infertility OR Sterility OR Subfecundity OR Subfertility OR "Time to conception" OR "Time to pregnancy") AND (Prevalence OR Estimate OR Incidence) | Google Scholar doesn't allow to search for many keywords, so some terms were removed from the search strings. Limited to 1990-2020, excluded patents  Updated search: Limited to "Since 2020" | 434,000  Exported the first 500 records | 27,200  Exported the first 420 records only due to reaching Google Scholar limits |

# Supplementary Table SII: Grey literature search strategy

**Strategy 1. Scanning reference lists**

**Note:** References were scanned from studies identified by the research team, advisory committee members, and articles flagged during abstract and full-text screening

| Reference | Date scanned  MM/DD/YYYY |
| --- | --- |
| Abebe MS, Afework M, Abaynew Y. Primary and secondary infertility in Africa: systematic review with meta-analysis. *Fertil Res Pract* 2020;6:20. | 03/13/2021 |
| Boerma JT, Mgalla Z. *Women and infertility in sub-Saharan Africa: a multi-disciplinary perspective*. 2001; Royal Tropical Institute: Amsterdam. | 02/02/2021 |
| Boivin J, Bunting L, Collins JA, Nygren KG. International estimates of infertility prevalence and treatment-seeking: potential need and demand for infertility medical care. *Hum Reprod* 2007;22:1506–1512. | 08/06/2020 |
| Buck GM, Lynch CD, Stanford JB, Sweeney AM, Schieve LA, Rockett JC, Selevan SG, Schrader SM. Prospective pregnancy study designs for assessing reproductive and developmental toxicants. *Environ Health Perspect* 2004;112:79–86. | 08/07/2020 |
| Chandra A, Copen CE, Stephen EH. Infertility and impaired fecundity in the United States, 1982-2010: data from the National Survey of Family Growth. *Natl Health Stat Report* 2013;67:1–18. | 09/22/2020 |
| Direkvand Moghadam A, Delpisheh A, Sayehmiri K. The prevalence of infertility in Iran: a systematic review. *Iran J Obstet Gynecol Infertil* 2013;16:1–7. Mashhad University of Medical Sciences. | 01/09/2021 |
| Dyer SJ. Comment to: International estimates on infertility prevalence and treatment seeking: potential need and demand for medical care. *Hum Reprod* 2009;24:2379–2380. | 08/25/2020 |
| Eldib A, Tashani OA. Infertility in the Middle East and North Africa Region: a systematic review with meta-analysis of prevalence surveys. *Libyan J Med Sci* 2018;2:37. Medknow Publications. | 01/09/2021 |
| Gurunath S, Pandian Z, Anderson RA, Bhattacharya S. Defining infertility: a systematic review of prevalence studies. *Hum Reprod Update* 2011;17:575–588. | 08/06/2020 |
| Islam MM, Yadava RC. Estimation of fecundability: levels and trends in Bangladesh. *J Fam Welf* 1997;43:13–21. | 01/09/2021 |
| Katon JG, Zephyrin L, Meoli A, Hulugalle A, Bosch J, Callegari L, Galvan IV, Gray KE, Haeger KO, Hoffmire C, *et al.* Reproductive health of women veterans: a systematic review of the literature from 2008 to 2017. *Semin Reprod Med* 2018;36:315–322. | 08/06/2020 |
| Khan ME, Kumar N, Patel BC, Sikri S, George P. Infertility: its causes and consequences in Indian scenario. In Puri C, Van Look P, editors. *Sexual and reproductive health: recent advances and future directions* 2001;2:309–328. New Age International: New Delhi. | 01/09/2021 |
| Larsen U. Research on infertility: which definition should we use? *Fertil Steril* 2005;83:846–852. | 9/22/2020 |
| Mascarenhas MN, Flaxman SR, Boerma T, Vanderpoel S, Mathers CD, Stevens GA. Trends in primary and secondary infertility prevalence since 1990: a systematic analysis of demographic and reproductive health surveys. *The Lancet* 2013;381:S90. | 9/22/2020 |
| Naz MSG, Ozgoli G, Sayehmiri K. Prevalence of infertility in Iran: a systematic review and meta-analysis. *Urol J* 2020;17:338–345. | 01/09/2021 |
| Polis CB, Cox CM, Tunçalp Ö, McLain AC, Thoma ME. Estimating infertility prevalence in low-to-middle-income countries: an application of a current duration approach to Demographic and Health Survey data. *Hum Reprod* 2017;32:1064–1074. | 9/22/2020 |
| Scheike TH, Keiding N. Design and analysis of time-to-pregnancy. *Stat Methods Med Res* 2006;15:127–140. | 01/09/2021 |
| Schmidt L, Münster K. Infertility, involuntary infecundity, and the seeking of medical advice in industrialized countries 1970-1992: a review of concepts, measurements and results. *Hum Reprod* 1995;10:1407–1418. | 08/07/2020 |
| Stephen EH. Postponement of childbearing and its effect on the prevalence of subfecundity. In Velde E te, Pearson P, Broekmans F, editors. *Female Reproductive Aging: the proceedings of the 10th Reinier de Graaf Symposium, Zeist, the Netherlands* 2000;9:59–70. | 01/09/2021 |
| Thoma ME, McLain AC, Louis JF, King RB, Trumble AC, Sundaram R, Buck Louis GM. Prevalence of infertility in the United States as estimated by the current duration approach and a traditional constructed approach. *Fertil Steril* 2013;99:1324–1331. | 9/22/2020 |
| Zhou Z, Zheng D, Wu H, Li R, Xu S, Kang Y, Cao Y, Chen X, Zhu Y, Chen Z, *et al.* Epidemiology of infertility in China: a population-based study. *BJOG* 2018;125:432–441. | 08/07/2020 |

**Strategy 2. Targeted website browsing/searching**

| Organization name | Website | Date of search  MM/DD/YY |
| --- | --- | --- |
| African Network and Registry for Assisted Reproductive Technology (ANARA) | <http://anara-africa.com/> | 08/05/2020 |
| American College of Obstetricians and Gynecologists (ACOG) | <https://www.acog.org/> | 08/05/2020 |
| American Society for Reproductive Medicine (ASRM) | <https://www.asrm.org/> | 08/25/2020 |
| Asia Pacific Initiative on Reproduction (ASPIRE) | <http://aspire-reproduction.org/> | 08/25/2020 |
| Centers for Disease Control (CDC) | <https://www.cdc.gov/> | 08/06/2020 |
| Demographic and Health Surveys (DHS) Program | <https://dhsprogram.com/> | 08/03/2020 |
| European Board & College of Obstetrics and Gynecology (EBCOG) | <https://www.ebcog.eu/> | 08/25/2020 |
| European Society of Human Reproduction and Embryology (ESHRE) | <https://www.eshre.eu/> | 08/06/2020 |
| Groupe Interafricain d’Etude, de Recherche et d’application sur la Fertilité (GIERAF) | <https://www.gieraf.org/en/> | 08/04/2020 |
| Guttmacher Institute | <https://www.guttmacher.org/> | 08/04/2020 |
| International Committee for Monitoring Assisted Reproductive Technologies (ICMART) | <https://www.icmartivf.org/> | 08/25/2020 |
| International Federation of Fertility Societies (IFFS) | <https://www.iffsreproduction.org/> |  |
| International Federation of Gynecology and Obstetrics (FIGO) | <https://www.figo.org/> | 08/06/2020 |
| National Institute of Child Health and Human Development (NICHD) | <https://www.nichd.nih.gov/> | 08/06/2020 |
| Population Council | <https://www.popcouncil.org/> | 08/03/2020 |
| Red Latinoamericana de Reproducción Asistida (RedLARA) - Latin American Network of Assisted Reproduction | <https://redlara.com/> | 08/05/2020 |
| Society for Assisted Reproductive Technology (SART) | <https://www.sart.org/> | 08/05/2020 |
| World Health Organization (WHO)  Home page_____________________________________  Publications ____________________________________  Reproductive Health Library _______________________  IRIS___________________________________________  WHOLIS _______________________________________ | <https://www.who.int/>  <https://www.who.int/publications/>  <https://extranet.who.int/rhl>  <https://apps.who.int/iris/>  <http://kohahq.searo.who.int/> | 08/05/2020 |

**Strategy 3. Conference proceedings**

| Conference name | Website | Proceedings available Date searched  MM/DD/YYYY |
| --- | --- | --- |
| African Population Conference (APS) organized by Union for African Population Studies (UAPS) | <https://uaps-uepa.org/> | No |
| American Public Health Association (APHA) Annual Meeting | <https://www.apha.org/Events-and-Meetings/Annual> | No |
| American Society for Reproductive Medicine (ASRM) Scientific Congress and Expo | <https://www.asrm.org/> | No |
| Congress of the Asia Pacific Initiative on Reproduction (ASPIRE) | <http://aspire-reproduction.org/aspire-congresses/> | No |
| European Population Conference (EPC) | <https://www.eaps.nl/page/european-population-conference> | No |
| European Society of Health and Medical Sociology (ESHMS) Conference | Each conference edition has a new website domain | No |
| Groupe Interafricain d’Etude, de Recherche et d’application sur la Fertilité (GIERAF) Congress | <https://www.gieraf.org/en/category-3/congress/> | No |
| In Vitro Fertilization World Congress of the International Society for In Vitro Fertilization (ISIVF) | <https://www.isivf.com/> | No |
| International Federation of Fertility Societies (IFFS) World Congress | <https://www.iffsreproduction.org/> | No |
| International Federation of Gynecology and Obstetrics (FIGO) World Congress | <https://www.figo.org/> | No |
| International Population Conference (IPC) organized by International Union for the Scientific Study of Population (IUSSP) | <https://iussp.org/en/conference-papers> | Yes, 09/20/2020 |
| Pacific Coast Reproductive Society (PCRS) Annual Meeting | <https://www.pcrsonline.org/> | No |
| Population Association of America (PAA) Annual Meeting | <https://www.populationassociation.org/home> | No |
| Red Latinoamericana de Reproducción Asistida (REDLARA) - Latin American Network of Assisted Reproduction Annual Conference | <https://redlara.com/> | No |
| Society for Pediatric and Perinatal Epidemiologic Research (SPER) Annual Meeting | <https://sper.org/annual-meeting-2/> | No |
| Society for Reproductive Investigation (SRI) Annual Meeting | <https://www.sri-online.org/> | No |

# Supplementary Table SIII: Data extraction items

| **READ BEFORE EXTRACTING DATA** | |
| --- | --- |
| Separate tab will be used for each type of study | - Population-based study - Clinic-based study - Sub-group study |
| Important notes regarding grouped/linked studies | Most studies will not have any notes in this column; however, for those that do have notes, it is important to read before starting the data extraction since, for some studies that are linked to other studies, only a portion of data will be extracted. |
| **REFERENCE DETAILS (Rayyan)** | |
| Reference information for article | All studies marked “Included” and “Maybe” will be exported in Excel along with the selected labels. |
| **METHODS** |  |
| **Supplementary reference** |  |
| Supplementary reference for study details | If the article does not include details about study design and/or methods but does reference another article where these details are described, then include the reference information in this cell as it is written in the in-text citation of the article so it can easily be identified if/when needed. |
| **Study characteristics** |  |
| Study questions/aim(s) | Open ended |
| Geographic unit | - World Regions - World Region - Countries - Country - Regions/provinces/states (within country) - Region/province/state (within country) - Cities/Districts/Counties - City/District/Country |
| Geographic location  - Name of country(ies), region(s)/province(s)/state(s), city(ies) | Open ended; list with comma  If multiple geographic locations included and reported separately, add a row for each one (e.g. DHS analysis reporting on multiple countries) |
| World region (WHO Regions) | - East Asia and Pacific - Europe and Central Asia - Latin America and Caribbean - Middle East and North Africa - North America - South Asia - sub-Saharan Africa - Multiple Regions - Global |
| Income level | - High income - Low- and Middle-income - Both   Link: <https://data.worldbank.org/country> (right-hand side lists income levels, if you click on one and scroll to the bottom, you will find all countries within that income level) |
| **Sampling** | |
| Sampling strategy | Open ended (e.g. simple random sampling, stratified sampling, systematic random sampling, multistage sampling, cluster sampling, census, clinic-based census, etc.) |
| Sampling frame | Open ended (e.g. clinic records, census enumeration areas, national registries, etc.) |
| For clinic-based studies only   1. Clinic/Hospital type: 2. Number of participating hospitals/clinics: | - Primary care - OB/GYN - Obstetrics - Gynecology - Other (Open ended)   Open ended |
| Sampling unit | - Individuals - Couples - Household |
| Sex of respondents | - Men - Women - Both Men and Women - Unknown |
| Eligibility criteria (for entire sample, not analytical sample) | Open ended |
| **Study design** |  |
| Study design | - Cross-sectional (*Note*: if authors use only a single wave from a longitudinal/cohort study, mark it as “cross-sectional) - Cohort - Case-control - Randomized trial   If multiple study designs included, add a row for each one (e.g. study includes data from two different data sets and provides estimates from each data set) |
| Data type | - Primary data - Secondary data (Data source: Open ended) |
| **Data collection** |  |
| Data collection dates | MM/DD/YYYY |
| Data collection instrument(s) | - Survey   - *Mode of administration*: online, face-to-face, mail, phone, not described, not applicable)   - *Administrator*: interviewer (with or without assisted software), self-administered (with or without assisted software), not described in manuscript, not applicable - Chart review - Other (Open ended) - Not applicable |
| **Infertility definition applied** |  |
| Ratio measured (term used to define “infertility”) | Open ended |
| Definition (if specific wording identified, use exacting wording) | Open ended   - If multiple definitions used, add a row for each definition - If a study reports primary and secondary infertility as a subset of the total, you do not need to include separate rows for each. In these instances, total, primary, and secondary infertility can be reported on the same row (columns CF - CO). However, if different durations are used to define primary and secondary infertility, then separate rows can be used. (e.g. Study ID 888). |
| Duration (months) | 6, 12, 24, 36, 60, other, no duration  If other or no duration, specify: Open ended |
| Outcome measured | - Failure to achieve clinical pregnancy - Failure to achieve live birth - Other (Open ended) |
| Numerator  (this will often be identical to the definition of infertility) | Open ended |
| Intentions are part of numerator  (i.e. trying to conceive) | Yes, No, Don’t Know |
| Denominator | Open ended |
| Intentions are part of denominator  (i.e. trying to conceive) | Yes, No, Don’t Know |
| Denominator only includes those ever at risk of pregnancy  (e.g. denominator only includes women who are having unprotected sex and/or report trying for pregnancy. If authors use “marriage” as a proxy for unprotected sex, mark “don’t know” since some of these women might actually be practicing contraception or abstinence and then include your rationale in the next cell) | Yes, No, Don’t Know  If “don’t know,” provide explanation |
| Questions used to derive data | Open ended (include exact wording, if available) |
| Analytic sample   1. Exclusion criteria 2. Age range 3. Relationship status | Open ended  Open ended  Open ended |
| How participants with unintended pregnancies were handled? | Open ended |
| Among participants included in the analytic sample, how were those with known infertility were handled?  (e.g. congenital absence of the vas deferens) | Open ended |
| How were participants who went on to receive fertility treatment handled? | Open ended |
| **Analytic methods used to generate the estimates** | |
| Type of measure | - Lifetime (i.e. ever experienced infertility in their lifetime) - Point - prevalence - current (i.e. currently experiencing infertility) - Point - prevalence - past (i.e. experienced infertility at a specific time in the past such as the first year of marriage) |
| Multiple methods used to estimate infertility prevalence within the same study population | - Yes (add a row for each method) - No |
| Method(s) used | - Prospective TTP - Retrospective TTP - Current Duration Approach - Retrospective Non-TPP (Direct) - Retrospective Indirect - Medical Record/Admissions Review - Other (please specify) |
| Notes/details about the application of the method | Open ended  (e.g. description of how the TTP or current duration was derived; statistical methods used, etc.) |
| Reported analytic assumptions | Open ended |
| Reported strengths of the method | Open ended |
| Reported limitations of the method | Open ended |
| **RESULTS** | |
| **Sample** |  |
| Sample size | Open-ended |
| Response rate overall | Open-ended |
| Response rate for sub-groups ( if applicable) | Open-ended |
| Comparison made of characteristics of non-responders to responders | Yes, No/Not reported in manuscript |
| Reasons for non-response | Open-ended |
| Analytic sample size | Open-ended |
| **Participant characteristics for the entire sample**  **(when available or applicable; extract as reported in each article, note “n/a” if not reported)** | |
| Sex | Open-ended |
| Relationship status | Open-ended |
| Urban/Rural |  |
| Parity | Open-ended |
| Gravidity | Open-ended |
| Age | Open-ended |
| Race/Ethnicity | Open-ended |
| Sexual orientation | Open-ended |
| Frequency of sexual intercourse | Open-ended |
| **Infertility estimates** | |
| Overall estimates | N, prevalence/incidence and 95% confidence interval or standard error  If authors provide estimates for multiple years, record the most recent year here and then note the other years in the cell titled, “List of additional estimates available by participant characteristics/sub-groups) |
| Primary infertility definition | - No prior pregnancy - No prior live birth - Not defined |
| Primary infertility estimates (if applicable) | N, prevalence/incidence and 95% confidence interval or standard error |
| Secondary infertility definition | - Prior pregnancy - Prior live birth - Not defined |
| Secondary infertility estimates (if applicable) | N, prevalence/incidence and 95% confidence interval or standard error |
| Proportion experiencing both primary and secondary infertility (if applicable) | N, prevalence/incidence and 95% confidence interval or standard error |
| List of additional estimates available by participant characteristics/sub-groups/less recent years | Open ended |
| Percentage of sample and/or women with infertility that used treatment: Indicate if among full sample or only those with infertility and report | N, prevalence/incidence and 95% confidence interval or standard error |
| **Additional information and notes** | |
| Quality assessment complete | Yes/No |
| Comments or data not extracted elsewhere | Open-ended |
| Requires follow-up with author(s) to obtain missing information | - Yes (if information is missing or unclear on the definition/numerator/denominator, methods applied, or prevalence estimates - N, n, or 95% CI)   - Specify what information is needed   - Dates email(s) sent to author - No |
| If you believe article should be excluded, shade the row(s) in gray and state your reason here (we may find during data extraction that some articles that we flagged for inclusion actually should be excluded) | Open ended |

# Supplementary Table SIV: Risk of bias tool

The Risk of Bias Tool is designed to assess the risk of bias in population-based prevalence studies. It was initially developed by Leboeuf-Yde and Lauritsen (1995) and then modified by Hoy et al. (2012). We slightly modified the Hoy et al. (2012) tool to align with the purpose of our study. Please read the additional notes for each item when initially using the tool.

**Note:** If there is insufficient information in the article to permit a judgement for a particular item, please answer No (HIGH RISK) for that particular item.

| **Item #** | **Risk of bias item** | **Criteria for answers** | **Additional notes and examples** |
| --- | --- | --- | --- |
|  | **External validity** | | |
| **1** | Was the sampling frame a true or close representation of the target population? | - Yes (LOW RISK): The sampling frame was a true or close representation of the target population. - No (HIGH RISK): The sampling frame was NOT a true or close representation of the target population. | The sampling frame is a list of the sampling units in the target population and the study sample is drawn from this list. Clinic-based sampling frames are considered high risk given that they only represent those individuals seeking care.  Examples:   - The sampling frame was a list of almost every individual within the target population. The answer is: Yes (LOW RISK). - The cluster sampling method was used and the sample of clusters/villages was drawn from a list of all villages in the target population. The answer is: Yes (LOW RISK). - The sampling frame was a list of just one particular ethnic group within the overall target population, which comprised many groups. The answer is: No (HIGH RISK). - The sampling frame included all eligible patients attending a primary care clinic that serves the target population over a 12-month period. The answer is No (HIGH RISK). - The sampling frame included pregnant women only. The answer is: No (HIGH RISK). |
| **2** | Was some form of random selection used to select the sample, OR, was a census undertaken? | - Yes (LOW RISK): A census was undertaken, OR, some form of random selection was used to select the sample (e.g. simple random sampling, stratified random sampling, cluster sampling, systematic sampling). - No (HIGH RISK): A census was NOT undertaken, AND some form of random selection was NOT used to select the sample. | A census collects information from every unit in the sampling frame. Clinic-based studies that recruit all eligible patients within a time period of 12 months or longer are considered a census. In a survey, only part of the sampling frame is sampled. In these instances, random selection of the sample helps minimize study bias.  Examples:   - The sample was selected using simple random sampling. The answer is: Yes (LOW RISK). - The target population was the village and every person in the village was sampled. The answer is: Yes (LOW RISK). - A census of the patient population was taken at a clinic by sampling all eligible patients over a 12-month period. The answer is: Yes (LOW RISK). - The target population was a region within a country but only the nearest villages to the capital city were selected in order to save on the cost of fuel. The answer is: No (HIGH RISK). - In a case-control study, controls were selected to match the cases on certain characteristics such as age. The answer is: No: (HIGH RISK) |
| **3** | Was the likelihood of non-response bias minimal? | - Yes (LOW RISK): The response rate for the study was >/=75%, OR, an analysis was performed that showed no significant difference in relevant demographic characteristics between responders and no responders OR authors applied weighting methods to account for differences between responders and non-responders. - No (HIGH RISK): The response rate was <75%, and if any analysis comparing responders and non-responders was done, it showed a significant difference in relevant demographic characteristics between responders and non-responders. | Examples:   - The response rate was 83%. The answer is : Yes (LOW RISK). - The response rate was 68%; however, the researchers did an analysis and found no significant difference between responders and non-responders in terms of age, sex, occupation and or socioeconomic status. The answer is: Yes (LOW RISK). - The response rate was 68%; however, the researchers applied weighting methods to account for differences between responders and non-responders in terms of age, sex, occupation and or socioeconomic status. The answer is: Yes (LOW RISK). - The response rate was 65% and the researchers did NOT carry out an analysis to compare relevant demographic characteristics between responders and non-responders. The answer is: No (HIGH RISK). - The response rate was 69% and the researchers did an analysis and found a significant difference in age, sex and socio-economic status between responders and non-responders and no procedures were applied to account for differences. The answer is: No (HIGH RISK). |
|  | **Internal validity** | | |
| **4** | Were data collected directly from the subjects (as opposed to a proxy)? | - Yes (LOW RISK): All data were collected directly from the subjects. - No (HIGH RISK): In some instances, data were collected from a proxy. | A proxy is a representative of the subject.  Examples:   - All eligible subjects in the household were interviewed separately. The answer is: Yes (LOW RISK). - A representative of the household was interviewed and questioned about the presence of infertility in at least one household member, including his or her partner. The answer is: No (HIGH RISK). - Medical records (proxy) are used to identify those with infertility. Some individuals with known infertility will not be captured in medical records since not all patients are asked about infertility. The answer is No (HIGH RISK). |
| **5** | Was an acceptable case definition used in the study? | - Yes (LOW RISK): An acceptable case definition was used. In instances where multiple definitions are used, at least one acceptable case definition was used. - No (HIGH RISK): An acceptable case definition was NOT used. | Acceptable case definitions of infertility include those that define infertility as a failure to achieve either a clinical pregnancy or live birth after > 12 months of regular unprotected intercourse overall or > 6 months for ages 35 and over, consistent with minimum clinical criterion for defining infertility (ACOG). Given that some studies avoid use of 12 months due to heaping, definitions >9 months will be considered acceptable. Intentions may or may not be included in the definition. Definitions that include menopausal women and surgically sterile men or women are not acceptable.  Examples:   - Clinical definition: failure to achieve a clinical pregnancy after 12 months or more of regular unprotected sexual intercourse. The answer is: Yes (LOW RISK) - Epidemiological definition: failure to achieve a clinical pregnancy (or live birth) after 24 months or more of regular unprotected sexual intercourse. The answer is: Yes (LOW RISK) - Demographic definition: failure to achieve a live birth after 5 years or more of regular unprotected sexual intercourse. The answer is: Yes (LOW RISK) - Failure to achieve a clinical pregnancy after 6 months of regular unprotected sexual intercourse. The answer is: No (HIGH RISK). - Difficulty achieving a clinical pregnancy (no duration specified). The answer is: No (HIGH RISK) |
| **6** | Was the study instrument/items that measured the parameter of interest (e.g. prevalence of infertility) shown to have reliability and validity (if necessary)? | - Yes (LOW RISK): The study instruments/items had been shown to have reliability and validity (if this was necessary), e.g. test-retest, piloting, validation in a previous study, etc. - No (HIGH RISK): The study instruments/items had NOT been shown to have reliability or validity (if this was necessary). | Self-reported time-to-pregnancy/ time-trying-to-conceive instruments (retrospective or prospective) are considered low risk given that several studies have shown these measures to be fairly reliable and valid (though not all). Binary self-reported instruments that include a duration are also considered low risk (i.e. ‘Have you ever had a time, lasting 12 months or longer, when you and a partner were trying for a pregnancy but it didn’t happen?’). Studies that use ICD codes to identify cases of infertility are acceptable.  Instruments that do not specify a duration of time are considered high risk unless compared to a valid and reliable measure since the definition of infertility is duration-based and these types of measures have not been validated. Current duration measures are considered high risk since these measures have not been validated for measuring infertility. Studies that use the reproductive calendar to indirectly classify women as infertile are considered high risk. Studies that use proxy measures for unprotected sex are considered high risk (e.g. assume those that are married are having unprotected sex).  Examples:   - The authors used a reproductive calendar to determine time to pregnancy or live birth. The answer is: Yes (LOW RISK). - The authors ask participants if they ever tried for 12-months or longer to get pregnant. The answer is: Yes (LOW RISK). - The authors ask participants if they ever had difficulty conceiving and do not compare results to a valid and reliable measure. The answer is: No (HIGH RISK) - The authors use a reproductive calendar to estimate current duration at risk and do not compare to a valid and reliable measure. The answer is: No (HIGH RISK) - The authors don’t explicitly state or sufficiently describe the question (or the ICD codes) used to determine infertility status of respondents. The answer is: No (HIGH RISK) - A reproductive calendar is used to indirectly determine whether a woman is classified as infertile (no direct questions on infertility). The answer is: No (HIGH RISK). - The authors assume that all married couples are having unprotected sex. The answer is: No (HIGH RISK). |
| **7** | Was the same mode of data collection used for all subjects? | - Yes (LOW RISK): The same mode of data collection was used for all subjects. - No (HIGH RISK): The same mode of data collection was NOT used for all subjects. | The mode of data collection is the method used for collecting information from the subjects. The most common modes are face-to-face interviews, telephone interviews and self-administered questionnaires.  Examples:   - All eligible subjects had a face-to-face interview. The answer is: Yes (LOW RISK). - Some subjects were interviewed over the telephone and some filled in postal questionnaires. The answer is: No (HIGH RISK). |
| **8** | Were the numerator(s) and denominator(s) for the parameter of interest appropriate? | - Yes (LOW RISK): The paper presented appropriate numerator(s) AND denominator(s) for the parameter of interest (e.g. the prevalence of infertility). - No (HIGH RISK): The paper did present numerator(s) AND denominator(s) for the parameter of interest but one or more of these were inappropriate. | There may be errors in the calculation and/or reporting of the numerator and/or denominator.  Examples:   - There were no errors in the reporting of the numerator(s) AND denominator(s) for the prevalence of infertility. The answer is: Yes (LOW RISK). - In reporting the overall prevalence of infertility in both men and women), the authors accidentally used the population of women as the denominator rather than the combined population. The answer is: No (HIGH RISK). |
|  | **Summary assessment** | | |
| **9** | Summary item on the overall risk of study bias | - LOW RISK OF BIAS: Further research is very unlikely to change our confidence in the estimate. - MODERATE RISK OF BIAS: Further research is likely to have an important impact on our confidence in the estimate and may change the estimate. - HIGH RISK OF BIAS: Further research is very likely to have an important impact on our confidence in the estimate and is likely to change the estimate. | 1-point is awarded to each item labeled as “yes (LOW RISK). Items 1 - 8 are summed and level of risk is determined by the following tertiles:   - Low risk of bias: 6 - 8 - Moderate risk of bias: 3 - 5 - High risk of bias: 0 - 2 |

# Supplementary Table SV: Duplicate studies excluded from review

*** **Primary studies** are publications among the duplicate publications selected for inclusion in the systematic review. Primary publication were selected based on date of data collection (publications with the most recent data were prioritized), sample (publications with the largest sample were prioritized), and main purpose of study (publications with the aim of estimating prevalence of infertility were prioritized).

| Item # | Duplicate studies excluded from review | Primary studies* |
| --- | --- | --- |
| 1 | Abma JC, Chandra A, Mosher WD, Peterson LS, Piccinino LJ. Fertility, family planning, and women’s health: new data from the 1995 National Survey of Family Growth. *Vital Health Stat 23* 1997;1–114. | Chandra A, Stephen EH. Infertility and impaired fecundity in the United States, 1982–2010: data from the National Survey of Family Growth. *Natl Health Stat Report* 2013;1–19. |
| 2 | Akhondi MM, Kamali K, Ranjbar F, Shirzad M, Shafeghati S, Ardakani ZB, Goodjani A, Parsaeian M, Mohammad K. Prevalence of primary infertility in Iran in 2010. *Iran J Public Health* 2013;42:1398–1404. | Akhondi MM, Ranjbar F, Shirzad M, Ardakani ZB, Kamali K, Mohammad K. Practical difficulties in estimating the prevalence of primary infertility in Iran. *Int J Fertil Steril* 2019;**13**:113–117. |
| 3 | Arya S, Dwivedi AK, Alvarado L, Kupesic-Plavsic S. Exposure of U.S. population to endocrine disruptive chemicals (Parabens,  Benzophenone-3, Bisphenol-A and Triclosan) and their associations with female  infertility. *Environ Pollut* 2020;265:114763. | Anyalechi GE, Hong J, Kreisel K, Torrone E, Boulet S, Gorwitz R, Kirkcaldy RD, Bernstein K. Self-reported infertility and associated pelvic inflammatory disease among women of reproductive age: National Health and Nutrition Examination Survey, United States, 2013-2016. *Sex Transm Dis* 2019;**46**:446–451. |
| 4 | Bach CC, Liew Z, Bech BH, Nohr EA, Fei C, Bonefeld-Jorgensen EC, Henriksen TB, Olsen J. Perfluoroalkyl acids and time to pregnancy revisited: an update from the Danish National Birth Cohort. *Environ Health* 2015;14:59–59. | Guldbrandsen K, Håkonsen LB, Ernst A, Toft G, Lyngsø J, Olsen J, Ramlau-Hansen CH. Age of menarche and time to pregnancy. *Hum Reprod* 2014;**29**:2058–2064. |
| 5 | Barden-O’Fallon JL. An examination of self-reported fertility impairment in a rural district of Malawi. 2004; The University of North Carolina at Chapel Hill: Ann Arbor. | Barden-O’Fallon J. Associates of self-reported fertility status and infertility treatment-seeking in a rural district of Malawi. *Hum Reprod* 2005;**20**:2229–2236. |
| 6 | Basso O, Baird DD. Infertility and preterm delivery, birthweight, and Caesarean section: a study within the Danish National Birth Cohort. *Hum Reprod* 2003;18:2478–2484. | Guldbrandsen K, Håkonsen LB, Ernst A, Toft G, Lyngsø J, Olsen J, Ramlau-Hansen CH. Age of menarche and time to pregnancy. *Hum Reprod* 2014;**29**:2058–2064. |
| 7 | Bitler M, Schmidt L. Health disparities and infertility: impacts of state-level insurance mandates. *Fertil Steril* 2006;85:858–865. | Chandra A, Stephen EH. Infertility and impaired fecundity in the United States, 1982–2010: data from the National Survey of Family Growth. *Natl Health Stat Report* 2013;1–19. |
| 8 | Chandra A, Stephen EH. Impaired fecundity in the United States: 1982-1995. *Fam Plann Perspect* 1998;30:34–42. | Chandra A, Stephen EH. Infertility and impaired fecundity in the United States, 1982–2010: data from the National Survey of Family Growth. *Natl Health Stat Report* 2013;1–19. |
| 9 | Chin HB, Kramer MR, Mertens AC, Spencer JB, Howards PP. Differences in women’s use of medical help for becoming pregnant by the level of urbanization of county of residence in Georgia. *J Rural Health* 2017;33:41–49. | Jacobson MH, Chin HB, Mertens AC, Spencer JB, Fothergill A, Howards PP. “Research on infertility: definition makes a difference” Revisited. *Am J Epidemiol* 2017;**187**:337–346. |
| 10 | Craig LB, Peck JD, Janitz AE. The prevalence of infertility in American Indian/Alaska Natives and other racial/ethnic groups: National Survey of Family Growth. *Paediatr Perinat Epidemiol* 2019;33:119–125. | Chandra A, Stephen EH. Infertility and impaired fecundity in the United States, 1982–2010: data from the National Survey of Family Growth. *Natl Health Stat Report* 2013;1–19. |
| 11 | Ernst A, Lauridsen LLB, Brix N, Arah OA, Olsen J, Olsen LH, Ramlau-Hansen CH. Parental time to pregnancy, medically assisted reproduction and pubertal development in boys and girls. *Hum Reprod* 2019;34:724–732. | Guldbrandsen K, Håkonsen LB, Ernst A, Toft G, Lyngsø J, Olsen J, Ramlau-Hansen CH. Age of menarche and time to pregnancy. *Hum Reprod* 2014;**29**:2058–2064. |
| 12 | Fei C, McLaughlin JK, Lipworth L, Olsen J. Maternal levels of perfluorinated chemicals and subfecundity. *Hum Reprod* 2009;24:1200–1205. | Guldbrandsen K, Håkonsen LB, Ernst A, Toft G, Lyngsø J, Olsen J, Ramlau-Hansen CH. Age of menarche and time to pregnancy. *Hum Reprod* 2014;**29**:2058–2064. |
| 13 | Filippov OS, Radionchenko AA. [The causes of male sterility in Siberia]. *Urol Nefrol (Mosk)* 1997;33–34. | Philippov OS, Radionchenko AA, Bolotova VP, Voronovskaya NI, Potemkina TV. Estimation of the prevalence and causes of infertility in western Siberia. *Bull World Health Organ* 1998;**76**:183–187. |
| 14 | Fledderjohann J. Difficulties conceiving and relationship stability in sub-Saharan Africa: the case of Ghana. *Eur J Popul* 2017;33:129–152. | Fledderjohann J, Johnson DR. Impaired fertility and perceived difficulties conceiving in Ghana: measurement problems and prospects. *J Biosoc Sci* 2016;**48**:431–456. |
| 15 | Győrffy Z, Girasek E. [The health of female physicians in Hungary. A longitudinal perspective]. *Orv Hetil* 2014;155:993–999. | Győrffy Z, Dweik D, Girasek E. Reproductive health and burn-out among female physicians: nationwide, representative study from Hungary. *BMC Womens Health* 2014;**14**:121. |
| 16 | Hu Y, Ji L, Zhang Y, Shi R, Han W, Lap Ah Tse, Rui Pan, Yiwen Wang, Guodong Ding, Jian Xu, *et al.* Organophosphate and pyrethroid pesticide exposures measured before conception and associations with time to pregnancy in Chinese couples enrolled in the Shanghai birth cohort. *Environ Health Perspect* 2018;126:1–9. | Hu P, Cai C, Vinturache A, Hu Y, Gao Y, Zhang J, Lu M, Gu H, Qiao J, Tian Y, *et al.* Maternal preconception body mass index and time-to-pregnancy in Shanghai Women, China. *Women Health* 2020;**60**:1014–1023. |
| 17 | Huang J -t, Tang Y -g., Wang Q -l. Prevalence of infertility and its related factors among newlyweds in minority area. *Chinese Journal of Public Health* 2011;27:424–426. | Huang J -t, Tang Y -g. Incidence of infertility and its influencing factors among married residents in Guangdong province. *China Public Health = Zhongguo Gong Gong Wei Sheng* 2013;**29**:0194–0197. |
| 18 | Joham AE, Teede HJ, Ranasinha S, Zoungas S, Boyle J. Prevalence of infertility and use of fertility treatment in women with polycystic ovary syndrome: data from a large community-based cohort study. *J Womens Health* 2015;24:299–307. | Herbert D, Lucke J, Dobson A. Infertility, medical advice and treatment with fertility hormones and/or in vitro fertilisation: a population perspective from the Australian Longitudinal Study on Women’s Health. Aust N Z J Public Health 2009;**33**:358–364. |
| 19 | Jørgensen KT, Specht IO, Lenters V, Bach CC, Rylander L, Jönsson BA, Lindh CH, Giwercman A, Heederik D, Toft G, *et al.* Perfluoroalkyl substances and time to pregnancy in couples from Greenland, Poland and Ukraine. *Environ Health* 2014;13:116–116. | Toft G, Axmon A, Giwercman A, Thulstrup AM, Rignell-Hydbom A, Pedersen HS, Ludwicki JK, Zvyezday V, Zinchuk A, Spano M, *et al.* Fertility in four regions spanning large contrasts in serum levels of widespread persistent organochlorines: a cross-sectional study. *Environ Health* 2005;**4**:26. |
| 20 | Juhl M, Nyboe Andersen A-M, Grønbæk M, Olsen J. Moderate alcohol consumption and waiting time to pregnancy. *Hum Reprod* 2001;16:2705–2709. | Guldbrandsen K, Håkonsen LB, Ernst A, Toft G, Lyngsø J, Olsen J, Ramlau-Hansen CH. Age of menarche and time to pregnancy. *Hum Reprod* 2014;**29**:2058–2064. |
| 21 | Kapiga S, Sam NE, Mlay J, Aboud S, Ballard RC, Shao JF, Larsen U. The epidemiology of HIV-1 infection in northern Tanzania: results from a community-based study. *AIDS Care* 2006;18:379–387. | Larsen U. Research on infertility: which definition should we use? *Fertil Steril* 2005;**83**:846–852. |
| 22 | Klinger EV, Kapiga SH, Sam NE, Aboud S, et al. A community-based study of risk factors for trichomonas vaginalis infection among women and their male partners in Moshi urban district, Northern Tanzania. *Sex Transm Dis* 2006;33:712–718. | Larsen U. Research on infertility: which definition should we use? *Fertil Steril* 2005;**83**:846–852. |
| 23 | Kyrklund-Blomberg NB, Gennser G, Cnattingius S, Kyrklund-Blomberg NB, Gennser G, Cnattingius S. Placental abruption and perinatal death. *Paediatr Perinat Epidemiol* 2001;15:290–297. | Akre O, Cnattingius S, Bergström R, Kvist U, Trichopoulos D, Ekbom A. Human fertility does not decline: evidence from Sweden. *Fertil Steril* 1999;**71**:1066–1069. |
| 24 | Larsen U. Trends in infertility in Cameroon and Nigeria. *Int Fam Plann Persp* 1995;21:138-142, 166. | Larsen U. Primary and secondary infertility in sub-Saharan Africa. *Int J Epidemiol* 2000;**29**:285–291. |
| 25 | Larsen U. Childlessness, subfertility, and infertility in Tanzania. *Stud Fam Plann* 1996;27:18–28. | Larsen U. Primary and secondary infertility in sub-Saharan Africa. *Int J Epidemiol* 2000;**29**:285–291. |
| 26 | Larsen U. Fertility in Tanzania: do contraception and sub-fertility matter? *Popul Stud* 1997;51:213–220. | Larsen U. Primary and secondary infertility in sub-Saharan Africa. *Int J Epidemiol* 2000;**29**:285–291. |
| 27 | Larsen U. Sterility in sub-Saharan Africa. *Popul Stud* 1994;48:459–474. | Larsen U. Primary and secondary infertility in sub-Saharan Africa. *Int J Epidemiol* 2000;**29**:285–291. |
| 28 | Lee S, Min J-Y, Kim H-J, Min K-B. Association between the frequency of eating non-home-prepared meals and women infertility in the United States. *J Prev Med Public Health* 2020;53:73–81. | Anyalechi GE, Hong J, Kreisel K, Torrone E, Boulet S, Gorwitz R, Kirkcaldy RD, Bernstein K. Self-reported infertility and associated pelvic inflammatory disease among women of reproductive age: National Health and Nutrition Examination Survey, United States, 2013-2016. *Sex Transm Dis* 2019;**46**:446–451. |
| 29 | Li Q, Zheng D, Wang Y, Li R, Wu H, Xu S, Kang Y, Cao Y, Chen X, Zhu Y, *et al.* Association between exposure to airborne particulate matter less than 2.5 μm and human fecundity in China. *Environ Int* 2021;146:106231. | Zhou Z, Zheng D, Wu H, Li R, Xu S, Kang Y, Cao Y, Chen X, Zhu Y, Chen Z, *et al.* Epidemiology of infertility in China: a population-based study. *BJOG* 2018;**125**:432–441. |
| 30 | Menken J, Larsen U. Estimating the incidence and prevalence and analyzing the correlates of infertility and sterility. *Ann N Y Acad Sci* 1994;709:249–265. | Larsen U. Primary and secondary infertility in sub-Saharan Africa. *Int J Epidemiol* 2000;**29**:285–291. |
| 31 | Mirzaei M, Ph.D., Namiranian N, Bagheri-Fahraji B, Gholami S. Infertility and physical activity: A cross-sectional study of women living in Yazd aged 20-49 yr, 2014-2015. *Int J Reprod Biomed* 2020;18:795–803. | Mirzaei M, Namiranian N, Dehghani Firouzabadi R, Gholami S. The prevalence of infertility in 20-49 years women in Yazd, 2014-2015: a cross-sectional study. *Int J Reprod Biomed* 2018;**16**:683–688. |
| 32 | Oakley L, Doyle P, Maconochie N. Lifetime prevalence of infertility and infertility treatment in the UK: results from a population-based survey of reproduction. *Hum Reprod* 2008;23:447–447. | Oakley LL. The epidemiology of infertility: measurement, prevalence and an investigation of early life and reproductive risk factors. 2010; London School of Hygiene & Tropical Medicine. |
| 33 | Rostad B, Schei B, Sundby J. Fertility in Norwegian women: results from a population‐based health survey. *Scand J Public Health* 2006;34:5–10. | Rostad B, Schmidt L, Sundby J, Schei B. Has fertility declined from mid-1990s to mid-2000s? *Acta Obstet Gynecol Scand* 2013;**92**:1284–1289. |
| 34 | Schmuhl NB, Mooney KE, Zhang X, Cooney LG, Conway JH, LoConte NK. No association between HPV vaccination and infertility in U.S. females 18–33 years old. *Vaccine* 2020;38:4038–4043. | Anyalechi GE, Hong J, Kreisel K, Torrone E, Boulet S, Gorwitz R, Kirkcaldy RD, Bernstein K. Self-reported infertility and associated pelvic inflammatory disease among women of reproductive age: National Health and Nutrition Examination Survey, United States, 2013-2016. *Sex Transm Dis* 2019;**46**:446–451. |
| 35 | Shafi H, Agajani Delavar M, Esmaeilzadeh S. Comparing the prevalence of infertility in urban and rural areas in Babol. *Journal of Mazandaran University of Medical Sciences* 2016;25:335–339. Journal of Mazandaran University of Medical Sciences. | Esmaeilzadeh S, Delavar MA, Zeinalzadeh M, Mir M-RA. Epidemiology of infertility: a population-based study in Babol, Iran. *Women Health* 2012;**52**:744–754. |
| 36 | Slama R, Eustache F, Ducot B, Jensen TK, Jørgensen N, Horte A, Irvine S, Suominen J, Andersen AG, Auger J, *et al.* Time to pregnancy and semen parameters: a cross-sectional study among fertile couples from four European cities. *Hum Reprod* 2002;17:503–515. | Jensen TK, Slama R, Ducot B, Suominen J, Cawood EHH, Andersen AG, Eustache F, Irvine S, Auger S, Jouannet P, *et al.* Regional differences in waiting time to pregnancy among fertile couples from four European cities. *Hum Reprod* 2001;**16**:2697–2704. |
| 37 | Specht IO, Bonde JP, Toft G, Lindh CH, Jönsson BAG, Jørgensen KT. Serum phthalate levels and time to pregnancy in couples from Greenland, Poland and Ukraine. *PLoS One* 2015;10:e0120070. | Toft G, Axmon A, Giwercman A, Thulstrup AM, Rignell-Hydbom A, Pedersen HS, Ludwicki JK, Zvyezday V, Zinchuk A, Spano M, *et al.* Fertility in four regions spanning large contrasts in serum levels of widespread persistent organochlorines: a cross-sectional study. *Environ Health* 2005;**4**:26. |
| 38 | Stephen EH, Chandra A. Declining estimates of infertility in the United States: 1982-2002. *Fertil Steril* 2006;86:516–523. | Chandra A, Stephen EH. Infertility and impaired fecundity in the United States, 1982–2010: data from the National Survey of Family Growth. *Natl Health Stat Report* 2013;1–19. |

**Reference:**

Dijkers M. Duplicate publications and systematic reviews: problems and proposals. *KT Update* 2018;**6**:1–12.

# Supplementary Table SVI: Summary of included studies by region

| **Authors (year)** | **Geographic location** | **Type of study** | **Analytic sample size** | **Age Range** | **Methodologic approach** | **Numerator** | **Denominator** | **Period or lifetime measure** | **Duration (months)** | **Ratio measured** | **Estimate % (95% CI)** |
| --- | --- | --- | --- | --- | --- | --- | --- | --- | --- | --- | --- |
| **AFRICA REGION** | | | | | | | | | | | |
| Somé et al. (2016) | Ouagadougou, Burkina Faso | Cross-sectional | Households: 480 | Women: 18 - 45  Men: 18 - 55 | Retrospective Time-to-Pregnancy (TTP) Design | Childbearing age women/men who have never born a child and who had been seeking a child for more than 12 months | All women/men in union/living with partner | Lifetime | 12 | Infertility - women  Primary infertility  Secondary infertility   Infertility - men Primary infertility Secondary infertility | 10.4 (7.9-13.5) 6.8 (4.8-9.4) 3.6 (2.2-5.7)  9.3 (7.0-12.2) 4.8 (3.2-7.2) 4.4 (2.9-6.7) |
| Sundby et al. (1998) | Gambia | Cross-sectional | 3000 | 15 - 49 | Self-reported infertility measure (Direct) | Women with no pregnancy or live children born despite being married and not having used family planning for at least a year | All women | Period | 12 | Infertility Primary infertility | 9.5 3.3 |
|  |  |  |  |  |  | Women who are married, not using contraceptives and not breastfeeding and have had no birth of a child for the last three years. | All women | Period | 36 | Secondary subfertility | 6.2 |
| Walraven et al. (2001) | Farafenni area, The Gambia | Cross-sectional | 871 | < 45 years | Self-reported infertility measure (Direct) | Women trying to conceive for at least one year without success despite regular (one time per week) sexual intercourse, no use of contraception, postmenarchal and premenopausal. | Married women not using contraception, postmenarchal and premenopausal | Period | 12 | Infertility | 9·8 (8·2–11·6) |
| Geelhoed et al. (2002) | Berekum District Ghana | Cross-sectional | Women: 1073 Men: 1064 | Reproductive age | Self-reported infertility measure (Direct) | Women/men who experienced failure to achieve conception after at least one year of exposure | All women/men | Lifetime | 12 | Infertility - women Primary infertility Secondary infertility  Infertility - men Primary infertility Secondary infertility | 11.8 0.6 11.2  15.8 6.8 9.0 |
| Fledderjoha-nn and Johnson (2016) | Western, Central, and Greater Accra regions, Ghana | Cohort | 1350 | 15 - 49 | Self-reported infertility measure (Direct) | Women reporting difficulties conceiving (takes a long time to get pregnant when they want to and/or can no longer become pregnant)   Unadjusted: Does not account for contraceptive use or fertility desires  Adjusted: Women who desire a child and are not currently using modern contraception | Married or in union women | Period | No duration | Self-assessed difficulties conceiving:  Unadjusted Adjusted | 65.0 20.0 |
|  |  |  |  |  | Constructed infertility measure (Indirect) | Woman without a birth after 12/24/60/84 months   Unadjusted: Does not account for contraceptive use or fertility desires  Adjusted: Women who desire a child and are not currently using modern contraception | Women in union | Period | 12 24 60 80  12 24 60 80 | Infertility - Unadjusted     Adjusted | 69.0 64.0 35.0 24.0  17.0 15.0 7.0 4.0 |
| Miller-Fellows et al. (2017) | Nganja village, Kenya | Cross-sectional | 160 | 15 - 45 | Self-reported infertility measure (Direct) | Women who were in a sexual union and not using contraception for at least 5 years and did not have a live birth and/or women who reported a period of over one year without a pregnancy with regular, unprotected sexual intercourse | Women who had ever been married or in a co-residing sexual union, were currently pregnant, and/or had given birth to at least one child. | Lifetime | 60 or 12 | Subfertility  Primary infertility Secondary infertility | 44.0 (37.0 – 52.0) 2.0 (0.3 – 5.0) 42.0 (35.0 – 51.0) |
| Barden- O'Fallon (2005) | Mangochi, Malawi | Cohort | Women: 678  Men: 362 | Women: 15 - 34  Men: 20 - 44 | Self-reported infertility measure (Direct) | Women/men who said they ever experienced difficulty becoming pregnant | Women/men who had ever been pregnant or tried to become pregnant | Lifetime | No duration | Perceived infertility: Women Men | 19.6 19.6 |
| Fledderjoha-nn et al. (2017) | Balaka district, Malawi | Cross-sectional | 116 | 15 - 25 | Self-reported infertility measure (Direct) | Women who reported that they had a lot or some difficulty conceiving and/or carrying a pregnancy | All women | Lifetime | No duration | Impaired fertility | 12.8 |
| Rao et al. (2018) | Catchment area of a rural health facility, Malawi | Cross-sectional | 915 | 15 - 39 | Self-reported infertility measure (Direct) | Women who had ever tried and failed to conceive a pregnancy for 2 years or longer | All women | Lifetime | 24 | Infertility | 20.0 |
| Polis et al. (2017) | Nigeria | Cross-sectional | 6340 | 18 - 44 | Current Duration Design | Couples not yet pregnant by 12/24/36 months (estimated) | Women at risk of pregnancy (18–44 years, married or cohabitating, sexually active within the past 4 weeks and not currently using contraception and had not been sterilized) | Period | 12    24    36 | Infertility Primary infertility Secondary infertility  Infertility Primary infertility Secondary infertility  Infertility Primary infertility Secondary infertility | 31.1(27.9 - 34.7) 17.4 (12.9 - 23.8) 34.1 (30.3 - 39.3)  17.7 (15.7 - 20) 10.0 (7.0 - 14.3) 19.2 (17.1 - 22.1)  11.5 (10.2 - 13.0) 6.8 (4.6 - 10) 12.3 (11.0 - 14.1) |
| Ekudayo (2020) | Nigeria | Cross-sectional | 16,922 | 15 - 49 | Constructed infertility measure (Indirect) | Women who reported 0 as the total number of children ever born, who were not pregnant at the time of the survey and were not presently using contraception | Married women or women living with a partner who reported at least 1 child or who were presently pregnant (unclear if contraceptive users were included) | Period | 24 | Infertility | 3.1 |
| Bello et al. (2010) | Potchefstroom, North-West Province, South Africa | Cross-sectional | 482 | 18 - 49 | Retrospective Time-to-Pregnancy (TTP) Design | Women with a TTP greater than 6/12/24 months for the most recent pregnancy or current pregnancy attempt (if pregnancy has not been achieved) | Women who had been pregnant and planned their most recent pregnancies and women who had never been pregnant but were trying | Period | 6 12 24 | TTP > 6 months TTP > 12 months TTP > 24 months | 50.0 32.0 17.0 |
| Pick and Obermeyer (1996) | Khayelitsha, Cape Town, South Africa | Cross-sectional | 298 | 15+ | Self-reported infertility measure (Direct) | Women not using any form of contraception at the time of the survey who reported difficulty in becoming pregnant | Women who were not using any form of contraception at the time of the survey | Lifetime | No duration | Infertility | 19.0 |
|  |  |  |  |  |  | Women with no pregnancy in the preceding five years who reported difficulty in becoming pregnant and were not using contraception at the time of the survey | Women who were not using any form of contraception at the time of the survey | Period | 60 | Infertility | 10.0 |
| Larsen (2005) | Moshi Town, Tanzania | Cross-sectional | 993 | 20 - 44 | Retrospective Time-to-Pregnancy (TTP) Design | Women who have been having intercourse without using contraception or trying in any way to delay or avoid getting pregnant for at least two years without conceiving | Women in first union (married or consensual union) for at least two years | Period | 24 | Infertility  Primary infertility Secondary infertility | 12.1 (9.4 - 14.8) 2.5 (1.5 - 3.5) 9.6 (7.3 - 11.9) |
|  |  |  |  |  |  | Women who have tried to conceive for at least two years | Women in first union (married or consensual union) for at least two years | Period | 24 | Infertility  Primary infertility Secondary infertility | 6.9 (5.2 - 8.6) 1.8 (0.9 - 2.7) 5.0 (3.5 - 6.5) |
|  |  |  | 1120 | 20 - 44 | Self-reported infertility measure (Direct) | Women who report ever having problems getting pregnant | Women in first union (married or consensual union) | Lifetime | No duration | Infertility  Primary infertility Secondary infertility | 10.3 (8.4 - 12.2) 2.9 (1.9 - 3.9) 7.4 (6.0 - 8.8) |
|  |  |  | 720 | 20 - 44 | Constructed infertility measure (Indirect) | Women who have had no births at least 5 years subsequent to last birth or marriage, if childless. | Women in first union (married or consensual union) for at least five years | Period | 60 | Infertility Secondary infertility | 11.5 (9.2 - 13.7) 11.1 (9.8 - 12.6) |
|  |  |  |  |  |  | Women who have had no births at least 5 years subsequent to last birth or marriage, if childless despite confirming that she wants a(nother) child | Women in first union (married or consensual union) for at least five years | Period | 60 | Infertility Secondary infertility | 5.5 (3.9 - 7.1) 4.8 (3.9 - 5.7) |
|  |  |  |  |  |  | Women married at least five/seven years without ever having a child | Women in first union (married or consensual union) for at least five/seven years | Lifetime | 60 84 | Primary infertility | 3.5 (2.3 - 4.7) 1.9 (0.8 - 3.0) |
| Bernhard et al. (2000) | Magoda and Mpapayu villages, Tanzania | Cross-sectional | 530 | > 15 | Self-reported infertility measure (Direct) | Women with unsuccessful attempt to conceive for more than 6 months | All women | Lifetime | 6 | Primary infertility Secondary infertility | 1.5 5.1 |
| Klouman et al. (2005) | Oria village, Kilimanjaro region, Tanzania | Cross-sectional | 636 | 15 - 44 | Undetermined | Women who were unable to become pregnant within a year of living with their partners | All women | Period | 12 | Infertility  Primary infertility Secondary infertility | 10.3 3.1 7.2 |
| Larsen (2003) | Cameroon, Central African Republic (CAR), Gabon, and Chad | Cross-sectional | Cameroon: 3091 CAR: 3783 Chad: 5068 Gabon: 3205 | 15 - 49 | Constructed infertility measure (Indirect) | Childless women married for at least five years | Women married for at least five years (current contraceptive users classified as fertile) | Period | 60 | Primary infertility (range) | 3.1 - 6.9 |
|  |  |  |  |  |  | Childless women married for at least five years | Women married for at least five years (contraceptive use not taken into account) | Period | 60 | Primary infertility (range) | 3.2 - 7.0 |
|  |  |  | Cameroon: 2819 CAR: 3219 Chad: 4418 Gabon: 3134 | 20 - 44 |  | Ever-married, parous women who have had no live births during the last 5 years before the interview | Ever-married women who had at least one child and had been observed at least 5 years subsequent to the birth date of their first child (contraceptive users classified as fertile) | Period | 60 | Secondary infertility (range) | 18.9 - 26.3 |
|  |  |  |  |  |  | Ever-married, parous women who have had no live births during the last 5 years before the interview | Ever-married women who had at least one child and had been observed at least 5 years subsequent to the birth date of their first child (contraceptive use not taken into account) | Period | 60 | Secondary infertility (range) | 19.1 - 29.4 |
| Larsen (2000) | Multiple countries (22 countries with data in or after 1990) | Cross-sectional | Range: 1361 - 5869 | 20 - 44 | Constructed infertility measure (Indirect) | Women who have had no livebirths during the last 5 (secondary)/7 (primary) years before censoring (i.e., the month of survey or the month of last sexual intercourse, whichever came first) | Ever-married women (contraceptive users classified as fertile) | Period | 60/84 60 | Infertility: Range Secondary: Range | 7.0 - 28.0 7.0 - 25.0 |
|  |  |  |  |  |  | Women who have had no livebirths during the last 5 (secondary)/7 (primary) years before censoring (i.e., the month of survey or the month of last sexual intercourse, whichever came first) | Ever-married women (contraceptive use not taken into account) | Period | 60/84 84 60 | Infertility: Range Primary: Range Secondary: Range | 7.0 - 29.0 1.0 - 6.0 7.0 - 26.0 |
| Ericksen and Brunette (1996) | Multiple countries (12 countries with data in or after 1990) | Cross-sectional | Range: 1499 - 6206 | 20 - 41 | Constructed infertility measure (Indirect) | Women who had been married or sexually experienced without a birth for at least 5/7years | All women exposed to conception | Period | 60 84 | Infertility: Range | 8.9 - 16.6 12.0 - 20.8 |
| Woodall and Kramer (2018) | Ethiopia, Kenya, Tanzania, and Uganda | Cross-sectional | 17,547 | 15 - 49 | Constructed infertility measure (Indirect) | Women with no live birth within the last 5 years who have been married or in union for at least five years, are not using contraception, and are not currently pregnant | Women married or in union for at least 5 years, not using a contraceptive, and not presently pregnant | Period | 60 | Infertility Primary infertility | 35.0 3.0 |
| **EASTERN MEDITERRANEAN REGION** | | | | | | | | | | | |
| Hassan (1997) | Egypt | Cross-sectional | 20,002 | < 50 years | Constructed infertility measure (Indirect) | Women who have been exposed to or at risk of pregnancy for successive 12 months or more without conceiving | Married women | Period | 12 | Infertility Primary infertility Secondary infertility | 12.0 4.3 7.7 |
| Kazemijaliseh et al. (2015) | Tehran, Iran | Cross-sectional | 1067 | 18 - 57 | Retrospective Time-to-Pregnancy (TTP) Design | Women failing to achieve a clinical pregnancy after 12 months or more of regular unprotected sexual intercourse | Married women willing to become pregnant | Lifetime | 12 | Primary infertility | 17.3 |
| Nasrabad et al. (2013) | Iran | Cross-sectional | 90,141 | 15 - 49 | Constructed infertility measure (Indirect) | Couples of reproductive age who are having sexual intercourse without contraception and are unable to establish a pregnancy within one year | Ever-married women | Period | 12 | Primary infertility | 2.3 |
|  |  |  |  |  |  | Sexually active women who are not using a contraception but are unable to have a live birth for five or more years | Ever-married women | Period | 60 | Primary infertility | 2.6 |
| Esmaeilzadeh et al. (2012) | Babol County, Iran | Cross-sectional | 1081 | 20 - 45 | Self-reported infertility measure (Direct) | Women who have experienced a delay in conception for least 12 months of unprotected intercourse at some time in their life | Women who attempted conception | Lifetime | 12 | Infertility  Primary infertility Secondary infertility Experienced both | 15.5 (13.5 - 17.5) 12.2 1.9 1.5 |
|  |  |  |  |  |  | Women who were currently experiencing a delay in conception for least 12 months of unprotected intercourse and had not previously given birth to a child | Women who attempted conception | Period | 12 | Primary Infertility | 4.3 (2.3 - 6.3) |
| Mirzaei et al. (2018) | Yazd, Iran | Cross-sectional | 2611 | 20 - 49 | Self-reported infertility measure (Direct) | Women who have failed to achieve clinical pregnancy after 12 months or more unprotected coitus | All married, divorced, or widowed women | Period | 12 | Infertility Primary infertility Secondary infertility | 5.2(4.3 - 6.1) 2.68 (2.4 - 3.8) 2.15 (1.89 - 3.4) |
| Safarinejad (2008) | Iran | Cross-sectional | 11,441 | 15-50 | Undetermined | Women who did not conceive despite cohabitation and exposure to pregnancy for two years | Women who ever cohabitated for at least two years | Lifetime | 24 | Infertility Primary infertility Secondary infertility | 8.0 (3.2 - 15.0) 4.6 (3.6 - 5.2) 3.4 (2.4 - 5.1) |
| Vahidi et al. (2009) | Iran | Cross-sectional | 10,662 | 19-49 | Constructed infertility measure (Indirect) | Ever-married women who have experienced of infertility (no pregnancy) despite one year of unprotected intercourse | Ever-married women | Lifetime | 12 | Primary infertility | 24.9 (23.5-26.2) |
|  |  |  | 10,873 |  |  | Women who meet the definition of lifetime primary infertility and have not conceived up to the study time | Ever-married women | Period | 12 | Primary infertility | 3.4 (3.0-3.8.0) |
| Ahmadi Asr Badr et al. (2006) | Tabriz, Iran | Cross-sectional | 3183 | Not reported | Retrospective Time-to-Pregnancy (TTP) Design | Women with no conception during marriage after at least 12 months' period of intercourse without using contraception | Women married for at least one year | Lifetime | 12 | Infertility Primary infertility Secondary infertility | 3.27 2.04 1.23 |
|  |  |  | 2623 | 15-49 |  | Women with no conception during marriage after at least 12 months' period of intercourse without using contraception | Women ages 15 - 49 years married for at least one year | Lifetime | 12 | Infertility Primary infertility Secondary infertility | 3.35 2.05 1.30 |
| Akhondi et al. (2019) | Iran | Cross-sectional | 17,178 | 20-40 | Constructed infertility measure (Indirect) | Women who are sexually active, do not use any contraception, and do not have a live birth after 12/24/36/48/60 months | Married women | Lifetime | 12 24 36 48 60 | Primary infertility | 20.2 (SE = 0.2) 12.5 10.3 9.6 9.20 |
| Dovom et al. (2014) | Golestan, Qazvin, Kermanshah, Hormozgan Provinces, Iran | Cross-sectional | 888 | 18 - 49 | Retrospective Time-to-Pregnancy (TTP) Design | Women who have not become pregnant after at least 1 year of unprotected intercourse | Women who had attempted to get pregnant for at least one year, not use a contraceptive method, and have regular unprotected sexual intercourse | Period | 12 | Primary Infertility | 6.4 (4.8 - 8.0) |
|  |  |  | 681 |  |  | Women who failed to achieve a second clinical pregnancy after 12 months or more of regular unprotected sexual intercourse whether or not having the second child | Women who had attempted to get pregnant for the second time for at least one year, not use a contraceptive method, and have regular unprotected sexual intercourse | Lifetime | 12 | Secondary Infertility | 7.8 (6.0 - 9.6) |
|  |  |  | 888 |  |  | Women with any delay of more than one year to get pregnant during their life regardless of whether or not they have a child now | Women married for at least one year who have ever had a willingness for pregnancy | Lifetime | 12 | Infertility | 21.2 (18.4 - 23.8) |
| Hosseini et al. (2012) | Kermanshah, Golestan, Isfahan, and Hormozgan Provinces, Iran | Cross-sectional | 2296 | 18 - 49 | Self-reported infertility measure (Direct) | Couples with lack of pregnancy after one year of continuous unprotected sex during the ovulation point of menstrual cycle | Married women at risk of pregnancy | Period | 12 | Primary infertility Secondary infertility | 3.2 1.7 |
| Sharif et al. (2020) | Bandar Abbas,Hormozgan province, Iran | Cross-sectional | 1469 | 18 - 45 | Self-reported infertility measure (Direct) | Couples who had not achieved pregnancy in the past 12 months | All women of childbearing age and in union | Period | 12 | Infertility | 15.24 (14.7-15.4) |
| **EUROPEAN REGION** | | | | | | | | | | | |
| Bach et al. (2015) | Aarhus University Hospital, Skejby, Denmark | Cross-sectional | 1372 | Not reported | Retrospective Time-to-Pregnancy (TTP) Design | Nulliparous women with a TTP greater than 12 months or infertility treatment prior to the studied pregnancy | Nulliparous women who planned or partly planned their pregnancy and gave birth to a singleton | Period | 12 | Primary infertility | 21 |
| Guldbrandsen et al. (2014) | Denmark | Cohort | 73,107 | Not reported | Retrospective Time-to-Pregnancy (TTP) Design | Pregnant women with planned pregnancies and a TTP greater than 6/12 months | Pregnant women with a planned pregnancy | Period | 6 12 | Subfecundity | 32.0 16.0 |
| Hollegaard et al. (2007) | Odense University Hospital, Odense, Denmark | Cross-sectional | 2927 | 18 + | Retrospective Time-to-Pregnancy (TTP) Design | Pregnant women with a TTP greater than 12 months | Pregnant women | Period | 12 | Subfertility | 17.3 |
| Kjaer Pedersen et al (1994) Translation | Funen County, Denmark | Case-control | 247 | 18 - 49 | Undetermined | Women who failed to conceive after trying for more than 12 months | All women (controls) | Lifetime | 12 | Infertility | 12.0 (8.0 - 17.0) |
|  |  |  | 182 |  |  | Women who failed to conceive after trying for more than 12 months | Women who had attempted and/or achieved pregnancy (controls) | Lifetime | 12 | Infertility | 17.0 (12.0 - 23.0) |
| Hærvig et al (2018) | Copenhagen, Denmark | Cross-sectional | 2140 | 50 - 51 | Self-reported infertility measure (Direct) | Men who ever tried to achieve a pregnancy, without success during the first 12 months | All men | Lifetime | 12 | Infertility | 17.9 |
| Kirkegaard et al. (2014) | Aarhus University Hospital, Skejby, Denmark | Cross-sectional | 9507 | Not reported | Retrospective Time-to-Pregnancy (TTP) Design | Women with spontaneous planned pregnancy with TTP of 12/24 or more months | Women with spontaneous planned pregnancies | Period | 12 24 | Subfertility | 9.9 3.1 |
| Raatikainen et al. (2010) | Kuopio University Hospital, Kuopio, Finland | Cross-sectional | 17,114 | Not reported | Retrospective Time-to-Pregnancy (TTP) Design | Pregnant women who reported a TTP > 36 months | Pregnant women not using contraception at the time of pregnancy | Period | 36 | TTP >36 months | 2.2 |
| Terävä et al. (2008) | Helsinki, Vantaa, South-Western region, North Karelia region, Kuopio province, and Oulu province, Finland | Cross-sectional | 4371 | 25 - 64 | Self-reported infertility measure (Direct) | Women who experienced a time period when they had tried to become pregnant, but had not conceived or conception took more than 12 months | All women | Lifetime | 12 | Subfertility | 16.0 |
| Klemetti et al (2010) | Finland | Cross-sectional | Women: 1198 Men: 1093 | 30 - 44 | Self-reported infertility measure (Direct) | Women/men who made unsuccessful attempts to conceive a child over a period of 12 months or longer | All women/men | Lifetime | 12 | Infertility - women Men | 20.0 9.0 |
| Taponen et al. (2004) | Northern Finland | Case-control | 60 | 31 | Self-reported infertility measure (Direct) | Women who reported that Infertility has ever (earlier or at this moment) been a problem | Women not using oral contraceptives or IUD devices (control group) | Lifetime | No duration | Infertility | 10.0 |
| Slama et al. (2006) | France | Cross-sectional | 69 | 18-44 | Current Duration Design | Couples not yet pregnant after 12/24 months of unprotected intercourse (estimated) | Women at risk of pregnancy (18 to 44 years at interview, declared not to be pregnant, currently had a male partner, had had sexual intercourse within the last 2 months, did not use any birth control method (including sterilization of either partner), and had not given birth to a live- or stillborn baby in the 3 months before the interview) | Period | 12 24 | TTP > 12 months  TTP > 24 months | 34.0 (15.0 - 54.0) 16.0 (4.0 - 29.0) |
|  |  |  | 53 |  |  | Estimated proportion of couples not yet pregnant after 6/12/24 months who were trying to become pregnant | Women at risk of pregnancy (as defined above) and reported they were currently willing to become pregnant or that they had stopped contraception to become pregnant, even if they declared that they did not desire to become pregnant currently | Period | 6 12 24 | TTP > 6 months  TTP > 12 months  TTP > 24 months | 47.0 (28.0 - 66.0) 26.0(10.0 - 42.0) 10.0 (2.0 - 18.0) |
| Slama et al. (2012) | France | Cross-sectional | 867 (overall) 360 (primary) | 18-44 | Current Duration Design | Couples with no detected pregnancy during the first 6/12/24 months of unprotected intercourse (estimated) | Women at risk of pregnancy defined as not using any birth control method, had a male partner and had been sexually active in the previous month. | Period | 6   12   24 | Infertility  Primary infertility  Infertility  Primary infertility  Infertility  Primary infertility | 46.0 (36.0-35.0) 47.0 (26.0 - 68.0)  24.0 (19.0 - 30.0) 26.0 (15.0 - 36.0)  11.0 (8.0 - 14.0) 11.0 (7.0 - 16.0) |
|  |  |  |  |  |  | Couples who declared that they had stopped using birth control methods in order to obtain a pregnancy and had no detected pregnancy conceived during the first 6/12/24 months of unprotected intercourse (estimated) | Women at risk of pregnancy defined as not using any birth control method, had a male partner and had been sexually active in the previous month AND declared that they had stopped using birth control methods in order to obtain a pregnancy | Period | 6 12 24 | Infertility | 45.0 (34.0 - 55.0) 23.0 (18.0 - 28.0) 10.0 (8.0- 12.0) |
|  |  |  |  |  |  | Couples who declared that they had stopped using birth control methods in order to obtain a pregnancy and had no detected pregnancy conceived during the first 6/12/24 months of unprotected intercourse (estimated) | Women at risk of pregnancy defined as not using any birth control method, had a male partner and had been sexually active in the previous month AND declared that they had stopped using birth control methods in order to obtain a pregnancy AND who did not use infertility treatments over the CDUI period | Period | 6 12 24 | Infertility | 43.0 (34.0 - 53.0) 20.0 (16.0 - 25.0) 8.0 (6.0 - 10.0) |
| Eustache et al. (2004) | Cochin Hospital, Paris, France | Cross-sectional | 390 | Male partner: 20–45 | Retrospective Time-to-Pregnancy (TTP) Design | Pregnant couples who took more than 12 months to conceive and whose male partner did not provide a semen sample | Pregnant couples whose male partner did not provide a semen sample | Period | 12 | TTP > 12 months | 5.0 (3.2–7.3) |
| Muller et al. (2006) | Maternity units in Toulouse, Rennes, Lyons and Paris, France | Cross-sectional | Total: 894  Range:  178 - 273 | Men: 20 - 45 | Retrospective Time-to-Pregnancy (TTP) Design | Pregnant women with a TTP greater than 12 months | Pregnant women not using contraception at the start of pregnancy | Period | 12 | TTP > 12 mo: Range | 5.0 - 11.0 |
| Ajrouche et al (2014) | France | Case-control | 1167 | Not reported | Self-reported infertility measure (Direct) | Women who took more than a year to conceive the index child and/or needed to consult a doctor and/or needed for the mother or father to undergo fertility treatment | Women with a child (control group) | Period | 12 | Difficulty becoming pregnant | 18.0 |
| Kuppers-Chinnow and Karmaus (1997) Translation | Germany | Cross-sectional | 1216 | 25 - 45 | Retrospective Time-to-Pregnancy (TTP) Design | Women who have ever in their lifetime experienced a time of unprotected intercourse (TUI) (with or without the onset of pregnancy) of more than 12 months | Women who have ever been at risk of becoming pregnant (i.e., had unprotected sexual intercourse) | Lifetime | 12 | Subfecundity | 31.8 (29.4 - 34.6) |
| Datta et al. (2016) | Great Britain | Cross-sectional | Women:  Unweighted: 8066  Weighted: 7052   Men:  Unweighted: 5553  Weighted: 6811 | 16 - 74 | Self-reported infertility measure (Direct) | Women/men who ever had a time, lasting 12 months or longer, when they and their partner were trying for a pregnancy, but it didn’t happen | All women/men who reported having experience of heterosexual sex | Lifetime | 12 | Infertility - women Men | 12.5 (11.7–13.3) 10.1 (9.2–11.1) |
| Joffe (2000) | Great Britain | Cross-sectional | 1540 | 16-59 | Retrospective Time-to-Pregnancy (TTP) Design | Individuals with time to pregnancy greater than 12 months for first pregnancy | All individuals whose first pregnancy was a birth and was not due to a contraceptive failure | Period | 12 | TTP >12 months (Primary) | 15.0 |
| Gyorffy et al. (2014) | Hungary | Cross-sectional | 1069 | 24+ | Self-reported infertility measure (Direct) | Participants whose time-to-pregnancy had been longer than one year in case of any of their pregnancies. | All women (control group) | Lifetime | 12 | TTP > 12 months | 9.8 |
| Van der Avoort et al. (2003) | Boxmeer municipality, Netherlands | Cross-sectional | 243 | 25 - 40 | Self-reported infertility measure (Direct) | Male respondents who reported a lack of conception after at least 12 months of unprotected intercourse | All men | Period | 12 | Subfertility | 8.6 |
|  |  |  | 137 |  |  | Male respondents who reported a lack of conception after at least 12 months of unprotected intercourse | Men at risk for fertility problems | Period | 12 | Subfertility | 15.3 |
| Hoenderboom et al. (2020) | Amsterdam, Rotterdam and South Limburg, Netherlands | Cohort | 2377 | 16 - 39 | Retrospective Time-to-Pregnancy (TTP) Design | Women with an attempted time to first pregnancy of > 12 months | Women who had ever attempted to conceive | Period | 12 | Primary infertility (Time to first planned pregnancy) | 16.7 |
| Sundby and Schei (1996) | South-Trfindelag, Norway | Cross-sectional | 4034 | 40 - 42 | Self-reported infertility measure (Direct) | Women who tried to become pregnant for more than a year without succeeding | All Women | Lifetime | 12 | Infertility | 10.3 |
|  |  |  |  |  |  | Women who tried to become pregnant for more than a year without succeeding and had never given birth to a child | Married women not using contraception, postmenarchal and premenopausal | Lifetime | 12 | Permanent Infertility | 2.6 |
|  |  |  |  |  |  | Women who tried to become pregnant for more than a year without succeeding and had given birth to at least one child | Married women not using contraception, postmenarchal and premenopausal | Lifetime | 12 | Subfertility | 7.7 |
| Rostad et al. (2013) | North Trondelag County, Norway | Cross-sectional | 4951 | 50 - 59 | Self-reported infertility measure (Direct) | Women who have ever tried for more than a year to get pregnant regardless of any subsequent birth | All women | Lifetime | 12 | Infertility | 12.7 |
| Nguyen et al. (2007) | Norway | Cross-sectional | 26,303 | 18 - 39 | Retrospective Time-to-Pregnancy (TTP) Design | Couples who planned their pregnancy and that took more than 12 months to achieve pregnancy or received infertility treatment | Couples who planned their pregnancy | Period | 12 | Infertility | 12.0 |
| Magnus et al. (2021) | Norway | Cohort | 64,064 | 27 - 62 | Hybrid* Primary Retrospective Time-to-Pregnancy (TTP) Design  Secondary: Self-reported infertility measure (Direct) | Women with a planned pregnancy and a TTP > 12 or reported use of assisted reproductive technologies | Women who had a planned pregnancy | Period | 12 | Time to pregnancy | 12.3 |
| Soares et al. (2011) Translation | Porto, Portugal | Cross-sectional | 1540 | 18+ | Self-reported infertility measure (Direct) | Women who reported ever trying to get pregnant for more than a year without success | All women | Lifetime | 12 | Infertility | 11.9 (10.4-13.7) |
| Philippov et al. (1998) | Tomsk, Western Siberia, Russia | Cross-sectional | 2000 | 18 - 45 | Undetermined | Women who had not conceived after 12 months or more of unprotected intercourse | Married women | Lifetime | 12 | Infertility Primary infertility  Secondary infertility | 16.7 3.8 12.9 |
| Bhattacharya et al. (2009) | Grampian, Scotland | Cross-sectional | 4066 (12-month)  4049 (24-month) | 31-50 | Self-reported infertility measure (Direct) | Women who had unsuccessfully attempted conception for 12/24 months or longer | Women whose fertility had been tested | Lifetime | 12     24 | Infertility Primary infertility Secondary infertility Experienced both  Infertility Primary infertility Secondary infertility  Experienced both | 17.5 (16.3–18.6) 10.5 (9.5–11.4) 5.3 (4.7–6.0) 1.7 (1.3–2.0)  9.1 (8.2–10.0) 5.9 (5.2–6.6) 2.9 (2.4–3.4) 0.3 (0.2–0.5) |
|  |  |  |  |  |  | Women who had unsuccessfully attempted conception for 12/24 months or longer and/or had sought medical help with conception | Women whose fertility had been tested | Lifetime | 12     24 | Infertility Primary infertility Secondary infertility Experienced both  Infertility Primary infertility Secondary infertility  Experienced both | 19.3 (18.1–20.5) 9.8 (8.9–10.7) 7.0 (6.2–7.8) 2.5 (2.0–2.9)  11.8 (10.8–12.8) 5.7 (5.0–6.4) 5.2 (4.5–5.9) 0.9 (0.6–1.1) |
| Cabrera-Leon et al. (2015) | Huelva City, Spain | Cross-sectional | 443 | 30 - 49 | Retrospective Time-to-Pregnancy (TTP) Design | Women who did not achieve pregnancy after having had sexual intercourse with vaginal penetration and no contraception for one year or more. | All women | Lifetime | 12 | Infertility Huelva City Spain  Primary infertility Huelva City Spain  Secondary infertility Huelva City Spain | 17.79 17.58 (17.57–17.59)   6.14 6.12 (6.12–6.12)   11.64 11.33 (11.32–11.37) |
|  |  |  |  |  |  | Women with biological children who spent more than 6/12/24 months trying to become pregnant with any of their biological children | All women | Lifetime | 6   12   24 | Subfertility  Huelva City Spain  Huelva City Spain  Huelva City Spain | 21.00 19.98 (19.97–20.0)  11.62 11.21 (11.2–11.22)  4.59 4.36 (4.35–4.37) |
|  |  |  |  |  | Self-reported infertility measure (Direct) | Women who perceived having or having had difficulty in getting pregnant | All women | Lifetime | No duration | Subjective Infertility: Huelva City Spain | 9.41 8.22 (8.21–8.23) |
| Akre et al. (1999) | Sweden | Cross-sectional | 401,653 | 20 + | Retrospective Time-to-Pregnancy (TTP) Design | Primiparous women who did not become pregnant after more than one year | Primiparous women | Period | 12 | Primary subfertility | 8.3 |
| Wulff et al. (1997) | Sweden | Cross-sectional | 534 | 25-44 | Self-reported infertility measure (Direct) | Women who experienced a period of infertility (inability to conceive within 12 months of unprotected intercourse) at some point in life | All women | Lifetime | 12 | Infertility ever | 24.3 |
|  |  |  |  |  |  | Couples who were unable to get pregnant (again) after having tried for 12 months | All women | Period | 12 | Primary infertility Secondary infertility | 6.2 2.8 |
| Hallen (2011) | Sweden | Cross-sectional | 201 | 18 - 55 | Self-reported infertility measure (Direct) | Men reporting a period of 1 or more years of involuntary childlessness during the last five years | All men (control group) | Period | 12 | Involuntary childlessness | 7.0 (3.4–10.5) |
| Björvang et al (2020) | Värmland, Sweden | Cross-sectional | 818 | Not  reported | Retrospective Time-to-Pregnancy (TTP) Design | Women with planned pregnancies who had a TTP greater than 12 months | Pregnant women with planned pregnancies | Period | 12 | Infertility | 9.7 |
| Brunetti et al. (1994)  Translation | Martigny, Valais, Switzerland | Cross-sectional | 216 | 29 | Retrospective Time-to-Pregnancy (TTP) Design | Women exposed to fertilisation (i.e. women wishing to have children, with a stable partner and a regular sex life) for 24 or more months who have never conceived by the age of 29 | Women exposed to fertilisation before their 28th birthday (i.e. women wishing to have children, with a stable partner, a regular sex life, and presence of 24 months of unprotectedness) | Lifetime | 24 | Primary infertility (unresolved) | 2.8 |
|  |  |  | 212 |  |  | Women aged 29 who waited at least once for more than a year before obtaining a conception | Women who have conceived | Lifetime | 12 | Unintentionally delayed motherhood (hypofertility; resolved subfertility) | 10.0 |
| Gokler et al. (2014) | Mahmudiye, Turkey | Cross-sectional | 570 | 18 - 49 | Self-reported infertility measure (Direct) | Women who have the inability to become pregnant despite regular sexual intercourse during the last year | All married women | Period | 12 | Infertility Primary (% of total) Secondary (% of total) | 12.8 38.4 61.6 |
| Sarac and Koc (2018) | Turkey | Cross-sectional | 5947 | 15 - 49 | Constructed infertility measure (Indirect) | Women who have been married for at least five years, have not used any birth control methods during that time, and have not given birth | Women who have been married for at least five years | Period | 60 | Primary infertility | 1.8 |
|  |  |  | 6835 |  |  | Women who have been married for at least one year, have not used any contraception during the last year and who have not become pregnant in the last year | Women who have been married for at least one year | Period | 12 | Infertility | 8.1 |
|  |  |  | 5860 | 18 - 44 |  | Women who were at risk of pregnancy in the first 12 months of the total 5-year period | Women who are at risk of exposure to pregnancy | Period | 12 | Infertility | 8.6 |
| Albayrak and Günay (2007) | Provincial centre of Kayseri province, Turkey | Cross-sectional | 2400 | 15 - 49 | Undetermined | Women who had never been able to conceive, although they had been married at least 12 months, were living with their husband and had a desire for a baby | Married women | Period | 12 | Primary infertility (Childless women) | 6.3 |
| Gunnell and Ewings (1994) | Somerset, UK | Cross-sectional | 2377 | 36 - 50 | Retrospective Time-to-Pregnancy (TTP) Design | Women who failed to become pregnant after 12/24 months of regular unprotected intercourse | All women | Lifetime | 12    24 | Infertility Primary infertility Secondary infertility  Infertility Primary infertility Secondary infertility | 26.4 (24.6 - 28.2) 16.1 (14.6 - 17.6) 15.8 (14.3 - 17.3)  12.9 7.4 6.6 |
|  |  |  |  |  |  | Women with primary unresolved infertility and women who became pregnant but remained involuntarily childless | All women | Lifetime | 12 | Involuntary Childlessness | 3.0 |
| Buckett and Bentick (1997) | Shropshire, UK | Cross-sectional | 728 | 45 - 55 | Retrospective Time-to-Pregnancy (TTP) Design | Women who tried to conceive for more than 12/24 months | All women | Lifetime | 12    24 | Infertility  Primary infertility Secondary infertility  Infertility | 17.3 (14.6 - 20.0) 10.6 (8.4-12.8) 6.7 (4.9 - 8.5)  12 (9.6 - 14.4) |
|  |  |  |  |  |  | Women with primary or secondary infertility who never conceived | All women | Lifetime | 12 | Unresolved involuntary infertility | 4.3 (2.8 - 5.8) |
| Oakley et al. (2010) | United Kingdom | Cross-sectional | 7702 | 18-55 | Self-reported infertility measure (Direct) | Women who reported having problems getting pregnant | Women who had become pregnant or ever tried to get pregnant | Lifetime | No duration | Self-reported infertility | 19.5 (18.6-20.4) |
|  |  |  |  |  |  | Women with at least 1 planned pregnancy with a TTP > 12 months | Women who had become pregnant or ever tried to get pregnant and reported the pregnancy was planned | Lifetime | 12 | TTP > 12 months | 16.0 (15.1-16.9) |
|  |  |  | 6584 | 40 - 55 |  | Women with no pregnancy despite trying | Women who had become pregnant or ever tried to get pregnant | Lifetime | No duration | Primary unresolved infertility | 2.4 (2.0-2.8) |
|  |  |  |  |  |  | Women with no live birth despite trying | Women who had become pregnant or ever tried to get pregnant | Lifetime | No duration | Primary unresolved infertility | 4.3 (3.8-4.8) |
| Bolumar et al. (1997) | Denmark, Germany, Italy, Poland, and Spain | Cross-sectional | 3187 | 25 - 44 | Retrospective Time-to-Pregnancy (TTP) Design | Women who had planned their pregnancies and reported a TTP greater than 12 months for their first pregnancy | Women who had stopped using birth control and had planned their pregnancy | Period | 12 | Primary infertility | 12.0 |
| Karmaus and Juul (1999) | Denmark, Germany, Italy, Poland, and Spain | Cross-sectional | 932 | 25 - 44 | Retrospective Time-to-Pregnancy (TTP) Design | Women with a time of unprotected intercourse (TUI) greater than 12/24 months for first TUI with a starting date less than 5 years before the interview | Time of unprotected intercourse (TUI) for first TUI with a starting date less than 5 years before the interview, among women at risk | Period | 12    24 | Primary Subfecundity:  Total Range   Total Range | 23.4 14.8 - 33.3  17.6 10.1 - 26.8 |
|  |  |  |  |  |  | Women planning their pregnancies with a time of unprotected intercourse (TUI) greater than 12/24 months for first TUI with a starting date less than 5 years before the interview | Time of unprotected intercourse (TUI) for first TUI with a starting date less than 5 years before the interview, among women at risk and planning pregnancy | Period | 12    24 | Primary Subfecundity:  Total Range   Total Range | 18.7 12.1 - 30.2  13.6 8.1 - 24.1 |
| Toft et al. (2005) | Warsaw, Poland; Kharkiv, Ukraine, multiple municipalities, Greenland | Cross-sectional | Warsaw: 376  Kharkiv: 307  Greenland: 520 | 18+ (Greenland)   Not reported for other sites | Retrospective Time-to-Pregnancy (TTP) Design | Married women with a TTP greater than 12 months | Married women not using contraception | Period | 12 | TTP > 12 months Warsaw Kharkiv Greenland | 19.0 27.0 15.0 |
| Jensen et al. (2001) | Copenhagen, Denmark; Paris, France; Edinburgh, Scotland; Turku, Finland | Cross-sectional | Range:  191 - 302 | Male partner: 20–45 | Retrospective Time-to-Pregnancy (TTP) Design | Pregnant couples who took more than 6/12 months to conceive and whose male partner provided a semen sample | Pregnant couples whose male partner provided a semen sample | Period | 6 12 | TTP > 6/12 mo: Range | 16.9 - 20.9 7.5 - 10.1 |
| **SOUTH EAST ASIAN REGION** | | | | | | | | | | | |
| Zargar et al. (1997) | Kashmir valley, India | Cross-sectional | 10,063 | 15 - 44 | Self-reported infertility measure (Direct) | Couples who conceived more than one year after marriage or had not yet conceived at the time of the survey despite unprotected sexual intercourse for more than one year after marriage | Couples married for one year or more | Period | 12 | Primary Infertility | 15.07 |
|  |  |  |  |  |  | Couples who had not yet conceived at the time of the survey despite unprotected sexual intercourse for more than one year after marriage | Couples married for one year or more | Period | 12 | Unresolved Primary Infertility | 4.66 |
| Unisa (1999) | Raga Reddy district, Andhra Pradesh, India | Cross-sectional | Not reported | 20 - 49 | Constructed infertility measure (Indirect) | Women who have been married for three or more years without a live birth | Women who have been married for three or more years | Period | 36 | Childlessness (3 or more years) | 5.0 |
| Katole and Saoji (2019) | Central India | Cross-sectional | 570 | 15 - 49 | Self-reported infertility measure (Direct) | Women at risk of becoming pregnant (not pregnant, sexually active, not using contraception, and not lactating) who report trying unsuccessfully for a pregnancy for 2 years or more | All married women | Period | 24 | Primary infertility | 8.9 |
| Udgiri and Patil (2019) | Vijayapur, Karnataka, India | Cross-sectional | 693 (Rural) 419 (Urban) | 20 - 49 | Self-reported infertility measure (Direct) | Women at risk of becoming pregnant who report trying unsuccessfully for a pregnancy for 2 years or more | Women at risk of becoming pregnant | Period | 24 | Infertility  Rural  Urban  Primary infertility Rural  Urban  Secondary infertility  Rural  Urban | 7.6  8.8  5.3  5.7  2.3  3.1 |
| Singh and Shukla (2015) | Uttar Pradesh, India | Cross-sectional | 44,415 | 20 - 34 | Self-reported infertility measure (Direct) | Women who ever had a problem getting pregnant despite cohabitation and exposure to pregnancy for two or more years | All women married for at least two years | Lifetime | 24 | Infertility  Primary infertility | 10.7 8.4 |
|  |  |  |  |  |  | Women who never had a live birth up to the interview date, and reported problems conceiving for the first time (failure to conceive despite two years of cohabitation and exposure to pregnancy) | All women married for at least two years | Period | 24 | Primary infertility | 2.6 |
|  |  |  | 21,583 |  |  | Women who never had a live birth up to the interview date, and reported problems conceiving for the first time (failure to conceive despite two years of cohabitation and exposure to pregnancy) | Women married for at least two years and not using contraception | Period | 24 | Primary infertility | 2.3 |
| Purkayastha (2020) | India | Cross-sectional | 499, 627 | 15 - 49 | Constructed infertility measure (Indirect) | Currently married women who are married for five years or more, not currently pregnant, never used contraceptives, have no terminated pregnancies, and have zero children ever born | Currently married women | Period | 60 | Primary infertility | 1.79 |
| Chauhan et al. (2015) | Nashik district, Maharashtra State, India | Cross-sectional | 1167 | 15+ | Undetermined | Women who failed to conceive following a previous pregnancy or abortion despite cohabitation and exposure to pregnancy in absence of contraception for one or more years | Ever married women | Period | 12 | Secondary infertility | 1.7 |
| Samarakoon et al. (2007) | Colombo District, Sri Lanka | Cross-sectional | 2000 | Women: 15 - 48 Men: 17 - 53 | Undetermined | Women who have never conceived in spite of cohabitation and exposure to pregnancy for a period of 12 months | All married women | Period | 12 | Primary infertility | 4.05 (3.2 - 4.9) |
|  |  |  | 1907 |  |  | Women who have previously conceived but have been unable to conceive subsequently despite cohabitation and exposure to pregnancy for a period of 24 months (exposure to pregnancy was from the end of the period of lactation amenorrhea for women who breast fed the previous infant). | All married women who have had a pregnancy | Period | 24 | Secondary infertility | 16 (14.39 - 17.60) |
| **REGION OF THE AMERICAS** | | | | | | | | | | | |
| Bushnik et al. (2012) | Canada | Cross-sectional | 3,225,900 | 18-44 | Constructed infertility measure (Indirect) | Couples who did not become pregnant after exposure to the risk of conception during the previous 12 months | Couples not using any form of birth control within the past 12 months | Period | 12 | Infertility Primary infertility | 15.7 (14.2 - 17.4) 20.6 (17.8 - 23.7) |
|  |  |  | 3,176,900 |  |  | Couples who did not become pregnant after exposure to the risk of conception during the previous 12 months | Couples who did not use any form of birth control within the past 12 months and reported having sexual intercourse in the past 12 months | Period | 12 | Infertility Primary infertility | 14 (12.6 - 15.6) 18.7 (15.9 - 21.7) |
|  |  |  |  |  |  | Couples who did not become pregnant after exposure to the risk of conception during the previous 12 months | Couples who did not use any form of birth control within the past 12 months, reported having sexual intercourse in the past 12 months, and reported ever having tried to become pregnant with their current partner | Period | 12 | Infertility  Primary infertility | 11.5 (10.2 - 12.9) 10.2 (8.3 - 12.5) |
| Risch et al. (1994) | Ontario, Canada | Case-control | 564 | 35 - 79 | Self-reported infertility measure (Direct) | Women who ever had an interval of time when pregnancy was attempted without success | All women (control group) | Lifetime | No duration | Secondary infertility | 7.6 |
| Dulberg and Stephens (1993) | Canada | Cross-sectional | 1413 (12-month) 1350 (24-month) | 18 - 44 | Constructed infertility measure (Indirect) | Women and their husband/partner who did not use any contraceptive method (non-surgical or surgical) and was not pregnant during 12/24 months prior to the interview. | Women who had been married or cohabitating for at least 12/24 months prior to the survey | Period | 12 24 | Infertility | 8.5 (7.0 - 9.9) 7.0 (5.6 - 8.4) |
| Balakrishnan and Maxim (1993) | Ontario, Canada | Cross-sectional | 7765 | 16 - 49 | Self-reported infertility measure (Direct) | Women who believe she or her partner are incapable of having children | All women in union | Period | No duration | Perceived infertility Primary infertility Secondary infertility | 3.19 5.22 2.79 |
|  |  |  | 9267 |  | Constructed infertility measure (Indirect) | Women who reported that neither they nor their partner were sterilized, had not used any form of contraception in the year prior to the survey, and were not currently pregnant or post-partum mothers | All women in union | Period | 12 | Inferred infertility Primary infertility Secondary infertility | 6.66 11.23 5.31 |
|  |  |  | 7765 |  |  | Women classified as perceived infertile (direct question) and/or inferred infertile (indirect questions) | All women in union | Period | 12 or no duration | Aggregate infertility Primary infertility Secondary infertility | 7.74 11.68 6.72 |
| Fuentes and Devoto (1994) | Downtown Santiago, Chile | Cross-sectional | 365 | 15 - 45 | Self-reported infertility measure (Direct) | Women married for one year failing to achieve pregnancy after one or more years of unprotected sexual intercourse | Women at risk of pregnancy in their first year of marriage (i.e., having unprotected intercourse). | Period | 12 | Infertility (prevalence) | 10.14 (9.15 - 11.05) |
|  |  |  | 270 |  |  | Women married for eight years failing to achieve pregnancy after one or more years of unprotected sexual intercourse | Women at risk of pregnancy in their eighth year of marriage (i.e., having unprotected intercourse). | Period | 12 | Infertility (prevalence) Primary infertility Secondary infertility | 7.04 3.33 (2.74 - 3.72) 3.71 (3.28 - 4.14) |
|  |  |  | 474 |  |  | Women married for one year failing to achieve pregnancy after one or more years of unprotected sexual intercourse | Married women | Period | 12 | Infertility (frequency) | 7.8 |
|  |  |  |  |  |  | Women married for eight years failing to achieve pregnancy after one or more years of unprotected sexual intercourse | Married women | Period | 12 | Infertility (frequency) | 4.01 |
|  |  |  |  |  |  | Women suffering from infertility at some point in their lives disregarding whether they are currently infertile or not | Married women | Lifetime | 12 | Infertility | 25.74 (+/- 3.9) |
| Priestley (2012) | Jamaica | Cross-sectional | 8180 | 15 - 49 | Constructed infertility measure (Indirect) | Sexually experienced non-contracepting women who are non-surgically sterile, subfecund (women who think pregnancy is difficult), or have a long interval without contraception (more than 24 months without the use of contraception) | Sexually experienced women | Period | 24 | Impaired fecundity Primary Secondary | 31 12.2 36.1 |
| Jacobson et al. (2018) | Georgia, United States | Cross-sectional | 1014 | 22 - 45 | Retrospective Time-to-Pregnancy (TTP) Design | Women who did not get pregnant after 6/12/24 months of regular (>3 times per month) unprotected sex | Women who were at risk of becoming pregnant | Lifetime | 6 12 24 | Infertility | 42.6 35.3 23.5 |
|  |  |  |  |  |  | Women who did not get pregnant after 6/12/24 months of regular (>3 times per month) unprotected sex while actively trying to become pregnant | Women who were at risk of becoming pregnant | Lifetime | 6 12 24 | Infertility | 25.5 19.7 11.2 |
|  |  |  |  |  |  | Women who did not get pregnant after 12 months of regular (>3 times per month) unprotected intercourse for those <35 years old or after 6 months for those ≥35 years old | Women who were at risk of becoming pregnant | Lifetime | 12  (6 ≥35yrs) | Infertility | 35.9 |
|  |  |  |  |  |  | Women who did not get pregnant after 12 months of regular (>3 times per month) unprotected intercourse for those <35 years old or after 6 months for those ≥35 years old while actively trying to become pregnant | Women who were at risk of becoming pregnant | Lifetime | 12  (6 ≥35yrs) | Infertility | 20.5 |
| Crawford et al. (2015) | Florida, Massachusetts, and Michigan, United States | Cross-sectional | Florida: 1,285 MA: 1,302  Michigan: 3,360 | 18 - 50 | Self-reported infertility measure (Direct) | Those who ever tried to get pregnant for a year or longer and were unable to do so | All adults | Lifetime | 12 | Infertility  Florida Massachusetts Michigan | 9.7 (7.6–11.8) 6.0 (4.6–7.5) 4.2 (3.5–5.0) |
|  |  |  | Florida: 736  MA: 1246  Michigan: 2742 |  |  | Those who ever tried to get pregnant for a year or longer and were unable to do so | All adults who ever tried to get pregnant | Lifetime | 12 | Florida Massachusetts Michigan | 25.3  9.9  5.8 |
| Thoma et al. (2013) | United States | Cross-sectional | 277 | 15-44 | Current Duration Design | Women who want to become pregnant with a TTP greater than 12 months (estimated) | Women at risk of pregnancy at time of interview (not using a method of contraception nor pregnant but were sexually active at the time of interview AND responded "Yes" to the question ‘‘Is the reason you are not using a method of birth control now because you, yourself, want to become pregnant as soon as possible?’’) | Period | 12 | TTP > 12 months Primary | 15.5 (8.6 - 27.5) 24.30 (12.4-43.5) |
|  |  |  | 222 |  |  | Women who want to become pregnant with a TTP greater than 12 months (estimated) | Women at risk of pregnancy at time of interview (not using a method of contraception nor pregnant but were sexually active at the time of interview AND responded "Yes" to the question ‘‘Is the reason you are not using a method of birth control now because you, yourself, want to become pregnant as soon as possible?’’) AND did not report use of current infertility treatment | Period | 12 | TTP > 12 months Primary | 12.6 (7.6-21.4) 18.3 (11.0-30.9) |
|  |  |  | 3812 |  | Constructed infertility measure (Indirect) | Married or cohabiting respondents who had been in a continuous relationship for 12 months or more with no use of contraception, but sexually active every month for the past 12 months, and did not have a pregnancy | All married or cohabiting respondents | Period | 12 | Infertility Primary infertility  Secondary infertility | 7.0 (6.2 - 7.8) 13.2 (11.2 - 15.2) 5.3 |
|  |  |  | Not reported |  |  | Married or cohabiting respondents who had been in a continuous relationship for 12 months or more with no use of contraception, but sexually active every month for the past 12 months, and did not have a pregnancy AND did not report current use of infertility treatment | All married or cohabiting respondents who did not report current use of infertility treatment. | Period | 12 | Infertility Primary infertility | 6.6 (5.8-7.5) 11.9 (9.9-13.9) |
| Louis et al. (2013) | United States | Cross-sectional | 157 | 15-45 | Current Duration Design | Men who are in a relationship and trying to become pregnant with a TTP > 12 months (estimated) | Men at risk of pregnancy at time of interview (sexually active in the past year with a female partner and currently trying to get pregnant) | Period | 12 | Infertility  Primary infertility | 12.0 (7.0 - 23.2) 14.0 (6.0 - 25.6) |
|  |  |  | Not reported |  |  | Men who are in a relationship and trying to become pregnant with a TTP > 12 months (estimated) | Men at risk of pregnancy at time of interview (sexually active in the past year with a female partner and currently trying to get pregnant) and who reported not having sought infertility treatment during their current pregnancy attempt. | Period | 12 | Infertility | 9.4 (5.2-17.2) |
| Chandra et al. (2013) | United States | Cross-sectional | 61,755 (Weighted population size)  12,279 (unweighted sample size) | 15-44 | Hybrid*  Primary: Self-reported infertility measure (Direct)  Secondary: Constructed infertility measure (Indirect) | Women with impaired fecundity (i.e. non-surgically sterile (self or partner), subfecund (self or partner), and/or long interval (36 or more months) without conception) | All women | Period | No duration or 36 | Impaired fecundity Primary infertility Secondary infertility | 10.9 (SE = 0.4) 11.2 (SE = 0.7) 10.6 (SE = 0.6) |
|  |  |  | 25, 605 (Weighted population size)  3,971 (unweighted sample size) |  |  | Women with impaired fecundity (i.e. non-surgically sterile (self or partner), subfecund (self or partner), and/or long interval (36 or more months) without conception) | Married women | Period | No duration or 36 | Impaired fecundity  Primary infertility Secondary infertility | 12.1 (SE = 0.8) 21.2 (SE = 2.0) 9.9 (SE = 0.8) |
|  |  |  | 62,128 (Weighted population size) |  | Self-reported infertility measure (Direct) | Men who are nonsurgically sterile (self or partner) or subfertile | All men | Period | No duration | Infertility | 9.4 (SE = 0.5) |
|  |  |  | 5422 (unweighted sample size) |  | Constructed infertility measure (Indirect) | Women and their partner who, during the previous 12 months or longer, were continuously married, were sexually active each month, had not used contraception, and had not become pregnant. | Married women | Period | 12 | Infertility  Primary infertility Secondary infertility | 6.0 (SE = 0.5) 14.0 (SE = 1.6) 4.0 (SE = 0.5) |
| Boulet et al. (2016) | United States | Cross-sectional | 8691 | 18-50 | Self-reported infertility measure (Direct) | Women who reported difficulty becoming or staying pregnant | All women | Lifetime | No duration | Perceived infertility | 13.20 (11.3-15.2) |
| Nelson et al. (2011) | Philadelphia, Pennsylvania, United States | Cross-sectional | 291 | 35 - 47 | Self-reported infertility measure (Direct) | Women who ever tried to get pregnant for over one year without being able to | All women | Lifetime | 12 | Infertility | 20.0 |
| McQuillan et al. (2003) | Upper Midwest, United States | Cross-sectional | 580 | 25 - 50 | Self-reported infertility measure (Direct) | Women who have (a) tried for longer than 12 months to conceive any of their pregnancies, (b) sought medical help to conceive any of their pregnancies, (c) ever tried to get pregnant for more than 12 months without success, and/or (d) ever had regular unprotected intercourse for more than a year without pregnancy. | All women | Lifetime | 12 | Subfecundity (medically defined infertility) | 35.0 |
| Weiss et al. (1998) | Atlanta, Georgia; Seattle/Puget Sound, Washington; and central New Jersey, United States | Cross-sectional | 1989 | Seattle and New Jersey: 20 - 44   Atlanta: 20 - 54 | Self-reported infertility measure (Direct) | Women who report difficulty in either becoming pregnant or maintaining a pregnancy. | All women | Lifetime | No duration | Infertility Primary infertility | 19.0 31.0 |
| Merritt et al. (2013) | Massachusetts and New Hampshire, United States | Case-control | 2100 | Not reported | Self-reported infertility measure (Direct) | Women who had tried to become pregnant without success or had seen a doctor about having difficulties in getting pregnant or carrying a pregnancy to term. | All women (control group) | Lifetime | No duration | Infertility | 20.8 |
| Jacob et al. (2007) | Upper Midwest, United States | Cross-sectional | 580 | 25 - 50 | Self-reported infertility measure (Direct) | Women who experienced 12 months of regular unprotected intercourse without conception at some time in their lives | All women | Lifetime | 12 | Infertility | 28.0 |
| Gleason et al. (2020) | United States | Cohort | 1652 | 29 - 35 | Self-reported infertility measure (Direct) | Women who reported regular sexual intercourse over at least 12 months without the use of contraception and without conceiving a child and ever tried to get pregnant | Women who ever tried to get pregnant | Lifetime | 12 | Infertility | 24.1 |
| Anyalechi et al. (2019) | United States | Cross-sectional | 2628 | 18-49 | Self-reported infertility measure (Direct) | Women who had ever had sexual intercourse with a male partner and attempted to get pregnant for 12 months without becoming pregnant | Women who ever had intercourse with a male partner | Lifetime | 12 | Infertility | 13.80 (12.3-15.3) |
| Cairncross et al. (2020) | United States | Cross-sectional | 2809 | 42 - 52 | Self-reported infertility measure (Direct) | Women unable to achieve a clinical pregnancy for a period of > 12 months of trying to conceive or who used fertility medications for > 1 month | Women who had ever attempted to conceive | Lifetime | 12 or fertility medication for > 1 month | Infertility | 24.7 |
| **WESTERN PACIFIC REGION** | | | | | | | | | | | |
| Herbert et al. (2009a) | Australia | Cohort | 13,715 | 45 - 50 | Self-reported infertility measure (Direct) | Women who have tried unsuccessfully to get pregnant for 12 months or more, have been diagnosed as infertile by a doctor (self or partner), and/or had treatment for infertility in lifetime (self or partner) | Women born between 1946 - 1951 | Lifetime | 12 | Infertility | 11.0 |
| Herbert et al. (2009b) | Australia | Cohort | 1031 | 28 - 33 | Self-reported infertility measure (Direct) | Women who had tried to conceive for 12 or more months unsuccessfully | Women who had tried to conceive or had been pregnant | Lifetime | 12 | Infertility | 17.3 |
| Mena et al. (2020) | Australia | Cohort | 6130 | Time 1: 22 - 27  Final time: 37 - 42 | Self-reported infertility measure (Direct) | Women who reported ever having problems with infertility (tried unsuccessfully to get pregnant for 12 months or more) with a current or previous partner | Women who ever tried to get pregnant | Period | 12 | Infertility (Cumulative incidence) | 15.4 (14.5 - 16.4) |
| Damone et al. (2019) | Australia | Cross-sectional | 8612 | 28 - 33 | Self-reported infertility measure (Direct) | Couples who ever had problems with  fertility (tried unsuccessfully for 12 months or more to get pregnant) | All women in union (currently or previously) | Lifetime | 12 | Infertility | 11.1 |
| Zhou et al. (2018) | Zhejiang, Anhui, Fujian, Shandong, Beijing,  Hebei, Heilongjiang and Nei Mongol Provinces, China | Cross-sectional | 17,275 | 20 - 49 | Retrospective Time-to-Pregnancy (TTP) Design | Women who wanted to become pregnant in the previous year, who had unprotected sexual intercourse at least once a month, and who were trying to achieve pregnancy longer than 12 months | Women exposed to the risk of pregnancy (not using contraception and had not lived separated longer than three months) | Period | 12 | Infertility Primary infertility Secondary infertility | 15.5 9.5 6 |
|  |  |  | 10,742 |  |  | Women who wanted to become pregnant in the previous year, who had unprotected sexual intercourse at least once a month, and who were trying to achieve pregnancy longer than 12 months | Women attempting to become pregnant (not using contraception, had not lived separated longer than three months, and willing to become pregnant) | Period | 12 | Infertility Primary infertility Secondary infertility | 25 15.3 9.7 |
| Xingping et al. (2006) Translation | Shanxi Province, China | Cross-sectional | 5,325,844 | < 49 | Undetermined | Women who failed to achieve pregnancy after one year of regular sexual intercourse without contraception | Married women of childbearing age | Period | 12 | Infertility Primary (% of total) Secondary (% of total) | 1.57 82.4 17.6 |
| Zhang et al. (2014) | Beijing, China | Cross-sectional | 12,342 | 27 - 57 | Self-reported infertility measure (Direct) | Married couples who failed to achieve a clinical pregnancy after 12 months of regular unprotected sexual intercourse | All married couples who had regular unprotected intercourse for at least 12 months prior to the date of interview | Period | 12 | Infertility Primary infertility Secondary infertility | 4.2 3.1 1.1 |
| Yang et al. (2011) Translation | Xicheng, Fengtai, Shijingshan, Changping, Daxing, and Shunyi districts, and Yanqing County, Beijing, China | Cross-sectional | 5631 | 20 - 49 | Undetermined | Couples who had a desire to have children, had normal cohabitation for two years, had regular sexual intercourse, and were not pregnant without contraception | All couples | Period | 24 | Infertility  Primary (% of total) Secondary (% of total) | 1.72 58.76 41.24 |
| Wu et al. (2004) Translation | Zhenghe County, Fujian Province, China | Cross-sectional | 274 | Reproductive age | Undetermined | Married women who did not use contraception within two years after marriage and did not become pregnant | Married women | Period | 24 | Infertility | 1.1 |
| Cai et al. (2011) Translation | Xinjiang Uygur Autonomous Region, China | Cross-sectional | 1835 | 20 - 49 | Self-reported infertility measure (Direct) | Married women with an absence of pregnancy after 1 year of normal sexual intercourse without the use of contraception under the conditions of exposure to pregnancy | Married women | Period | 12 | Infertility  Primary infertility Secondary infertility | 15.2 7.5 7.7 |
| Zhang and Zhang (2013) Translation | Gansu province, China | Cross-sectional | 2187 | 20-49 | Undetermined | Couples who had a desire to have children, normal sexual activity for more than one year and have not used contraception, but still fail to conceive | Married women willing to have a child and not using contraception during a specified one-year period | Period | 12 | Primary Infertility Secondary infertility | 13.08 35.25 |
| Song (2013) | China | Cross-sectional | 3110 | 37 - 38 | Retrospective Time-to-Pregnancy (TTP) Design | Women with no child after 24/84 months of marriage | All married women | Period | 24 84 | Sterility | 14.24 1.67 |
| Wang et al. (2018) | Shangai, China | Cohort | 700 | 20 - 40 | Prospective Time-to-Pregnancy (TTP) Design | Couples in a committed relationship and planning to conceive with a TTP greater than 12 months | Couples in a committed relationship planning to conceive | Period | 12 | Infertility | 28.0 |
| Yang et al. (2017) | Sandu Shui Autonomous County, Guizhou Province, China | Cross-sectional | 7025 | 18 - 49 | Retrospective Time-to-Pregnancy (TTP) Design | Couples whose waiting time to pregnancy was 12 or more months for the first pregnancy or couples who had never been pregnant and time trying to conceive was 12 or more months | Couples who had been married for more than 12 months | Period | 12 | Infertility | 11.4 |
| Huang and Tang (2013) Translation | Guangdong Province, China | Cross-sectional | 18,893 | Women: 18 - 49 | Undetermined | Married couples who had regular sexual intercourse and did not take contraceptive measures, but had not conceived after cohabitation for more than 12 months | Couples married in 2007 who did not use contraceptive measures within one year after marriage | Period | 12 | Infertility | 13.3 |
| Meng et al. (2015) | Xiyang County and Shouyang County, Shanxi Province, China | Cohort | 1627 (12-month) 936 (24-month) | Not reported | Prospective Time-to-Pregnancy (TTP) Design | Newly married couples who failed to achieve a clinical pregnancy after 12/24 months or more of regular unprotected sexual intercourse | Newly married couples exposed to the risk of pregnancy | Period | 12    24 | Infertility Primary infertility Secondary infertility   Infertility Primary infertility Secondary infertility | 13.6 (11.9 - 15.3) 14.0 (12.2 - 15.8) 11.2 (7.2 - 15.2)  8.5 (6.7 - 10.3) 8.7 (6.7 - 10.7) 7.9 (3.6 - 12.2) |
| Hu et al. (2020) | Shanghai, China | Cohort | 820 | 24-46 | Prospective Time-to-Pregnancy (TTP) Design | Couples who engaged in regular unprotected intercourse and had a TTP greater than 12 months | Couples who engaged in regular unprotected intercourse and achieved pregnancy during follow-up | Period | 12 | Infertility | 26.2 |
| He et al. (2020) | China | Cross-sectional | 12,364 | 18 - 49 | Undetermined | Women who failed to achieve a clinical pregnancy after one year or more of unprotected sexual intercourse, despite having a desire to get pregnant | All married and cohabitating women | Lifetime | 12 | Infertility | 10.11 |
|  |  |  | 2486 |  |  | Women who failed to achieve a clinical pregnancy after one year or more of unprotected sexual intercourse, despite having a desire to get pregnant | Married and cohabitating women not using contraception | Lifetime | 12 | Infertility | 20.92 |
| Chen et al. (2015) | Sandu Shui Autonomous County, Guizhou province, China | Cross-sectional | 6906 | > 21 | Retrospective Time-to-Pregnancy (TTP) Design | Couples with TTP greater than or equal to12 months or being unable to conceive after trying for at least 12 months for their first pregnancy | Married couples who ever tried for pregnancy | Period | 12 | Primary Infertility | 11.97 |
| Righarts et al. (2015) | Otago and Southland, New Zealand | Cross-sectional | 974 | 25 - 50 | Hybrid*  Primary: Self-reported infertility measure (Direct)  Secondary: Constructed infertility measure (Indirect) | Women who ever tried to conceive for 12/24 months or more | All women who had ever conceived or had tried to conceive | Lifetime | 12 24 | Infertility | 21.7 (19.1 - 24.2) 12.8 (10.7 - 15.2) |
|  |  |  |  |  |  | Women who ever tried to conceive for 12 months or more and/or sought help to conceive | All women who had ever conceived or had tried to conceive | Lifetime | 12 | Infertility | 25.3 (22.6 - 28.1) |
|  |  |  | 476 |  |  | Women ages 40 years or more with no previous births, whose infertility was not resolved with a live birth | Women ages 40 years or more who had ever tried to become or had been pregnant. | Lifetime | 12 | Primary unresolved infertility | 1.9 (0.9 - 3.6) |
| van Roode et al. (2015) | Dunedin, New Zealand | Cohort | Men: 386  Women: 396 | 32 and 38 | Self-reported infertility measure (Direct) | Men/women who, with a partner, had ever tried for 12 months or more to get pregnant without success | Men/women who ever reported or attempted pregnancy | Lifetime | 12 | Infertility - men women | 17.9 (14.2 - 22.1) 25.0 (20.8 - 29.6) |
|  |  |  |  |  |  | Men/women who, with a partner, had ever tried for 12 months or more to get pregnant without success OR who sought medical help to get pregnant | Men/women who ever reported or attempted pregnancy | Lifetime | 12 | Infertility - men  women | 21.8 (17.7-26.2) 26.0 (21.8-30.6) |
|  |  |  | Not reported |  |  | Men/women who, with a partner, had ever tried for 12 months or more to get pregnant without success OR who sought medical help to get pregnant | All men/women | Lifetime | 12 | Infertility - men  women | 18.2 (14.8 - 22.1) 22.5 (18.7 - 26.6) |
| Righarts et al. (2021) | New Zealand | Cross-sectional | Men: 3744 Women: 5222 | 16 - 74 | Self-reported infertility measure (Direct) | Men/women who ever had a time, lasting 12 months or longer, when they or a partner were trying for a pregnancy but it didn't happen | All men/women who had heterosexual intercourse | Lifetime | 12 | Infertility- men women | 8.2 (7.1-9.4) 12.5 (11.3-13.8) |
|  |  |  | Fertility-tested women: 3792 |  |  | Men/women who ever had a time, lasting 12 months or longer, when they or a partner were trying for a pregnancy but it didn't happen | Fertility-tested women (ever conceived or tried unsuccessfully to conceive for 12 months or longer) | Lifetime | 12 | Infertility | 15.4 (14.0-16.9) |
|  |  |  | Men: 3744 Women: 5222 |  |  | Men/women who ever had a time, lasting 12 months or longer, when they or a partner were trying for a pregnancy but it didn't happen OR they believed they or their partner were infertile. | All men/women who had heterosexual intercourse | Lifetime | 12 and/or no duration (self-perceived) | Infertility- men women | 11.4 (10.1-12.8) 13.4 (12.2-14.7) |
|  |  |  | Fertility-tested women: 3792 |  |  | Men/women who ever had a time, lasting 12 months or longer, when they or a partner were trying for a pregnancy but it didn't happen OR they believed they or their partner were infertile. | Fertility-tested women (ever conceived or tried unsuccessfully to conceive for 12 months or longer) | Lifetime | 12 and/or no duration (self-perceived) | Infertility | 16.3 (14.8-17.8) |
| Kreisel et al. (2020) | Palau | Cross-sectional | 315 | >17 | Self-reported infertility measure (Direct) | Women who have tried unsuccessfully to become pregnant for 12 or more months | Women who reported ever trying to become pregnant | Lifetime | 12 | Infertility | 39.7 (34.2 - 45.3) |
| Passey et al (1998) | Asaro Valley, Papua New Guinea | Cross-sectional | 201 | 15 - 45 | Self-reported infertility measure (Direct) | Women who reported that they wanted more children, were trying to conceive, and had had unprotected intercourse for 2 or more years | All women | Period | 24 | Infertility | 29.6 |
| **MULTIPLE REGIONS** | | | | | | | | | | | |
| Rutstein and Shah (2004) | Multiple | Cross-sectional | Less Developed Countries (China excluded): 939,796.7 | 25 - 49 | Constructed infertility measure (Indirect) | Women who have been married for the past five years, who ever had sexual intercourse, who did not use contraception during the past five years, and who did not have any births in the past five years | Ever-married women | Period | 60 | Infertility Range   Primary infertility Range   Secondary infertility Range | 25.7 16.0 - 30.0  2.5 1.5 - 2.8  23.8 13.6 - 28.2 |
| Keiding et al. (2021) | Benin, Nigeria, Senegal, Tanzania, Indonesia, Philippines, Dominican Republic, Colombia | Cross-sectional | Range: 211 - 1,183 | 18 - 44 | Current Duration Design | Nulliparous women not yet pregnant by 12 months (estimated) *Initiation of attempt estimated by date of cohabitation with partner | Women at risk of conception at the time of the survey (18 and 44 years, only one partner, currently married or living with a partner, had sex in the last 4 weeks, never had a live birth, menstruating and not currently pregnant, not menopausal, had not had a hysterectomy, and was not contracepting at the time of interview) | Period | 12 | Primary Infertility: Range | 24.0 - 85.0 |
| Mascarenhas et al. (2012a) | Global | Cross-sectional | Range: 337 - 62,785 | 20-44,  > 30 (countries with data on women in union only) | Constructed infertility measure (Indirect) | Women who desire a child and have been in a union for at least five years, during which they have not used any contraceptives, and who have not had a live birth | Women in both infertile and fertile unions, where women in a fertile union have successfully had at least one live birth and have been in the union for at least five years at the time of the survey | Period | 60 | Primary infertility: World Range | 1.9 (1.8 - 2.2) 0.8 - 4.0 |
|  |  |  |  |  |  | Women who desire a child and have been in a union for at least five years, during which they have not used any contraceptives, and who have not had a live birth | All women (calculated as the product of the prevalence of infertility among child-seeking women and the proportion who are exposed to the risk of pregnancy) | Period | 60 | Primary infertility: World Range | 1.5 (0.3 - 1.7) 0.5 - 3.0 |
|  |  |  | China excluded |  |  | Women who desire a child and have been in a union for at least five years since their last birth, during which they have not used any contraceptives, and who have not had another live birth | Women in both infertile and fertile unions, where women in a fertile union have successfully had at least one live birth and, at the time of the survey, have been in the union for at least five years following their first birth | Period | 60 | Secondary infertility:  World  Range | 10.2 (9.2 - 11.4) 3.8 - 22.2 |
|  |  |  |  |  |  | Women who desire a child and have been in a union for at least five years since their last birth, during which they have not used any contraceptives, and who have not had another live birth. | All women (calculated as the product of the prevalence of infertility among child-seeking women and the proportion who are exposed to the risk of pregnancy) | Period | 60 | Secondary infertility:  World  Range | 2.9 (2.6 - 3.2) 0.8 - 10.5 |
| Mascarenhas et al. (2012b) | 26 countries | Cross-sectional | Range: 532 - 71,095 (primary)  Range: 190 - 22,740 (secondary) | 20 - 49 | Constructed infertility measure (Indirect) | Women that have been in a union for at least five years (secondary infertility: since the partner's last live birth) without a live birth, during which neither partner used contraception, and where the female partner expresses a desire for a(nother) child.  . | Women in union for at least five years (secondary infertility: following their first live birth) at the time of the survey | Period | 60 | Primary: Range Secondary: Range | 0.8 - 3.4 8.7 - 32.6 |
| Taylor et al. (1999) | Manchester, UK and Melbourne, Australia | Cross-sectional | Melbourne: 929  Manchester: 960 | Not reported | Retrospective Time-to-Pregnancy (TTP) Design | Women who failed to conceive current pregnancy within 12 months of unprotected intercourse | Pregnant women | Period | 12 | Subfecundity Melbourne Manchester | 20.2 14.5 |

* Hybrid approach includes studies that combined two approaches to generate a single infertility estimate

# Supplementary Table SVII: Risk of bias scores for individual studies included in the review

|  | **Individual items used to assess risk of bias^1^** | | | | | | | |  |  |
| --- | --- | --- | --- | --- | --- | --- | --- | --- | --- | --- |
| **Authors (year)** | **Item #1: Sampling Frame** | **Item #2: Sampling methods** | **Item #3: Non-Response Bias** | **Item #4: Direct data collection** | **Item #5: Case Definition** | **Item #6: Study instrument** | **Item #7: Mode of data collection** | **Item #8: Numerator and denominator** | **Total number of items rated low risk** | **Overall risk of bias^2^** |
| Ahmadi Asr Badr et al. (2006) | Low | Low | High | Low | Low | High | Low | Low | 6 | **LOW** |
| Ajrouche et al (2014) | High | High | Low | Low | Low | Low | Low | High | 5 | **MODERATE** |
| Akhondi et al. (2019) | Low | Low | High | Low | Low | High | Low | High | 5 | **MODERATE** |
| Akre et al. (1999) | High | Low | Low | Low | Low | Low | Low | Low | 7 | **LOW** |
| Albayrak and Günay (2007) | High | Low | Low | Low | High | High | Low | Low | 5 | **MODERATE** |
| Anyalechi et al. (2019) | Low | High | Low | Low | Low | Low | Low | Low | 7 | **LOW** |
| Bach et al. (2015) | High | Low | High | Low | Low | Low | Low | Low | 6 | **LOW** |
| Balakrishnan and Maxim (1993) | Low | Low | Low | Low | Low | High | Low | Low | 7 | **LOW** |
| Barden-O'Fallon (2005) | Low | Low | High | Low | High | High | Low | Low | 5 | **MODERATE** |
| Bello et al. (2010) | Low | Low | High | Low | Low | Low | Low | High | 6 | **LOW** |
| Bernhard et al. (2000) | Low | Low | Low | Low | High | High | Low | Low | 6 | **LOW** |
| Bhattacharya et al. (2009) | Low | Low | High | Low | Low | Low | Low | Low | 8 | **LOW** |
| Björvang et al (2020) | High | Low | High | Low | Low | Low | Low | Low | 6 | **LOW** |
| Bolumar et al. (1997) | High | Low | High | Low | Low | Low | Low | Low | 6 | **LOW** |
| Boulet et al. (2016) | Low | Low | High | Low | High | High | Low | Low | 5 | **MODERATE** |
| Brunetti et al. (1994) Translation | High | Low | Low | Low | Low | Low | Low | Low | 7 | **LOW** |
| Buckett and Bentick (1997) | Low | Low | Low | Low | Low | Low | Low | Low | 8 | **LOW** |
| Bushnik et al. (2012) | Low | Low | Low | Low | Low | High | High | Low | 6 | **LOW** |
| Cabrera-Leon et al. (2015) | Low | Low | High | Low | Low | Low | Low | Low | 7 | **LOW** |
| Cai et al. (2011) Translation | High | Low | Low | Low | Low | High | Low | Low | 6 | **LOW** |
| Cairncross et al. (2020) | High | Low | High | Low | Low | High | Low | Low | 6 | **LOW** |
| Chandra et al. (2013) | Low | Low | Low | Low | Low | High | Low | Low | 7 | **LOW** |
| Chauhan et al. (2015) | High | High | High | Low | Low | High | Low | Low | 4 | **MODERATE** |
| Chen et al. (2015) | High | High | Low | Low | Low | Low | Low | High | 5 | **MODERATE** |
| Crawford et al. (2015) | High | Low | High | High | Low | Low | Low | Low | 5 | **MODERATE** |
| Damone et al. (2019) | Low | Low | High | Low | Low | Low | Low | Low | 7 | **LOW** |
| Datta et al. (2016) | Low | Low | High | Low | Low | Low | Low | Low | 7 | **LOW** |
| Dovom et al. (2014) | Low | Low | Low | Low | Low | High | Low | Low | 7 | **LOW** |
| Dulberg and Stephens (1993) | Low | Low | High | Low | Low | Low | Low | Low | 7 | **LOW** |
| Ekudayo (2020) | Low | Low | Low | Low | Low | High | Low | Low | 7 | **LOW** |
| Ericksen and Brunette (1996) | Low | Low | High | Low | Low | High | Low | Low | 6 | **LOW** |
| Esmaeilzadeh et al. (2012) | Low | Low | Low | Low | Low | High | Low | Low | 7 | **LOW** |
| Eustache et al. (2004) | High | Low | High | Low | Low | Low | Low | Low | 6 | **LOW** |
| Fledderjohann and Johnson (2016) | Low | Low | High | Low | Low | Low | Low | Low | 7 | **LOW** |
| Fledderjohann et al. (2017) | Low | Low | Low | Low | High | High | Low | Low | 6 | **LOW** |
| Fuentes and Devoto (1994) | Low | Low | Low | Low | Low | Low | Low | Low | 8 | **LOW** |
| Geelhoed et al. (2002) | Low | Low | Low | Low | Low | Low | Low | High | 7 | **LOW** |
| Gleason et al. (2020) | Low | Low | Low | Low | Low | Low | High | Low | 7 | **LOW** |
| Gokler et al. (2014) | Low | Low | High | Low | Low | High | Low | Low | 6 | **LOW** |
| Guldbrandsen et al. (2014) | High | High | High | Low | Low | Low | Low | Low | 5 | **MODERATE** |
| Gunnell and Ewings (1994) | Low | Low | Low | Low | Low | Low | Low | Low | 8 | **LOW** |
| Gyorffy et al. (2014) | Low | Low | High | Low | Low | Low | Low | Low | 7 | **LOW** |
| Hærvig et al (2018) | Low | Low | High | Low | Low | Low | Low | Low | 7 | **LOW** |
| Hallen (2011) | Low | High | High | Low | High | High | Low | Low | 4 | **MODERATE** |
| Hassan (1997) | High | Low | High | Low | Low | High | Low | Low | 5 | **MODERATE** |
| He et al. (2020) | Low | Low | High | Low | Low | High | Low | Low | 6 | **LOW** |
| Herbert et al. (2009a) | Low | Low | High | Low | Low | Low | Low | Low | 7 | **LOW** |
| Herbert et al. (2009b) | Low | Low | High | Low | Low | Low | Low | Low | 7 | **LOW** |
| Hoenderboom et al. (2020) | Low | Low | High | Low | Low | Low | Low | Low | 7 | **LOW** |
| Hollegaard et al. (2007) | High | Low | Low | Low | Low | High | Low | Low | 6 | **LOW** |
| Hosseini et al. (2012) | Low | Low | High | Low | Low | High | Low | Low | 6 | **LOW** |
| Hu et al. (2020) | High | Low | High | Low | Low | Low | Low | High | 5 | **MODERATE** |
| Huang and Tang (2013) Translation | Low | Low | Low | Low | Low | High | Low | Low | 7 | **LOW** |
| Jacob et al. (2007) | Low | Low | High | Low | Low | High | Low | Low | 6 | **LOW** |
| Jacobson et al. (2018) | Low | High | High | Low | Low | Low | Low | Low | 6 | **LOW** |
| Jensen et al. (2001) | High | Low | High | Low | Low | Low | Low | Low | 6 | **LOW** |
| Joffe (2000) | High | Low | High | Low | Low | Low | Low | Low | 6 | **LOW** |
| Karmaus and Juul (1999) | High | Low | High | Low | Low | Low | Low | Low | 6 | **LOW** |
| Katole and Saoji (2019) | High | Low | High | Low | Low | High | Low | Low | 5 | **MODERATE** |
| Kazemijaliseh et al. (2015) | Low | Low | High | Low | Low | Low | Low | Low | 7 | **LOW** |
| Keiding et al. (2021) | Low | Low | Low | Low | Low | High | Low | Low | 7 | **LOW** |
| Kirkegaard et al. (2014) | High | Low | High | Low | Low | Low | Low | Low | 6 | **LOW** |
| Kjaer Pedersen et al (1994) Translation | Low | Low | Low | Low | Low | High | Low | Low | 7 | **LOW** |
| Klemetti et al (2010) | Low | Low | Low | Low | Low | Low | Low | Low | 8 | **LOW** |
| Klouman et al. (2005) | Low | Low | Low | Low | High | High | Low | Low | 6 | **LOW** |
| Kreisel et al. (2020) | Low | Low | Low | Low | Low | Low | Low | Low | 8 | **LOW** |
| Kuppers- Chinnow and Karmaus (1997) Translation | Low | Low | High | Low | Low | Low | Low | Low | 7 | **LOW** |
| Larsen (2000) | Low | Low | Low | Low | Low | High | Low | Low | 7 | **LOW** |
| Larsen (2003) | Low | Low | Low | Low | Low | High | Low | Low | 7 | **LOW** |
| Larsen (2005) | Low | Low | Low | Low | Low | Low | Low | Low | 8 | **LOW** |
| Louis et al. (2013) | Low | Low | Low | Low | Low | High | Low | Low | 7 | **LOW** |
| Magnus et al. (2021) | High | Low | High | Low | Low | Low | Low | Low | 6 | **LOW** |
| Mascarenhas et al. (2012b) | Low | Low | Low | Low | Low | High | High | Low | 6 | **LOW** |
| Mascarenhas et al. (2012a) | Low | Low | Low | Low | Low | High | Low | Low | 7 | **LOW** |
| McQuillan et al. (2003) | Low | Low | High | Low | Low | Low | Low | Low | 7 | **LOW** |
| Mena et al. (2020) | Low | Low | High | Low | Low | Low | Low | Low | 7 | **LOW** |
| Meng et al. (2015) | High | Low | High | Low | Low | Low | High | Low | 5 | **MODERATE** |
| Merritt et al. (2013) | Low | High | High | Low | High | High | Low | Low | 4 | **MODERATE** |
| Miller-Fellows et al. (2017) | High | Low | Low | Low | Low | Low | Low | Low | 7 | **LOW** |
| Mirzaei et al. (2018) | Low | Low | Low | Low | Low | High | Low | High | 6 | **LOW** |
| Muller et al. (2006) | High | High | High | Low | Low | High | Low | Low | 4 | **MODERATE** |
| Nagaonle (2016) | Low | Low | Low | Low | Low | Low | Low | Low | 8 | **LOW** |
| Nasrabad et al. (2013) | High | High | High | High | Low | High | Low | High | 2 | **HIGH** |
| Nelson et al. (2011) | Low | Low | High | Low | Low | Low | Low | Low | 7 | **LOW** |
| Nguyen et al. (2007) | High | Low | High | Low | Low | Low | Low | Low | 6 | **LOW** |
| Oakley et al. (2010) | Low | Low | High | Low | Low | Low | Low | Low | 7 | **LOW** |
| Passey et al (1998) | Low | Low | Low | Low | Low | High | Low | Low | 7 | **LOW** |
| Philippov et al. (1998) | High | Low | High | Low | Low | High | Low | Low | 5 | **MODERATE** |
| Pick and Obermeyer (1996) | Low | Low | Low | Low | High | High | Low | Low | 6 | **LOW** |
| Polis et al. (2017) | Low | Low | Low | Low | Low | High | Low | Low | 7 | **LOW** |
| Priestley (2012) | Low | High | High | Low | High | High | Low | Low | 4 | **MODERATE** |
| Purkayastha (2020) | Low | Low | Low | Low | Low | High | Low | Low | 7 | **LOW** |
| Raatikainen et al. (2010) | High | Low | High | Low | High | High | High | High | 2 | **HIGH** |
| Rao et al. (2018) | Low | Low | Low | Low | Low | Low | Low | Low | 8 | **LOW** |
| Righarts et al. (2015) | High | Low | High | Low | Low | Low | High | Low | 5 | **MODERATE** |
| Righarts et al. (2021) | Low | Low | Low | Low | Low | Low | Low | Low | 8 | **LOW** |
| Risch et al. (1994) | Low | High | High | Low | High | High | Low | Low | 4 | **MODERATE** |
| Rostad et al. (2013) | High | Low | Low | Low | Low | Low | Low | Low | 6 | **LOW** |
| Rutstein and Shah (2004) | Low | Low | Low | Low | High | High | Low | Low | 6 | **LOW** |
| Safarinejad (2008) | Low | Low | High | Low | Low | Low | Low | Low | 7 | **LOW** |
| Samarakoon et al. (2007) | Low | Low | High | Low | Low | High | Low | Low | 6 | **LOW** |
| Sarac and Koc (2018) | Low | Low | Low | Low | Low | High | Low | High | 6 | **LOW** |
| Sharif (2020) | Low | Low | Low | Low | Low | High | Low | Low | 7 | **LOW** |
| Singh and Shukla (2015) | High | Low | High | Low | High | High | Low | Low | 4 | **MODERATE** |
| Slama et al. (2006) | Low | Low | Low | Low | Low | High | Low | Low | 7 | **LOW** |
| Slama et al. (2012) | Low | Low | Low | Low | Low | High | Low | Low | 7 | **LOW** |
| Soares et al. (2011) Translation | Low | Low | High | Low | Low | Low | Low | Low | 7 | **LOW** |
| Song (2013) | High | Low | High | Low | Low | High | Low | Low | 5 | **MODERATE** |
| Sundby and Schei (1996) | Low | Low | Low | Low | Low | Low | Low | Low | 8 | **LOW** |
| Sundby et al. (1998) | Low | Low | Low | High | Low | Low | Low | Low | 7 | **LOW** |
| Taponen et al. (2004) | Low | Low | High | Low | High | High | Low | Low | 5 | **MODERATE** |
| Taylor et al. (1999) | High | High | High | Low | Low | Low | Low | Low | 5 | **MODERATE** |
| Terävä et al. (2008) | Low | Low | Low | Low | Low | Low | Low | Low | 8 | **LOW** |
| Thoma et al. (2013) | Low | Low | Low | Low | Low | High | Low | Low | 7 | **LOW** |
| Toft et al. (2005) | High | Low | High | Low | Low | High | Low | Low | 5 | **MODERATE** |
| Udgiri and Patil (2019) | Low | Low | High | Low | Low | Low | Low | Low | 7 | **LOW** |
| Unisa (1999) | Low | Low | High | Low | Low | High | Low | High | 5 | **MODERATE** |
| Vahidi et al. (2009) | Low | Low | Low | Low | Low | Low | Low | Low | 8 | **LOW** |
| Van der Avoort et al. (2003) | Low | Low | High | High | Low | Low | Low | Low | 6 | **LOW** |
| van Roode et al. (2015) | Low | High | Low | Low | Low | Low | Low | Low | 7 | **LOW** |
| Walraven et al. (2001) | Low | Low | Low | Low | Low | Low | Low | Low | 8 | **LOW** |
| Wang et al. (2018) | High | Low | High | Low | Low | Low | Low | Low | 6 | **LOW** |
| Weiss et al. (1998) | Low | High | Low | Low | High | High | Low | Low | 5 | **MODERATE** |
| Woodall and Kramer (2018) | Low | Low | Low | Low | Low | High | Low | Low | 7 | **LOW** |
| Wu et al. (2004) Translation | Low | Low | High | Low | Low | High | Low | Low | 6 | **LOW** |
| Wulff et al. (1997) | Low | Low | High | Low | Low | High | Low | Low | 6 | **LOW** |
| Xingping et al. (2006) Translation | Low | Low | High | Low | Low | High | Low | Low | 6 | **LOW** |
| Yang et al. (2011) Translation | High | High | Low | Low | Low | High | Low | Low | 5 | **MODERATE** |
| Yang et al. (2017) | High | High | Low | Low | Low | Low | Low | Low | 6 | **LOW** |
| Zargar et al. (1997) | Low | Low | High | Low | Low | High | Low | Low | 6 | **LOW** |
| Zhang and Zhang (2013) Translation | Low | Low | Low | Low | Low | High | Low | Low | 7 | **LOW** |
| Zhang et al. (2014) | Low | Low | High | Low | Low | Low | Low | Low | 7 | **LOW** |
| Zhou et al. (2018) | Low | Low | High | Low | Low | Low | Low | Low | 7 | **LOW** |

^1^ Individual items used to assess risk of bias:

1. Was the sampling frame a true or close representation of the target population?
2. Was some form of random selection used to select the sample or was a census undertaken?
3. Was the likelihood of non-response bias minimal?
4. Were data collected directly from the subjects (as opposed to a proxy)?
5. Was an acceptable case definition used in the study?
6. Was the study instrument that measured the parameter of interest (e.g., prevalence of infertility) shown to have reliability and validity (if necessary)?
7. Was the same mode of data collection used for all subjects?
8. Were the numerator(s) and denominator(s) for the parameter of interest appropriate?

^2^ Overall summary score was divided into the following tertiles: 1) low risk of bias: 6 - 8 points, 2) moderate risk of bias: 3 - 5 points, and 3) high risk of bias: 0 - 2 points

# Supplementary Table SVIII: Results from sensitivity analysis

| **Criterion** | **Pooled lifetime infertility, %**  **(95% CI)** | **Pooled period infertility, %**  **(95% CI)** |
| --- | --- | --- |
| Overall estimates | 17.5 (15.0, 20.3) | 12.6 (10.7, 14.6) |
| **Sensitivity analysis criterion applied** |  |  |
| Linked studies replaced with minimum value | 14.5 (12.3, 17.1) | 11.7 (10.0, 13.7) |
| Limited to high quality studies (bias score >7) | 18.1 (15.7, 20.8) | 13.9 (10.5, 18.2) |
| General population studies only | 17.5 (15.0, 20.3) | 12.4 (9.9, 15.5) |
| Limited to studies in which standard errors could be obtained directly | 19.0 (16.4, 21.9) | 12.2 (10.2, 14.6) |
