# Supplementary material for: Infertility prevalence and the methods of estimation from 1990 to 2021: a systematic review and meta-analysis
Source: Hum Reprod Open. 2022 Nov 12;2022(4):hoac051. doi: 10.1093/hropen/hoac051 (PMC9725182; doi:10.1093/hropen/hoac051)
Supplement: hoac051_Supplementary_Figures [file hoac051_supplementary_figures.docx]

**Supplementary figures**

**Infertility prevalence and the methods of estimation from 1990 - 2021: a systematic review and meta-analysis**

*Carie M. Cox, Marie E. Thoma, Nedelina Tchangalova, Gitau Mburu, Marta J. Bornstein,*

*Courtney L. Johnson & James Kiarie*

**Table of contents**

[Supplementary figure S1: Funnel plot – studies reporting lifetime prevalence of infertility 2](#_Toc116647490)

[Supplementary figure S2: Funnel plot – studies reporting period prevalence of infertility 3](#_Toc116647491)

[Supplementary figure S3: Lifetime and period prevalence of 12-month infertility by methodologic approach 4](#_Toc116647492)

[Supplementary figure S4: Lifetime and period prevalence of 12-month infertility by country income level 5](#_Toc116647493)

[Supplementary figure S5: Lifetime and period prevalence of 12-month infertility by WHO region 6](#_Toc116647494)

[Supplementary figure S6: Forest plot of primary 12-month infertility prevalence 7](#_Toc116647495)

[Supplementary figure S7: Forest plot of secondary 12-month infertility prevalence 8](#_Toc116647496)

#


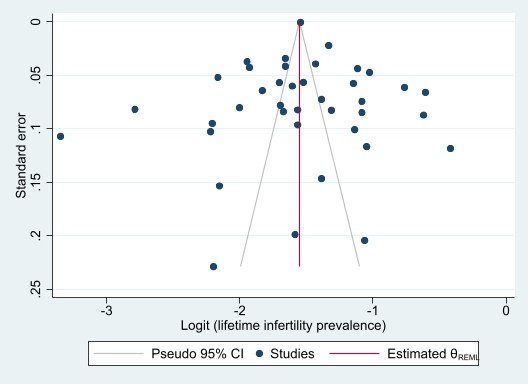


# Supplementary figure S1: Funnel plot – studies reporting lifetime prevalence of infertility


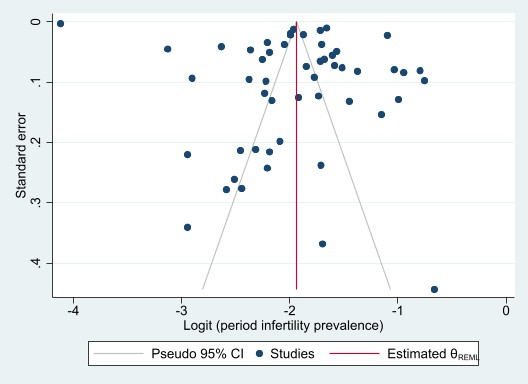


# Supplementary figure S2: Funnel plot – studies reporting period prevalence of infertility


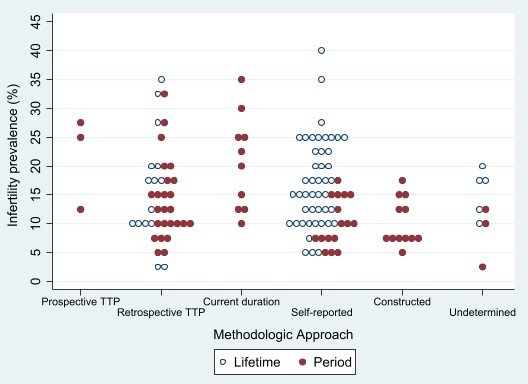


# Supplementary figure S3: Lifetime and period prevalence of 12-month infertility by methodologic approach

**
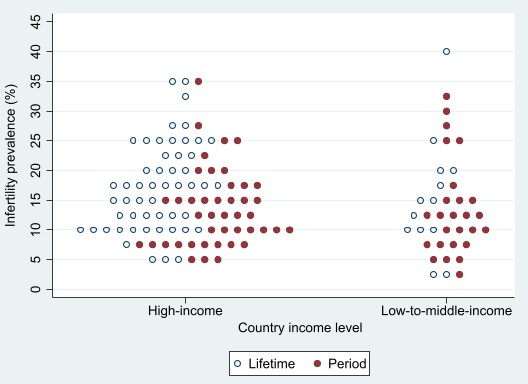
**

# Supplementary figure S4: Lifetime and period prevalence of 12-month infertility by country income level

**
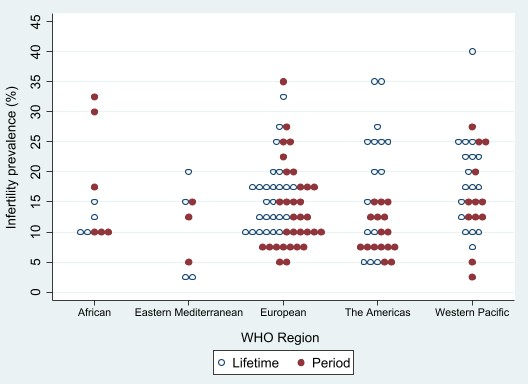
**

# Supplementary figure S5: Lifetime and period prevalence of 12-month infertility by WHO region


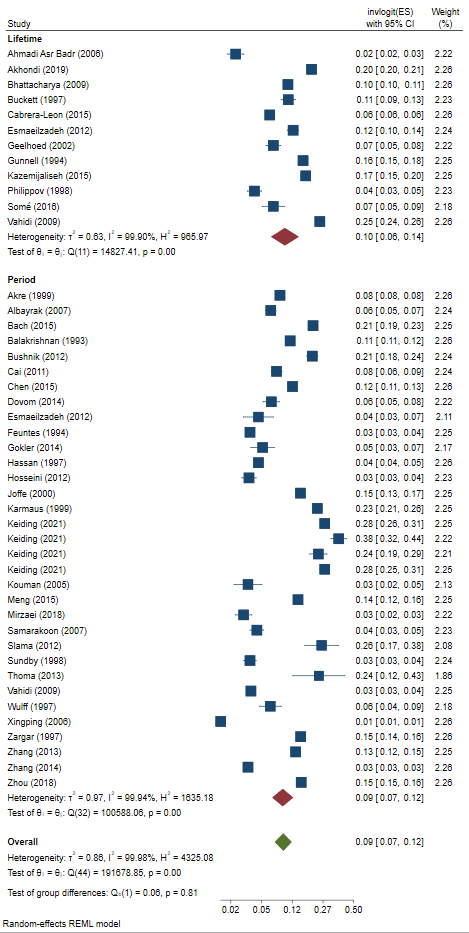


# Supplementary figure S6: Forest plot of primary 12-month infertility prevalence


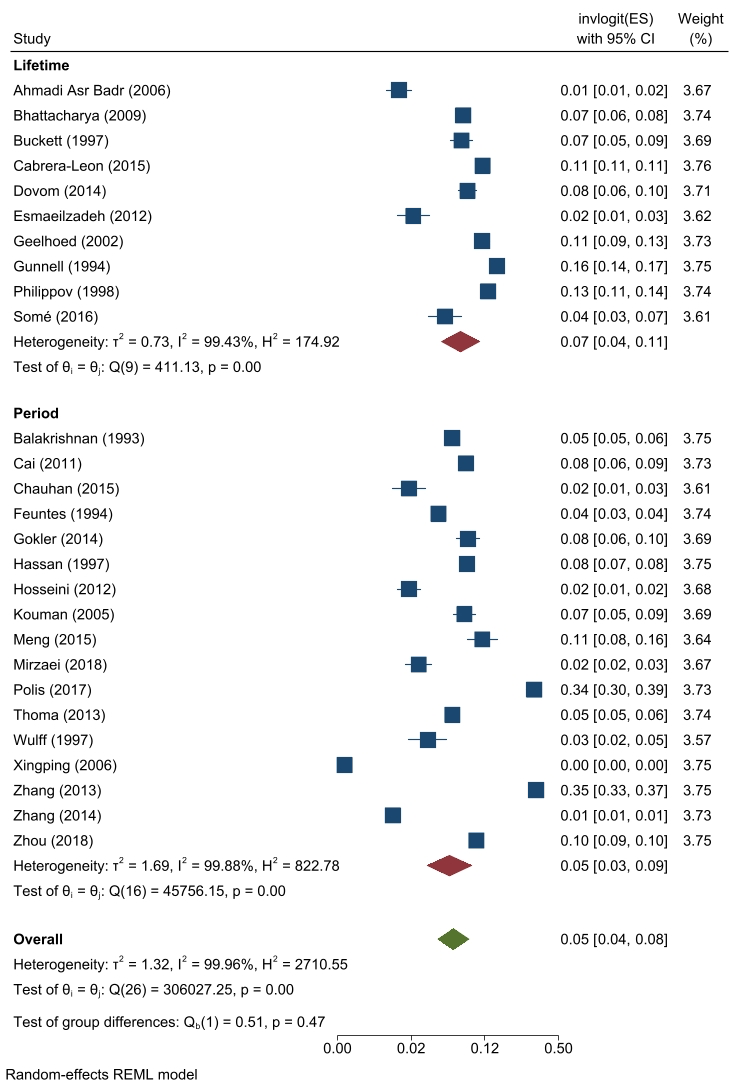


# Supplementary figure S7: Forest plot of secondary 12-month infertility prevalence
