# Supplementary material for: Infertility prevalence and the methods of estimation from 1990 to 2021: a systematic review and meta-analysis
Source: Hum Reprod Open. 2022 Nov 12;2022(4):hoac051. doi: 10.1093/hropen/hoac051 (PMC9725182; doi:10.1093/hropen/hoac051)
Supplement: hoac051_Supplementary_Files1_2 [file hoac051_supplementary_files1_2.docx]

# Supplementary File S1: Records with full text not located

Bettocchi C, Coker C, Parkinson M, Pryor J, Selvaggi F. Testicular cancer insitu: incidence and relation to infertility. In Boscia FM (ed) *Third Italian Conference on Andrology.* 1994. Monduzzi Editore, Bologna, pp. 369–371.

Guillén Pérez M, Candelario Madariaga M, Cruz Roja Z, Leonard Castillo A, Padrón Durán RS. [The prevalence of infertility and the importance of nursing work in this field]. *Rev Cubana Enferm* 1992;**8**:92–101.

Kinsella K, Koroleckyj-Cox T. Cross-national comparison of the prevalence and consequences of childlessness. *Gerontologist* 2003;**43**:414–415.

Konovalov OE. Sotsial’no-gigienicheskoe issledovanie épidemiologii pervichnogo i vtorichnogo zhenskogo besplodiia. *Problemy sotsial’noi gigieny i istoriia meditsiny* 1998;**5**:10–11.

Mohammad B. Prevalence of infertility in Sanandaj in 2002. *Scientific Journal of Kurdistan University of Medical Sciences* 2002;**7**:22–26.

Mohsen G, El-Awady M, Abdelazeem O. Prevalence of infertility in rural areas of Kafr El-Sheikh-Egypt: a community-based study. *J Egypt Public Health Assoc* 2001;**76**:469–486.

Rostami D, Rameazni T, Abedini M, Amirshekari G, Mehrabi Y. Prevalence of primary and secondary infertility among 18-49 years old Iranian women: a population-based study in four selected provinces. *Hakim Research Journal* 2014;**16**:294–301.

Simko J, Hollý I, Hudecová M, Zaviacic T, Holomán K. [Diagnosis of Chlamydia trachomatis using PCR in gynecology patients in Slovakia]. *Ceska Gynekol* 2002;**67**:376–379.

Snow RC, Okonofua FE, Kane T, Farley TMM, Pinol A. Prevalence and determinants of infertility in Ile-Ife, Nigeria. *Contracept Fertil Sex* 1995;**23**:544.

Sundby J. Infertility-causes, care and consequences: studies of frequency, risk factors, psychological consequences and health services for Norwegian women with reduced fertility or childlessness. 1994; University of Oslo, Department Group of Community Medicine.

Toychuev RM, D. S. Mirzakulov, Payzildaev T.r. [Prevalence of infertility in men living in conditions of environmental pollution with organochlorine pesticides]. *Gigiena i Sanitarii︠a︡ [Гигиена и Санитария]* 2015;**94**:97–99.

# Supplementary File S2: Studies included in the systematic review

Ahmadi Asr Badr Y, Madaen K, Haj Ebrahimi S, Ehsan Nejad AH, Koushavar H. Prevalence of infertility in Tabriz in 2004. *Urol J* 2006;**3**:87–91.

Ajrouche R, Rudant J, Orsi L, Petit A, Baruchel A, Nelken B, Pasquet M, Michel G, Bergeron C, Ducassou S, *et al.* Maternal reproductive history, fertility treatments and folic acid supplementation in the risk of childhood acute leukemia: the ESTELLE Study. *Cancer Causes Control* 2014;**25**:1283–1293.

Akhondi MM, Ranjbar F, Shirzad M, Ardakani ZB, Kamali K, Mohammad K. Practical difficulties in estimating the prevalence of primary infertility in Iran. *Int J Fertil Steril* 2019;**13**:113–117.

Akre O, Cnattingius S, Bergström R, Kvist U, Trichopoulos D, Ekbom A. Human fertility does not decline: evidence from Sweden. *Fertil Steril* 1999;**71**:1066–1069.

Albayrak E, Günay O. State and trait anxiety levels of childless women in Kayseri, Turkey. *Eur J Contracept Reprod Health Care* 2007;**12**:385–390.

Anyalechi GE, Hong J, Kreisel K, Torrone E, Boulet S, Gorwitz R, Kirkcaldy RD, Bernstein K. Self-reported infertility and associated pelvic inflammatory disease among women of reproductive age: National Health and Nutrition Examination Survey, United States, 2013-2016. *Sex Transm Dis* 2019;**46**:446–451.

Bach CC, Bech BH, Nohr EA, Olsen J, Matthiesen NB, Bossi R, Uldbjerg N, Bonefeld-Jørgensen EC, Henriksen TB. Serum perfluoroalkyl acids and time to pregnancy in nulliparous women. *Environ Res* 2015;**142**:535–541.

Balakrishnan TR, Fernando R. Infertility among Canadians: an analysis of data from the Canadian Fertility Survey (1984) and General Social Survey (1990). *The Prevalence of Infertility in Canada: Research Studies of the Royal Commission on New Reproductive Technologies* 1993;**6**:, p. 107–162. Minister of Supply and Services Canada Ottawa: Ottawa, Canada.

Barden-O’Fallon J. Associates of self-reported fertility status and infertility treatment-seeking in a rural district of Malawi. *Hum Reprod* 2005;**20**:2229–2236.

Bello B, Kielkowski D, Heederik D, Wilson K. Time-to-pregnancy and pregnancy outcomes in a South African population. *BMC Public Health* 2010;**10**:565–572.

Bernhard P, Makunde RW, Magnussen P, Lemnge MM. Genital manifestations and reproductive health in female residents of a wuchereria bancrofti-endemic area in Tanzania. *Trans R Soc Trop Med Hyg* 2000;**94**:409–412.

Bhattacharya S, Porter M, Amalraj E, Templeton A, Hamilton M, Lee AJ, Kurinczuk JJ. The epidemiology of infertility in the North East of Scotland. *Hum Reprod* 2009;**24**:3096–3107.

Björvang RD, Gennings C, Lin P-I, Hussein G, Kiviranta H, Rantakokko P, Ruokojärvi P, Lindh CH, Damdimopoulou P, Bornehag C-G. Persistent organic pollutants, pre-pregnancy use of combined oral contraceptives, age, and time-to-pregnancy in the SELMA cohort. *Environ Health* 2020;**19**:1–14.

Bolumar F, Olsen J, Boldsen J, European Study Group on nfertility Subfecundity. Smoking reduces fecundity: a European multicenter study on infertility and subfecundity. *Am J Epidemiol* 1996;**143**:578–587.

Boulet SL, Warner L, Adamski A, Smith RA, Burley K, Grigorescu V. Behavioral risk factor surveillance system state-added questions: leveraging an existing surveillance system to improve knowledge of women’s reproductive health. *J Womens Health* 2016;**25**:565–570.

Brunetti P, Morabia A, Campana A, Marcus-Steiff J. Biometrical study of reproduction conditions in the general-population: method and initial results of surveys carried out in chambery-grenoble and martigny. *Population* 1994;**49**:27–60.

Buckett W, Bentick B. The epidemiology of infertility in a rural population. *Acta Obstet Gynecol Scand* 1997;**76**:233–237.

Bushnik T, Cook JL, Yuzpe AA, Tough S, Collins J. Estimating the prevalence of infertility in Canada. *Hum Reprod* 2012;**27**:738–746.

Cabrera-Leon A, Lopez-Villaverde V, Rueda M, Moya-Garrido MN, Cabrera-León A. Calibrated prevalence of infertility in 30- to 49-year-old women according to different approaches: a cross-sectional population-based study. *Hum Reprod* 2015;**30**:2677–2685.

Cai X, Song R, Long M, Wang S, Ma Y, Li X, Ai H, Shan X, Fu L, Liu Y. [A cross-sectional study on the current status of female infertility in three counties of Xinjiang Uygur Autonomous Region]. *Zhonghua Yi Xue Za Zhi* 2011;**91**:3182–3185.

Cairncross ZF, Ahmed SB, Dumanski S, Nerenberg K, Metcalfe A. Infertility and the risk of cardiovascular disease: findings from the Study of Women’s Health Across the Nation (SWAN). *CJC Open* 2020;

Chandra A, Stephen EH. Infertility and impaired fecundity in the United States, 1982–2010: data from the National Survey of Family Growth. *Natl Health Stat Report* 2013;1–19.

Chauhan S, Kulkarni R, Agarwal D. Prevalence and factors associated with chronic obstetric morbidities in Nashik district, Maharashtra. *Indian J Med Res* 2015;**142**:479–488.

Chen J, Zhong C, Liang H, Yang Y, Zhang O, Gao E, Chen A, Yuan W, Wang J, Sun F, *et al.* The relationship between age at menarche and infertility among Chinese rural women. *Eur J Obstet Gynecol Reprod Biol* 2015;**194**:68–72.

Crawford S, Fussman C, Bailey M, Bernson D, Jamieson DJ, Murray-Jordan M, Kissin DM. Estimates of lifetime infertility from three states: the behavioral risk factor surveillance system. *J Womens Health* 2015;**24**:578–586.

Damone AL, Earnest A, Joham AE, Teede HJ, Moran LJ, Loxton D. Depression, anxiety and perceived stress in women with and without PCOS: a community-based study. *Psychol Med* 2019;**49**:1510–1520.

Datta J, Palmer MJ, Tanton C, Gibson LJ, Jones KG, Macdowall W, Glasier A, Sonnenberg P, Field N, Mercer CH, *et al.* Prevalence of infertility and help seeking among 15 000 women and men. *Hum Reprod* 2016;**31**:2108–2118.

Dovom MR, Tehrani FR, Abedini M, Amirshekari G, Hashemi S, Noroozzadeh M. A population-based study on infertility and its influencing factors in four selected provinces in Iran (2008-2010). *Iran J Reprod Med* 2014;**12**:561–566.

Dulberg CS, Stephens T. The prevalence of infertility in Canada, 1991–1992: analysis of three national surveys. *The Prevalence of Infertility in Canada: Research Studies of the Royal Commission on New Reproductive Technologies* 1993;**6**:, p. 61–106. Minister of Supply and Services Canada Ottawa: Ottawa, Canada.

Ekudayo O, Titilayo A, Anuodo O, Babalola O. Female genital cutting and infertility in marriage: a cross-sectional study among women in Nigeria. *Int J Educ Res* 2020;**8**:65–78.

Ericksen K, Brunette T. Patterns and predictors of infertility among African women: a cross-national survey of twenty-seven nations. *Soc Sci Med* 1996;**42**:209–220.

Esmaeilzadeh S, Delavar MA, Zeinalzadeh M, Mir M-RA. Epidemiology of infertility: a population-based study in Babol, Iran. *Women Health* 2012;**52**:744–754.

Eustache F, Auger J, Cabrol D, Jouannet P. Are volunteers delivering semen samples in fertility studies a biased population? *Hum Reprod* 2004;**19**:2831–2837.

Fledderjohann J, Johnson DR. Impaired fertility and perceived difficulties conceiving in Ghana: measurement problems and prospects. *J Biosoc Sci* 2016;**48**:431–456.

Fledderjohann J, Trinitapoli J, Billari F. Who seeks help? Responses to perceived fertility impairments in Malawi. *International Popular Conference* [Internet] 2017; International Union for the Scientific Study of Population (IUSSP). Available from: <https://iussp.confex.com/iussp/ipc2017/meetingapp.cgi/Paper/3525>.

Fuentes A, Devoto L. Infertility after 8 years of marriage: a pilot study. *Hum Reprod* 1994;**9**:273–278.

Geelhoed DW, Nayembil D, Asare K, Schagen van Leeuwen JH, Roosmalen J van. Infertility in rural Ghana. *Int J Gynaecol Obstet* 2002;**79**:137–142.

Gleason JL, Shenassa ED, Thoma ME. Stressful life events, the incidence of infertility, and the moderating effect of maternal responsiveness: a longitudinal study. *J Dev Orig Health Dis* 2020;1–9.

Gokler ME, Unsal A, Arslantas D. The prevalence of infertility and loneliness among women aged 18-49 years who are living in semi-rural areas in western Turkey. *Int J Fertil Steril* 2014;**8**:155–162.

Guldbrandsen K, Håkonsen LB, Ernst A, Toft G, Lyngsø J, Olsen J, Ramlau-Hansen CH. Age of menarche and time to pregnancy. *Hum Reprod* 2014;**29**:2058–2064.

Gunnell DJ, Ewings P. Infertility prevalence, needs assessment and purchasing. *J Public Health Med* 1994;**16**:29–35.

Győrffy Z, Dweik D, Girasek E. Reproductive health and burn-out among female physicians: nationwide, representative study from Hungary. *BMC Womens Health* 2014;**14**:121.

Hærvig KK, Kierkegaard L, Lund R, Bruunsgaard H, Osler M, Schmidt L. Is male factor infertility associated with midlife low-grade inflammation? A population based study. *Hum Fertil (Camb)* 2018;**21**:146–154.

Hallén M, Sandblom G, Nordin P, Gunnarsson U, Kvist U, Westerdahl J. Male infertility after mesh hernia repair: a prospective study. *Surgery* 2011;**149**:179–184.

Hassan KE. Prevalence of infertility and its impact on marital fertility, Egypt, 1993. 1997. Available from: <http://www.zohry.com/dwb/khassan/pub/infertility.pdf>.

He Y, Zheng D, Shang W, Wang X, Zhao S, Wei Z, Song X, Shi X, Zhu Y, Wang S, *et al.* Prevalence of oligomenorrhea among women of childbearing age in China: a large community-based study. *Womens Health* 2020;**16**:1-9.

Herbert D, Lucke J, Dobson A. Infertility, medical advice and treatment with fertility hormones and/or in vitro fertilisation: a population perspective from the Australian Longitudinal Study on Women’s Health. *Aust N Z J Public Health* 2009a;**33**:358–364.

Herbert DL, Lucke JC, Dobson AJ. Infertility in Australia circa 1980: an historical population perspective on the uptake of fertility treatment by Australian women born in 1946-51. *Aust N Z J Public Health* 2009b;**33**:507–514.

Hoenderboom BM, Bergen JEAM van, Dukers-Muijrers NHTM, Götz HM, Hoebe CJPA, Vries HJC de, Broek IVF van den, Vries F de, Land JA, Sande MAB van der, *et al.* Pregnancies and time to pregnancy in women with and without a previous chlamydia trachomatis infection. *Sex Transm Dis* 2020;**47**:739–747.

Hollegaard S, Vogel I, Thorsen P, Jensen IP, Mordhorst C-H, Jeune B. Chlamydia trachomatis C-complex serovars are a risk factor for preterm birth. *In Vivo* 2007;**21**:107–112.

Hosseini J, Emadedin M, Mokhtarpour H, Sorani M. Prevalence of primary and secondary infertility in four selected provinces in Iran, 2010-2011. *Iran J Obstet Gynecol Infertil* 2012;**15**:1–7.

Hu P, Cai C, Vinturache A, Hu Y, Gao Y, Zhang J, Lu M, Gu H, Qiao J, Tian Y, *et al.* Maternal preconception body mass index and time-to-pregnancy in Shanghai Women, China. *Women Health* 2020;**60**:1014–1023.

Huang J -t, Tang Y -g. Incidence of infertility and its influencing factors among married residents in Guangdong province. *China Public Health = Zhongguo Gong Gong Wei Sheng* 2013;**29**:0194–0197.

Jacob MC, McQuillan J, Greil AL. Psychological distress by type of fertility barrier. *Hum Reprod* 2007;**22**:885–894.

Jacobson MH, Chin HB, Mertens AC, Spencer JB, Fothergill A, Howards PP. “Research on infertility: definition makes a difference” Revisited. *Am J Epidemiol* 2018;**187**:337–346.

Jensen TK, Slama R, Ducot B, Suominen J, Cawood EHH, Andersen AG, Eustache F, Irvine S, Auger S, Jouannet P, *et al.* Regional differences in waiting time to pregnancy among fertile couples from four European cities. *Hum Reprod* 2001;**16**:2697–2704.

Joffe M. Time trends in biological fertility in Britain. *Lancet* 2000;**355**:1961–1965.

Karmaus W, Juul S. Infertility and subfecundity in population-based samples from Denmark, Germany, Italy, Poland and Spain. *Eur J Public Health* 1999;**9**:229–235.

Katole A, Saoji A. Prevalence of primary infertility and its associated risk factors in urban population of central India: a community-based cross-sectional study. *Indian J Community Med* 2019;**44**:337–341.

Kazemijaliseh H, Ramezani Tehrani F, Behboudi-Gandevani S, Hosseinpanah F, Khalili D, Azizi F. The prevalence and causes of primary infertility in Iran: a population-based study. *Glob J Health Sci* 2015;**7**:226–232.

Keiding N, Ali MM, Eriksson F, Matsaseng T, Toskin I, Kiarie J. The use of time to pregnancy for estimating and monitoring human fecundity from demographic and health surveys. *Epidemiology* 2021;**32**:27–35.

Kirkegaard I, Uldbjerg N, Tabor A, Henriksen TB. Longer time-to-pregnancy in spontaneously conceived pregnancies is associated with lower PAPP-A and free [beta]-hCG in first trimester screening for Down syndrome. *Prenat Diagn* 2014;**34**:235–240.

Klemetti R, Raitanen J, Sihvo S, Saarni S, Koponen P. Infertility, mental disorders and well-being: a nationwide survey. *Acta Obstet Gynecol Scand* 2010;**89**:677–682.

Klouman E, Manongi R, Knut-Inge Klepp. Self-reported and observed female genital cutting in rural Tanzania: associated demographic factors, HIV and sexually transmitted infections. *Trop Med Int Health* 2005;**10**:105–1115.

Kreisel KM, Ikerdeu E, Cash HL, De Jesus SL, Kamb ML, Anderson T, Barrow RY, Sugiyama MS, Basilius K, Madraisau S. An evaluation of infertility among women in the Republic of Palau, 2016. *Hawaii J Health Soc Welf* 2020;**79**:7–15.

Küppers-Chinnow M, Karmaus W. Prävalenz von verminderter Fruchtbarkeit und Inanspruchnahme ärztlicher Hilfe. *Geburtshilfe Frauenheilkd* 1997;**57**:89–95.

Larsen U. Primary and secondary infertility in sub-Saharan Africa. *Int J Epidemiol* 2000;**29**:285–291.

Larsen U. Infertility in central Africa. *Trop Med Int Health* 2003;**8**:354–367.

Larsen U. Research on infertility: which definition should we use? *Fertil Steril* 2005;**83**:846–852.

Louis JF, Thoma ME, Sørensen DN, McLain AC, King RB, Sundaram R, Keiding N, Buck Louis GM. The prevalence of couple infertility in the United States from a male perspective: evidence from a nationally representative sample. *Andrology* 2013;**1**:741–748.

Magnus MC, Fraser A, Rich-Edwards JW, Magnus P, Lawlor DA, Håberg SE. Time-to-pregnancy and risk of cardiovascular disease among men and women. *Eur J Epidemiol* 2021;**36**:383–391.

Mascarenhas MN, Cheung H, Mathers CD, Stevens GA. Measuring infertility in populations: constructing a standard definition for use with demographic and reproductive health surveys. *Popul Health Metr* 2012a;**10**:17.

Mascarenhas MN, Flaxman SR, Boerma T, Vanderpoel S, Stevens GA. National, regional, and global trends in infertility prevalence since 1990: a systematic analysis of 277 health surveys. *PLoS Med* 2012b;**9**:e1001356.

McQuillan J, Greil AL, White L, Jacob MC. Frustrated fertility: infertility and psychological distress among women. *J Marriage Fam* 2003;**65**:1007–1018.

Mena GP, Mielke GI, Brown WJ. Do physical activity, sitting time and body mass index affect fertility over a 15-year period in women? Data from a large population-based cohort study. *Hum Reprod* 2020;**35**:676–683.

Meng Q, Ren A, Zhang L, Liu J, Li Z, Yang Y, Li R, Ma L. Incidence of infertility and risk factors of impaired fecundity among newly married couples in a Chinese population. *Reprod Biomed Online* 2015;**30**:92–100.

Merritt MA, De Pari M, Vitonis AF, Titus LJ, Cramer DW, Terry KL. Reproductive characteristics in relation to ovarian cancer risk by histologic pathways. *Hum Reprod* 2013;**28**:1406–1417.

Miller-Fellows SC, Howard L, Kramer R, Hildebrand V, Furin J, Mutuku FM, Dunstan Mukoko, Ivy JA, King CH. Cross-sectional interview study of fertility, pregnancy, and urogenital schistosomiasis in coastal Kenya: documented treatment in childhood is associated with reduced odds of subfertility among adult women. *PLoS Negl Trop Dis* 2017;**11**:e0006101.

Mirzaei M, Namiranian N, Dehghani Firouzabadi R, Gholami S. The prevalence of infertility in 20-49 years women in Yazd, 2014-2015: a cross-sectional study. *Int J Reprod Biomed* 2018;**16**:683–688.

Muller A, Slama R, Labbé-Declèves C, Jouannet P, Bujan L, Mieusset R, Le Lannou D, Guerin J-F, Benchaib M, Spira A. Geographic variations in probability of pregnancy in four cities of France. *Rev Epidemiol Sante Publique* 2006;**54**:55–60.

Nasrabad HBR, Abbasi-Shavazi MJ, Hosseini-Chavoshi M, Karegar-Shoraki MR. Trend and patterns of childlessness in Iran. *Proceedings of the XXVII International Population Conference of the IUSSP.* 2013; pp. 26–31. Busan, Korea.

Nelson DB, Sammel MD, Patterson F, Lin H, Gracia CR, Freeman EW. Effects of reproductive history on symptoms of menopause: a brief report. *Menopause* 2011;**18**:1143–1148.

Nguyen RH, Wilcox AJ, Skjærven R, Baird DD. Men’s body mass index and infertility. *Hum Reprod* 2007;**22**:2488–2493.

Oakley LL. The epidemiology of infertility: measurement, prevalence and an investigation of early life and reproductive risk factors. 2010; London School of Hygiene & Tropical Medicine.

Passey M, Mgone CS, Lupiwa S, Suve N, Tiwara S, Lupiwa T, Clegg A, Alpers MP. Community based study of sexually transmitted diseases in rural women in the highlands of Papua New Guinea: prevalence and risk factors. *Sex Transm Infect* 1998;**74**:120–127.

Pedersen KK, Hagen C, Eshoj O. Infertility and pregnancy outcome in women with insulin-dependent diabetes mellitus. An epidemiological study. *Ugeskr Laeger* 1994;**156**:6196–6200.

Philippov OS, Radionchenko AA, Bolotova VP, Voronovskaya NI, Potemkina TV. Estimation of the prevalence and causes of infertility in western Siberia. *Bull World Health Organ* 1998;**76**:183–187.

Pick WM, Obermeyer CM. Urbanization, household composition and the reproductive health of women in a South African city. *Soc Sci Med* 1996;**43**:1431–1441.

Polis CB, Cox CM, Tunçalp Ö, McLain AC, Thoma ME. Estimating infertility prevalence in low-to-middle-income countries: an application of a current duration approach to Demographic and Health Survey data. *Hum Reprod* 2017;**32**:1064–1074.

Priestley SR. Impaired fertility in Jamaica: evidence from fertility surveys. *West Indian Med J* 2012;**61**:716–725.

Purkayastha N, Sharma H. Prevalence and potential determinants of primary infertility in India: evidence from Indian demographic health survey. *Clin Epidemiol Glob Health* 2021;**9**:162–170.

Raatikainen K, Harju M, Hippeläinen M, Heinonen S. Prolonged time to pregnancy is associated with a greater risk of adverse outcomes. *Fertil Steril* 2010;**94**:1148–1151.

Rao N, Esber A, Turner A, Mopiwa G, Banda J, Norris A. Infertility and self-rated health among Malawian women. *Women Health* 2018;**58**:1081–1093.

Righarts A, Dickson NP, Ekeroma A, Gray AR, Parkin L, Gillett WR. The burden of infertility in New Zealand: a baseline survey of prevalence and service use. *Aust N Z J Obstet Gynaecol* 2021;**61**:439–447.

Righarts A, Dickson NP, Parkin L, Gillett WR. Infertility and outcomes for infertile women in Otago and Southland. *N Z Med J* 2015;**128**:43–53.

Risch HA, Marrett LD, Howe GR. Parity, contraception, infertility, and the risk of epithelial ovarian cancer. *Am J Epidemiol* 1994;**140**:585–597.

Roode T van, Dickson NP, Righarts AA, Gillett WR. Cumulative incidence of infertility in a New Zealand birth cohort to age 38 by sex and the relationship with family formation. *Fertil Steril* 2015;**103**:1053-1058.e2.

Rostad B, Schmidt L, Sundby J, Schei B. Has fertility declined from mid-1990s to mid-2000s? *Acta Obstet Gynecol Scand* 2013;**92**:1284–1289.

Rutstein SO, Shah IH. *Infecundity, infertility, and childlessness in developing countries* [Internet]. 2004; OCR Macro and World Health Organization. Available from: <https://www.who.int/reproductivehealth/publications/infertility/DHS_9/en/>.

Safarinejad MR. Infertility among couples in a population-based study in Iran: prevalence and associated risk factors. *Int J Androl* 2008;**31**:303–314.

Samarakoon S, Rajapaksa L, Seneviratne HR. Prevalence of primary and secondary infertility in the Colombo District. *Ceylon J Med Sci* 2007;**45**:83–91.

Sarac M, Koc I. Prevalence and risk factors of infertility in Turkey: evidence from demographic and health surveys, 1993-2013. *J Biosoc Sci* 2018;**50**:472–490.

Sharif SN, Azizi Kutenaee M, Darsareh F, Roozbeh N. Prevalence and risk factors of infertility in a southern port city of Iran. *Hormozgan Medical Journal* 2020;**24**: e99412.

Singh BP, Shukla U. Inability to conceive and treatment-seeking behaviour in Uttar Pradesh state in India. *Can Stud Popul* 2015;**42**:1–12.

Slama R, Ducot B, Carstensen L, Lorente C, La Rochebrochard E de, Leridon H, Keiding N, Bouyer J. Feasibility of the current-duration approach to studying human fecundity. *Epidemiology* 2006;**17**:440–449.

Slama R, Hansen OKH, Ducot B, Bohet A, Sorensen D, Giorgis Allemand L, Eijkemans MJC, Rosetta L, Thalabard JC, Keiding N, *et al.* Estimation of the frequency of involuntary infertility on a nation-wide basis. *Hum Reprod* 2012;**27**:1489–1498.

Soares S, Rodrigues T, Barros H. [Infertility prevalence in the city of Porto]. *Acta Med Port* 2011;**24**:699–706.

Somé EN, Boncoungou J, Poda JN. Prevalence of the infertility among couples in Ouagadougou (Burkina Faso): A population-based survey. *Open Public Health J* 2016;**9**:88-97.

Song S. Assessing the impact of in utero exposure to famine on fecundity: evidence from the 1959-61 famine in China. *Popul Stud* 2013;**67**:293–308.

Sundby J, Mboge R, Sonko S. Infertility in the Gambia: frequency and health care seeking. *Soc Sci Med* 1998;**46**:891–899.

Sundby J, Schei B. Infertility and subfertility in Norwegian women aged 40-42: prevalence and risk  factors. *Acta Obstet Gynecol Scand* 1996;**75**:832–837.

Taponen S, Ahonkallio S, Martikainen H, Koivunen R, Ruokonen A, Sovio U, Hartikainen A-L, Pouta A, Laitinen J, King V, *et al.* Prevalence of polycystic ovaries in women with self‐reported symptoms of oligomenorrhoea and/or hirsutism: Northern Finland Birth Cohort 1966 Study. *Hum Reprod* 2004;**19**:1083–1088.

Taylor GM, Faragher EB, Chantler E, Seif MW. Fecundity in the modern city: a comparison of couples attending antenatal clinics in  Manchester (UK) and Melbourne (Australia). *J Obstet Gynaecol* 1999;**19**:489–495.

Terävä A-N, Gissler M, Hemminki E, Luoto R. Infertility and the use of infertility treatments in Finland: prevalence and socio-demographic determinants 1992–2004. *Eur J Obstet Gynecol Reprod Biol* 2008;**136**:61–66.

Thoma ME, McLain AC, Louis JF, King RB, Trumble AC, Sundaram R, Buck Louis GM. Prevalence of infertility in the United States as estimated by the current duration approach and a traditional constructed approach. *Fertil Steril* 2013;**99**:1324–1331.

Toft G, Axmon A, Giwercman A, Thulstrup AM, Rignell-Hydbom A, Pedersen HS, Ludwicki JK, Zvyezday V, Zinchuk A, Spano M, *et al.* Fertility in four regions spanning large contrasts in serum levels of widespread persistent organochlorines: a cross-sectional study. *Environ Health* 2005;**4**:26.

Udgiri R, Patil VV. Comparative study to determine the prevalence and socio-cultural practices of infertility in rural and urban field practice area of tertiary care hospital, Vijayapura, Karnataka. *Indian J Community Med* 2019;**44**:129–133.

Unisa S. Childlessness in Andhra Pradesh, India: treatment-seeking and consequences. *Reprod Health Matters* 1999;**7**:54–64.

Vahidi S, Ardalan A, Mohammad K. Prevalence of primary infertility in the Islamic Republic of Iran in 2004-2005. *Asia Pac J Public Health* 2009;**21**:287–293.

Van der Avoort IAM, Van Golde RJT, Tuerlings JHAM, Kiemeney LA, Meuleman EJH, Braat DDM, Kremer JAM. Underestimation of subfertility among relatives when using a family history: taboo bias. *J Androl* 2003;**24**:285–288.

Walraven G, Scherf C, West B, Ekpo G, et al. The burden of reproductive-organ disease in rural women in the Gambia, West Africa. *Lancet* 2001;**357**:1161–1167.

Wang B, Zhou W, Zhu W, Chen L, Wang W, Tian Y, Shen L, Zhang J. Associations of female exposure to bisphenol A with fecundability: evidence from a preconception cohort study. *Environ Int* 2018;**117**:139–145.

Weiss HA, Troisi R, Rossing MA, Brogan D, Coates RJ, Gammon MD, Potischman N, Swanson CA, Brinton LA. Fertility problems and breast cancer risk in young women: a case-control study in the United States. *Cancer Causes Control* 1998;**9**:331–339.

Woodall PA, Kramer MR. Schistosomiasis and infertility in East Africa. *Am J Trop Med Hyg* 2018;**98**:1137.

Wu S, Tian J, Wang M, Pan B, Lü H, Wang Z, Li H. [The effect of cadmium pollution on reproductive health in females]. *Zhonghua Liu Xing Bing Xue Za Zhi* 2004;**25**:852–855.

Wulff M, Högberg U, Stenlund H. Infertility in an industrial setting: a population-based study from Northern Sweden. *Acta Obstet Gynecol Scand* 1997;**76**:673–679.

Xingping G, Yu W, Qiufang H. Prevalence of infertility in rural areas of Shanxi Province. *Chin J Fam Plan* 2006;**14**:358.

Yang F, Li L, Chen J-P, Liu X-Q, Zhong C-L, Yang Y, Ren Y-F, Yuan W, Liang H, Miao M-H. Couple’s infertility in relation to male smoking in a Chinese rural area. *Asian J Androl* 2017;**19**:311–315.

Yang Y-Q, Shen H, Chen J, Chen Z-W. [A prevalence survey of infertility in Beijing, China]. *Zhonghua Yi Xue Za Zhi* 2011;**91**:313–316.

Zargar AH, Wani AI, Masoodi SR, Laway BA, Salahuddin M. Epidemiologic and etiologic aspects of primary infertility in the Kashmir region of India. *Fertil Steril* 1997;**68**:637–643.

Zhang H, Wang S, Zhang S, Wang T, Deng X. Increasing trend of prevalence of infertility in Beijing. *Chin Med J* 2014;**127**:691–695.

Zhang X -h, Zhang R. Analysis on infertility prevalence and its affecting factors in Gansu province. *Reprod Contracept* 2013;**33**:184–192.

Zhou Z, Zheng D, Wu H, Li R, Xu S, Kang Y, Cao Y, Chen X, Zhu Y, Chen Z, *et al.* Epidemiology of infertility in China: a population-based study. *BJOG* 2018;**125**:432–441.

# 
